# Chemo-/Regio-Selective Synthesis of Novel Functionalized Spiro[pyrrolidine-2,3′-oxindoles] under Microwave Irradiation and Their Anticancer Activity

**DOI:** 10.3390/molecules28186503

**Published:** 2023-09-07

**Authors:** Richa Sharma, Lalit Yadav, Ali Adnan Nasim, Ravi Kant Yadav, Rui Hong Chen, Neha Kumari, Fan Ruiqi, Ashoke Sharon, Nawal Kishore Sahu, Sirish Kumar Ippagunta, Paolo Coghi, Vincent Kam Wai Wong, Sandeep Chaudhary

**Affiliations:** 1 Laboratory of Organic and Medicinal Chemistry (OMC Lab), Department of Chemistry, Malaviya National Institute of Technology, Jawaharlal Nehru Marg, Jaipur 302017, Rajasthan, India; richasharmachem1@gmail.com (R.S.); lalityadav50@gmail.com (L.Y.); ravisubhashyadav@gmail.com (R.K.Y.); nawal.chemistry@gmail.com (N.K.S.); 2 State Key Laboratory of Quality Research in Chinese Medicine, Macau University of Science and Technology, Avenida Wai Long, Taipa, Macau 999078, China; aanasim@gmail.com (A.A.N.); guisitong2@hotmail.com (R.H.C.); 19098533c111003@student.must.edu.mo (F.R.); 3 Department of Applied Chemistry, Birla Institute of Technology Mesra, Ranchi 835215, Jharkhand, India; nehacelia.kumari94@gmail.com (N.K.); asharon@bitmesra.ac.in (A.S.); 4 Department of Chemistry, Government Engineering College, Bharatpur 321303, Rajasthan, India; 5 Department of Biotechnology, All India Institute of Medical Sciences (AIIMS), New Delhi 110029, India; sirish.ippagunta@gmail.com; 6 School of Pharmacy, Macau University of Science and Technology, Avenida Wai Long, Taipa, Macau 999078, China; 7 Dr. Neher’s Biophysics Laboratory for Innovative Drug Discovery, State Key Laboratory of Quality Research in Chinese Medicine, Macau University of Science and Technology, Avenida Wai Long, Taipa, Macau 999078, China; 8 Laboratory of Bioactive Heterocycles and Catalysis (BHC Lab), Department of Medicinal Chemistry, National Institute of Pharmaceutical Education and Research-Raebareli (Transit Campus), Bijnor–Sisendi Road, Near CRPF Base Camp, Sarojini Nagar, Lucknow 226002, Uttar Pradesh, India

**Keywords:** anticancer, spirooxindole, [3+2] cycloaddition, azomethine ylide, microwave

## Abstract

A novel series of nitrostyrene-based spirooxindoles were synthesized via the reaction of substituted isatins **1a**–**b**, a number of α-amino acids **2a**–**e** and (E)-2-aryl-1-nitroethenes **3a**–**e** in a chemo/regio-selective manner using [3+2] cycloaddition (Huisgen) reaction under microwave irradiation conditions. The structure elucidation of all the synthesized spirooxindoles were done using ^1^H and ^13^C NMR and HRMS spectral analysis. The single crystal X-ray crystallographic study of compound **4l** was used to assign the stereochemical arrangements of the groups around the pyrrolidine ring in spiro[pyrrolidine-2,3′-oxindoles] skeleton. The in vitro anticancer activity of spiro[pyrrolidine-2,3′-oxindoles] analogs **4a**–**w** against human lung (A549) and liver (HepG2) cancer cell lines along with immortalized normal lung (BEAS-2B) and liver (LO2) cell lines shows promising results. Out of the 23 synthesized spiro[pyrrolidine-2,3′-oxindoles], while five compounds (**4c**, **4f**, **4m**, **4q**, **4t**) (IC_50_ = 34.99–47.92 µM; SI = 0.96–2.43) displayed significant in vitro anticancer activity against human lung (A549) cancer cell lines, six compounds (**4c**, **4f**, **4k**, **4m**, **4q**, **4t**) (IC_50_ = 41.56–86.53 µM; SI = 0.49–0.99) displayed promising in vitro anticancer activity against human liver (HepG2) cancer cell lines. In the case of lung (A549) cancer cell lines, these compounds were recognized to be more efficient and selective than standard reference artemisinin (IC_50_ = 100 µM) and chloroquine (IC_50_ = 100 µM; SI: 0.03). However, none of them were found to be active as compared to artesunic acid [IC_50_ = 9.85 µM; SI = 0.76 against lung (A549) cancer cell line and IC_50_ = 4.09 µM; SI = 2.01 against liver (HepG2) cancer cell line].

## 1. Introduction

In the search for a tool to synthesize a variety of pharmaceutically important heterocycles, one methodology that conceivably meets all the goals and gives a better alternative for library creation of heterocycles is the “multicomponent reaction” pathway. Moreover, it introduces the complexity, regiospecificity and stereoselectivity in the minimum number of steps [1,2,3]. The combination of all these properties along with the very quick reaction kinetics of microwave contributes new strategies to the fast and productive combination of heterocycles. Since the development of precisely regulated microwave reactors, microwave-assisted organic synthesis (MAOS) has efficiently revolutionized synthetic heterocyclic chemistry. The microwave reactor has been utilized to quicken the reaction rate along with the enhancement in the yields of the desired product, shortening the reaction time and upgrading the selectiveness of the reaction [4,5,6]. In this sequence, microwave-assisted synthesis of a series of distinctive nitrostyrene-based spirooxindoles as a potent anticancer agent has been done against human lung (A549) and liver (HepG2) cancer cell lines along with immortalized normal lung (BEAS-2B) and liver (LO2) cell lines.

Cancer is the primary cause of death in both more and less developed countries and the burden is anticipated to increase globally owing to population growth and aging. According to estimates, approximately 19.3 million new cancer diagnoses and 10.0 million casualties happened globally in 2020 based on GLOBACON estimates [7]. Cancer is an abnormal development of cells that, in general, multiply in an uncontrolled manner. For several years, lung cancer has been the most frequently diagnosed and the prime cause of death in men and the second major cause of casualties in women [8]. Liver cancer is the sixth most common cancer globally. In addition, previous studies from the year 2020 depicted that approx. 905,677 people were diagnosed with liver cancer worldwide. Out of these, 830,200 people died from liver cancer throughout the world. By 2040, there may be a >55% increase in the number of new liver cancer cases and fatalities [9]. Numerous natural and synthetic anticancer drugs are accessible in the market (cisplatin and paclitaxel); nevertheless, such pharmaceutical products are often associated with unfortunate and undesirable side effects [10]. Hence, the significance of investigating new prominent pharmaceutically important skeletons as anticancer agents through easily accessible starting materials becomes a crucial step in improving the success rates in cancer treatment.

**Figure 1:**
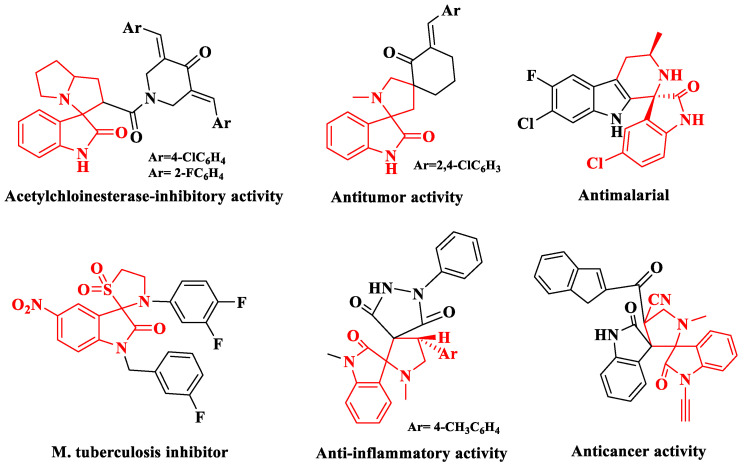
Chemical structure of various biologically active compounds having spiro(pyrrolidine-2,3′-oxindole) as core skeleton.

Spirooxindoles, specifically the spiro(pyrrolidine-2,3′-oxindole) core as a ubiquitous pharmaceutically privileged heterocyclic moiety, are blended with numerous significant biological activities (Figure 1) such as anticancer [11,12,13,14,15], antimicrobial [16,17,18,19], anti-inflammatory [20,21], antimycobacterial [22], acetylcholinesterase-inhibitory [23,24], MDM2-p53 interaction inhibitor [25], p38alpha inhibitor [26], *β*-secretase (BACE1) inhibitory activity [27], antitumor [28], antitubercular [29], antimalarial [30], etc. These spiro molecules are the key skeleton of many bioactive natural/synthetic scaffolds such as horsfiline [31,32,33,34,35], MI-219 [36], rhynchophylline and isorhynchophylline [37], mitraphylline [38], elacomine [39], alstonine [40], spiro-tryprostatine A and B [41,42], etc. In view of these facts, spirooxindoles can be described as being on the verge of becoming a new anticancer lead molecule because similar skeleton-based lead molecules such as MI-888 and NITD609 are now in preclinical assessments for the treatment of malaria and cancer growth individually [43,44]. Therefore, inspired by MI-888 and MI-219, which contain an oxindole substructure, i.e.; spiro(pyrrolidine-2,3′-oxindole) motifs with a spiro pyrrolidine ring having nitrogen at N3 position and as inhibitors of MDM2–p53 are potential anti-breast cancer agents; herein, we present the design, and synthesize and evaluate the in vitro anticancer activity of a novel series of nitrostyrene-based spirooxindoles in which the oxindole core with spiro pyrrolidine ring has nitrogen in the N2 location, i.e., spiro(pyrrolidine-2,3′-oxindole) as the key pharmacophore.

**Figure 2:**
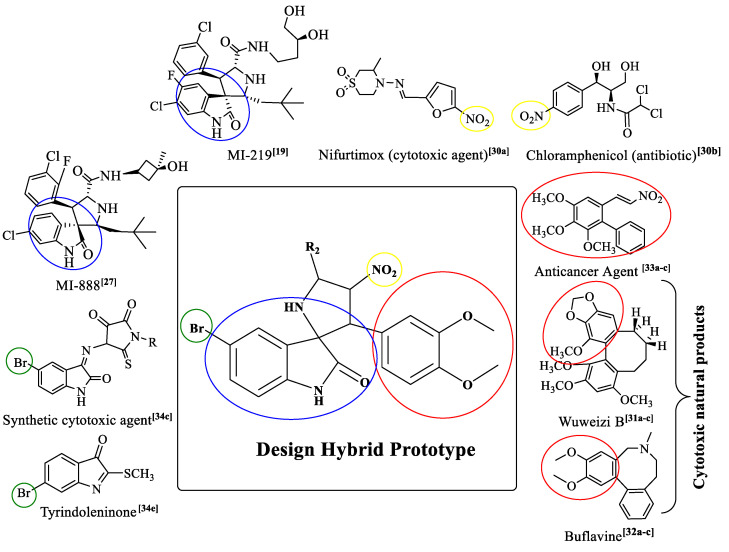
Design strategy of nitrostyrene–based novel spirooxindoles.

**Scheme 1:**
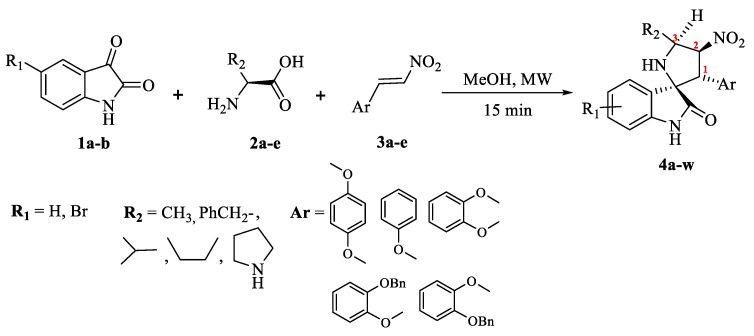
Our approach: Synthesis of nitrostyrene–based spirooxindole derivatives **4a**–**w**.

Nitroaromatic substructure has been identified in several literature studies as a potentially effective cytotoxic agent, either in part or as a whole [44,45,46,47]. The reduction of the nitro group, subsequently followed by the interaction of the active intermediate species (nitro-radical anions) with DNA, is the primary cause of the biological activity of nitro derivatives [48]. Various reports depict that the introduction of a nitro group on bioactive moieties enhances their biological activity [49,50,51,52,53]. However, in this sequence, inspired by the naturally and synthetically occurring cytotoxic and anticancer pharmacophore, i.e.; Wuweizi B [54,55,56], Buflavine [57,58,59] and nitrovinylstilbenes [60,61,62], respectively, we envisaged that the hybrid of donating group substituting nitroaromatic system with spirooxindole core moiety could lead to a further increase in cytotoxicity and, thus, increase the anticancer activity. The purpose of taking the bromo derivative of isatin can be justified by the fact that it carries a variety of biological activities including anticancer ones and the spiro derivatives of bromoisatin also have antiphlogistic and analgesic activities. Moreover, taking into account the cytotoxic activity of naturally occurring tyrindoleninone, we decided to add such a kind of bromo derivative of isatin into our design methodology [63,64,65,66,67]. Therefore, considering all the facts, we designed a hybrid prototype (Figure 2). Although there are only a few reports available in the literature on nitrostyrene-based spirooxindoles [68,69,70,71,72], and several types of spirooxindoles have also been highlighted, only a few of them emphasized their anticancer activity [73,74,75,76,77,78,79]. Herein, in pursuit of a new anticancer agent using our designed prototype, for the first time we report the synthesis and in vitro anticancer activities of twenty-three various nitrostyrene-based spirooxindole derivatives **4a**–**w** against human lung (A549) and liver (HepG2) cancer cell lines along with immortalized normal lung (BEAS-2B) and liver (LO2) cell lines. The anticancer drugs artemisinin, chloroquine, artesunic acid and Paclitaxel were taken as standard reference. The stereochemistry of the target compound was confirmed by X-ray crystal analysis (Scheme 1).

## 2. Results and Discussion

### 2.1. Chemistry

#### 2.1.1. Synthesis

Our initial investigation was aimed towards the development of a sustainable and an environmentally benign economic protocol for the synthesis of spiro[pyrrolidine-2,3′-oxindoles]. We started our optimization study by utilizing the multicomponent reaction of isatin **1a**, L-alanine **2b** and (E)-1,4-dimethoxy-2-(2-nitrovinyl)benzene **3a** as a starting substrate (Table 1) under conventional heating as well as under microwave irradiation conditions. The study involving conventional heating furnished the desired **4b** from the reaction of **1a**, **2b** and **3a** in up to a maximum 65% yield at r.t to 100 °C temperature range and in 120–240 min time range (Table 1, entry 1–14). Then, we switched our attention towards microwave-assisted optimization study for the synthesis of spiro[pyrrolidine-2,3′-oxindoles]. Hitherto, it is widely mentioned that water can be an excellent solvent for the synthesis of spirooxindole [80,81,82]. Therefore, we started our investigation by the reaction of an equimolar amount of isatin **1a**, L-alanine **2b** and (E)-1,4-dimethoxy-2-(2-nitrovinyl)benzene **3a** in aqueous medium at room temperature for 30 min under microwave irradiation. Unfortunately, **4b** was obtained only in a trace amount (Table 1, entry 1), whereas reducing the time of microwave to 20 and 10 min does not generate the product **4b** at all (Table 1, entries 2–3). Furthermore, it is also reported that polar solvents such as EtOH, MeOH and/or their aqueous mixture have been used for the synthesis of spirooxindole [11,12,13,14,15]. Consequently, we decided to take an equimolar amount of isatin **1a**, L-alanine **2b** and (E)-1,4-dimethoxy-2-(2-nitrovinyl)benzene **3a** dissolved in equal ratio of MeOH: H_2_O (1:1) at a temperature of 60 °C, 90 °C and 120 °C for 15, 10 and 5 min under microwave irradiation, respectively. However, intriguingly, it furnished **4b** in 66%, 62% and 48% yields, respectively (Table 1, entries 4–6). Keeping all the other reaction conditions the same, when EtOH was used instead of MeOH along with an equal ratio of water at a temperature of 60 °C, 90 °C and 120 °C for 15, 10 and 5 min under microwave irradiations, respectively, **4b** was obtained in 59%, 49% and 36% yields, respectively (Table 1, entries 10–12). It was confirmed that when using water, either alone or in a mixture with polar solvents, the reaction does not give fruitful results. Thus, we decided to use only polar solvents in our further optimization studies. In the quest for an effectual result, keeping all the other reaction conditions the same at a temperature of 60 °C for 15, 10 and 5 min, respectively, **4b** was obtained in 91%, 82% and 61% yields, respectively (Table 1, entries 7–9). Subsequently, keeping all the reaction variables the same, the effect of variation in the temperature was examined. It was observed that increasing the temperature up to 90 °C and 120 °C does not have any beneficial effect on the yield of the reaction (Table 1, entries 13–14). Thus, overall, an equimolar amount of isatin **1a**, L-alanine **2b** and (E)-1,4-dimethoxy-2-(2-nitrovinyl)benzene **3a** dissolved in methanol as solvent at 60 °C for 15 min under microwave irradiation was found to be the best optimized reaction condition.

#### 2.1.2. Substrate Scope from the Reaction of Isatin **1a**–**b** with Differently Substituted Amino Acids **2a**–**e** and Nitrostyrenes **3a**–**e** to Furnish Spiro[pyrrolidine-2,3′-oxindoles] **4a**–**w** Derivatives

**Scheme 2:**
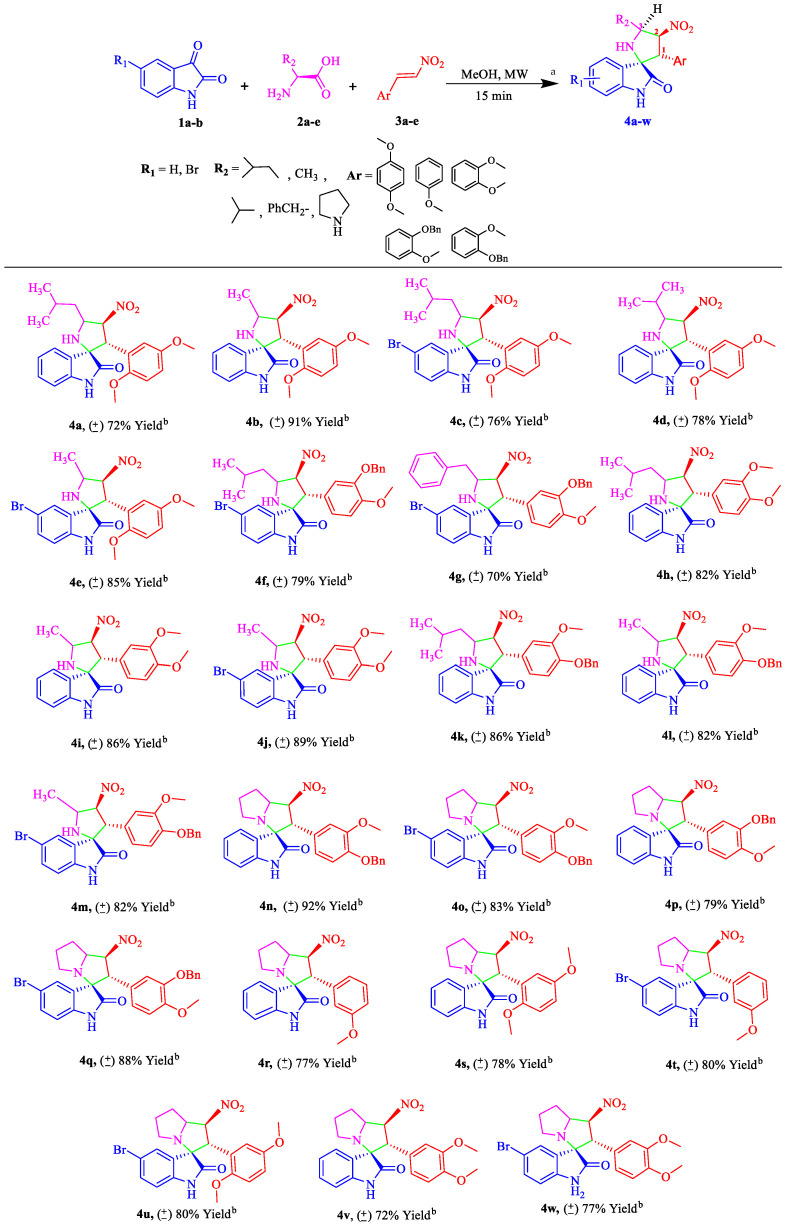
^a^ Reaction Conditions: Substituted isatins **1a**–**b** (0.5 mmol), various amino acids (0.5 mmol and nitrostyrenes **3a**–**e** dissolved in methanol as solvent at 60 °C for 15 min under microwave irradiation. ^b^ Combined yield of isolated mixture of regioisomers.

With the optimized reaction conditions in hand, we next explored the substrate scope utilizing substituted isatins, numerous amino acids and a variety of nitrostyrenes. Initially, to investigate the effect of the increment in the carbon chain of amino acids on the yield of the reaction, isatin/5-bromo isatin **1a**–**b** and various amino acids **2a**–**e** were allowed to react with E-1,4-dimethoxy-2-(2-nitrovinyl)benzene **3a** using the optimized reaction conditions (Scheme 2). One carbon containing amino acids, i.e., in the case of alanine **2b**, when reacted with isatin and 5-bromo isatin **1a**–**b**, it furnished the products **4b** and **4e** in excellent (±) 91% and 85% combined yields, respectively. 

However, in the case of leucine **2a** having four carbon atoms, when reacted with isatin/5-bromo isatin **1a**–**b** and (E)-1,4-dimethoxy-2-(2-nitrovinyl)benzene **3a**, it furnished the products **4a** and **4c** in (±) 72% and 76% combined yields, respectively. This indicated that with an increase in the carbon chain, a slight decrement in the yields of the reaction was observed. Additionally, valine was found to be in good agreement with the optimized reaction conditions and afforded **4d** in (±) 78% combined yield. Subsequently, different substituents at various positions of the aryl group of 2π-component work quite efficiently. 5-bromo-isatin **1b** with (E)-2-(benzyloxy)-1-methoxy-4-(2-nitrovinyl)benzene **3b** underwent reaction with leucine and phenylalanine under the optimized reaction conditions and furnished the corresponding products **4f** and **4g** in (±) 79% and 70% combined yields, respectively. It is noteworthy here that a remarkable increment in the reaction yield has been observed when a secondary amino acid, i.e., proline **2e** has been used under the optimized conditions with 5-bromo isatin/ isatin along with (E)-2-(benzyloxy)-1-methoxy-4-(2-nitrovinyl)benzene **3b** and afforded **4p** and **4q** in (±) 79% and 88% combined yields, respectively. Furthermore, no significant changes in the reaction yield have been observed by changing the position of methoxy groups in the 2π-component, i.e., in the case of (E)-1,2-dimethoxy-4-(2-nitrovinyl)benzene **3c**, the products **4h**, **4i** and **4j** have been obtained in (±) 82%, 86% and 89% good combined yields, respectively. The utilization of only donating group substituted nitrostyrenes can be justified by the virtue of naturally and synthetically occurring cytotoxic and anticancer pharmacophores, i.e., Wuweizi B [54,55,56], Buflavine [57,58,59] and nitrovinylstilbenes [60,61,62]. In continuation, the change in the position of O-benzylic group and methoxy group on nitrostyrenes was also found to be well tolerated with our optimized reaction conditions. (E)-1-(benzyloxy)-2-methoxy-4-(2-nitrovinyl)benzene **3d** was reacted with isatin/ 5-bromo isatin **1a**–**b** and various amino acids leucine **2a**, alanine **2b**, and proline **2e**, thus profoundly furnishing the corresponding products **4k**–**o** in (±) 86%, 82%, 82%, 92% and 83% combined yields, respectively. Mono-group (-OCH_3_) substituted nitrostyrene, i.e., (E)-1-methoxy-3-(2-nitrovinyl)benzene **3e** also works efficiently with the optimized parameters and affords the corresponding spiro[pyrrolidine-2,3′-oxindole] analogs **4r** and **4t** in (±) 77% and 80% combined yields, respectively. In addition, the reactions of (E)-1,4-dimethoxy-2-(2-nitrovinyl)benzene **3a** and (E)-1,2-dimethoxy-4-(2-nitrovinyl)benzene **3c** were reacted with proline **2e** and isatin/5-bromo isatin **1a**–**b** using the optimized parameters and furnished **4s**, **4u**, **4v** and **4w** in (±) 78%, 80%, 72% and 77% combined yields, respectively. Consequently, based on the above facts, it has been interpreted that the bromo-substituted isatin was found to be more favourable towards the optimized reaction condition as compared to the unsubstituted one; therefore, it generated the corresponding product in relatively good yields. In addition, the secondary cyclic amino acid was efficient at generating the desired product in excellent yields compared to its counterpart (aliphatic amino acids). To conclude, we have synthesized twenty-three novel nitrostyrene-based spirooxindoles in good to excellent yields.

#### 2.1.3. Single Crystal X-ray Diffraction Studies

**Figure 3:**
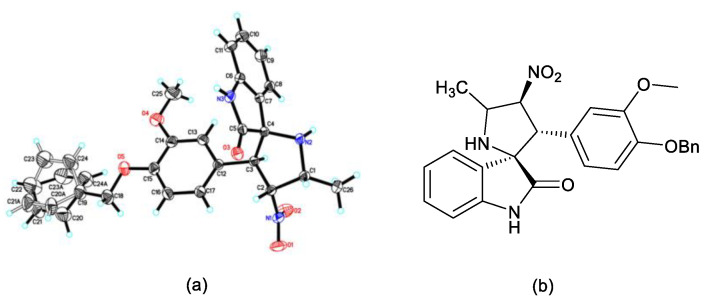
(**a**) ORTEP diagram of the cycloadduct **4l**. (**b**) Structure of 3′-(4-(benzyloxy)-3-methoxyphenyl)-5′-methyl-4′-nitrospiro[indoline-3,2′-pyrrolidin]-2-one **4l** [83,84,85].

Finally, the stereochemistry of the novel synthesized spiro[pyrrolidine-2,3′-oxindole] analogs via [1,3] dipolar cycloaddition (Huisgen) reaction was unequivocally determined by the single crystal X-ray diffraction analysis of the cycloadduct **4l** (Figure S3, Page S1–S2 of SI). A suitable single crystal of compound **4l** was prepared and the diffraction data were collected on a Rigaku Oxford Diffractometer. The OLEX2 [83,84] program was used to solve the structure using direct methods and refined with the ShelXL [85] refinement package using least squares minimization. The single crystal X-ray analysis confirmed all the stereochemistry associated and, overall, the three-dimensional structure of the compound **4l**. Figure 3 represents an ORTEP diagram drawn with 30% probability displacement ellipsoids. The phenyl ring is found to be disordered due to the presence of two major rotamers [C19, C20, C21, C22, C23 C24 and C19, C20A, C21A, C22, C23A, C24A]. Along with single crystal X-ray data of **4l**, the structures of spiro[pyrrolidine-2,3′-oxindole] have been confirmed by ^1^H and ^13^C NMR and HRMS spectroscopic techniques. The full characterization data are reported in the Materials and Methods section; in addition, the ^1^H and ^13^C NMR spectral data of **4a**–**w** are given in Supplementary Materials. 

#### 2.1.4. Plausible Mechanism

**Scheme 3:**
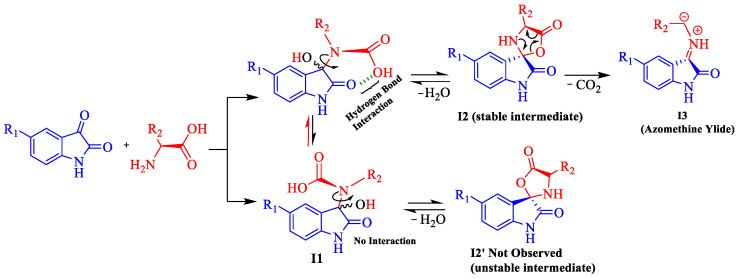
Possible intermediate **I2** and **I2′** before the formation of azomethine ylide.

It has been well documented that the spirooxindole class of bioheterocycles possesses a wide array of biological activity. However, we realized during our investigation that nitrostyrene-based spirooxindoles have never been subjected to testing of their anticancer activity in spite of having a nitro group which is responsible for cytotoxicity [45,46,47]. Therefore, in our ongoing search for a novel anticancer agent, we have synthesized 23 novel amino acid-nitrostyrene-based spirooxindoles by utilizing the literature procedure [72]. Additionally, with reference to the literature [70,72], the plausible mechanism of the regio-/stereoselective product can be depicted starting from the stereochemistry of the four stereocenters that can possibly have eight pairs of promising diastereoisomers, but the predefined stereochemistry of amino acid and trans β-nitrostyrene will determine the stereochemistry of C-1, C-2 and C-3 in the product. Subsequently, the non-appearance of six diastereoisomer pairs has been anticipated due to two reasons: (i) the hydrogen at C-3 cannot have a symmetry that is above the plane; and (ii) there is no possibility of the substituents at C-1 and C-2 to attain Z-configuration. Considering the stereochemistry of the quaternary carbon, previous reports [70,72] depicted how the reaction between isatin and amino acid give rise to the formation of two possible intermediates. i.e., **I2** and **I2′**; interestingly, out of these two, only one intermediate **I2** is highly stable and observed in our case of regioselective product. The probable reason can be justified from the hydrogen-bond interaction influenced by the carbonyl group present on isatin and so, we get one intermediate exclusively (Scheme 3). 

Once the stereochemistry of the quaternary stereocenter has been determined, the potential development of an extra pair of diastereoisomers is dismissed and the following errand was to decide the stereochemistry of the substituents at positions C-1′ and C-2′ of cycloadduct **4**, whose stereoselectivity will be resolved by the [3+2] cycloaddition between the azomethine ylide **I3** (Scheme 3) and trans β-nitrostyrene **3**. The key factor here is the ylide structure because of its cyclic nature which gives a fairly inflexible ring layout and brings about a superior diastereofacial approach between the ylide and the β-nitrostyrene.

**Scheme 4:**
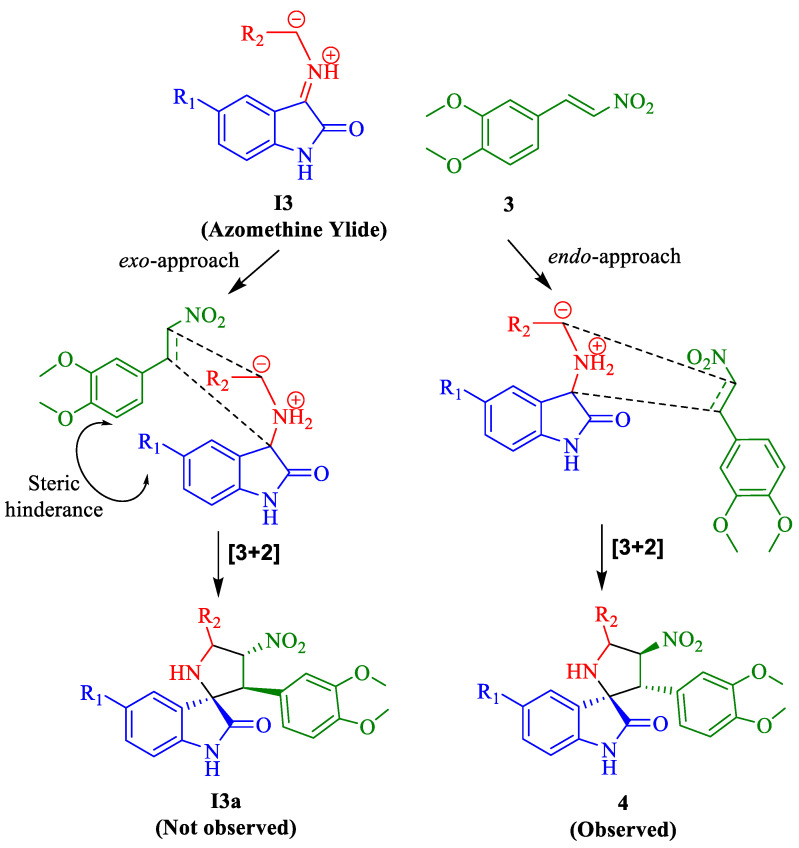
Probable reason for the formation of single regioisomer of spiro[pyrrolidine-2,3′-oxindoles] during our one-pot [1,3]-dipolar cycloaddition reaction.

The approach between the two can be interpreted in two different ways. We start with the exo approach which portrays that the steric hindrance between the basic isatin skeleton and cumbersome trans β-phenyl nitrostyrene brings about an electrostatic aversion within these components resulting in an increase in the free energy of activation for that particular transition state whereas the trans β-phenyl nitrostyrene will adequately escape from the isatin moiety in the endo approach and thus form a stable transition state which has a low value of free energy of activation. Therefore, cycloadduct 4 will be profoundly observed through the endo-approach pathway while the stereoisomer **I3a**, obtained from the exo-approach, was definitely not found in our reaction (Scheme 4).

### 2.2. In Vitro Anticancer Activity against Lung/Liver Cancer and Normal Cell Lines

All the synthesized derivatives of spiro[pyrrolidine-2,3′-oxindoles] **4a**–**w** have been evaluated for their in vitro cytotoxic activities against liver and lung cancer cell lines as well as immortalized normal cell lines. While human lung cancer (A549) cell lines were compared to immortalized (BEAS-2B) normal cell lines, the liver cancer was scrutinized by using HepG2 cancer liver cell lines versus LO2 normal liver cell lines. The study used artemisinin, artesunic acid, chloroquine and paclitaxel as standard reference drugs (Table 2 and Table 3, Supplementary Materials Figures S1–S4).

#### 2.2.1. Structure–Activity Relationship [Anticancer Activity against Lung Cancer (A549) Cell Line]

We have synthesized a series of spiro[pyrrolidine-2,3′-oxindole] analogs (**4a**–**w**). The nitrostyrenes substituted with various electron-donating, containing (*EDGs*, such as *OMe*-/*Me*-/ *2,4-(OMe)_2_*) at the varying positions (*o-*/*m-*/*p-*) reacted with several amino acids along with isatin/5-bromoisatin. The minimum inhibitory concentration (MIC) values for all the spiro[pyrrolidine-2,3′-oxindole] analogs (**4a**–**w)** were determined against the lung (A549) cancer cell lines. As can be seen from Table 3, five compounds (**4c**, **4f**, **4m**, **4q**, **4t**) (IC_50_ = 34.99–47.92 µM; SI = 0.96–2.43) displayed significant in vitro anticancer activity against human lung (A549) cancer cell lines. These compounds were recognized as more efficient and selective than standard reference artemisinin (IC_50_ = 100 µM) and chloroquine (IC_50_ = 100 µM; SI: 0.03). However, none of them were found to be active as compared to artesunic acid [IC_50_ = 9.85 µM; SI = 0.76 against lung (A549) cancer cell line]. Compounds **4a**–**b**, **4d**, **4g**–**l**, **4n**–**p**, **4r**–**s**, and **4u**–**w** were found inactive against lung (A549) cancer cell lines.

It has been concluded that the bromo-substituted isatin was found to be more fruitful in comparison to the unsubstituted isatin. Subsequently, amino acids such as leucine, alanine and proline were blended and helped to enhance the anticancer activity of corresponding compounds. While talking about the effects of various groups on the anticancer activity against the lung (A549) cancer cell lines, the 1,4-dimethoxynitrostyrene and (E)-2-(benzyloxy)-1-methoxy-4-(2-nitrovinyl)benzene were the only two nitroaromatic styrenes that enhanced the anticancer activity of the spirooxindole derivatives. Out of these few selective spirooxindole derivatives, compound **4c** was found to be the most active of the series with the IC_50_ value of 34.99 µM against lung A549 cancer cell lines with a selectivity index of 0.96 (Table 2, entry 3), whereas compounds **4f**, **4m**, **4q**, and **4t** exhibited equally comparable results to those of compound **4c** and showed an IC_50_ value of 41.12, 45.94, 47.92 and 45.22 µM, respectively (Table 2, entries 6, 13, 17, 20). Although the IC_50_ values of these derivatives were slightly lower, they had a better selectivity index than compound **4c**, which is 2.43, 1.38, 2.09 and 1.50, respectively. The derivative **4e** was the least active out of these six having an IC_50_ value of 85.63 µM and a selectivity index of 1.17 (Table 2, entry 5). Moreover, these compounds were found to be more active than artemisinin and chloroquine. They were quite potent and comparable with artesunate and showed these promising activities. However, all the compounds were found to have a less promising activity profile compared to the standard drug paclitaxel.

#### 2.2.2. Structure–Activity Relationship [Anticancer Activity against Liver Cancer (HepG2) Cell Line]

The in vitro anticancer evaluation against liver cancer (HepG2) cells followed a similar pattern as that of the activity pattern observed against the lung (A549) cancer cell lines. Six synthetic spiro[pyrrolidine-2,3′-oxindole] analogs (**4c**, **4f**, **4k**, **4m**, **4q**, and **4t**) showed higher cytotoxic potency and selectivity than the reference drug, artemisinin (IC_50_ = >100 μM) and equally comparable with chloroquine (IC_50_ = 49.02 μM; SI: 0.30). The IC_50_ values for these derivatives were 60.28, 81.95, 67.76, 41.56, 85.63 and 46.05 μM, respectively (Table 3, entries 3, 6, 11, 13, 17, 20). These compounds were recognized as being more efficient and more selective than standard reference artemisinin (IC_50_ = >100 µM) and comparable to chloroquine (IC_50_ = 49.02 ± 0.4 µM; SI: 0.30). However, none of them were found to be active compared to artesunic acid [IC_50_ = 4.09 µM; SI = 2.01 against the liver (HepG2) cancer cell line].

In terms of the factors affecting the cytotoxicity of the prepared substrates, it almost follows a similar pattern as that against the lung cancer cell lines but the values of minimum inhibitory concentration (MIC) come out to be slightly higher than the previous cancer cell lines. Out of these six derivatives, compound **4m** exhibited the most promising anticancer activity. Unlike the lung cancer cell lines, six derivatives showed higher IC_50_ values against liver cancer cells but also have relatively lower selectivity index (SI) values. This signifies that the prepared spirooxindole derivatives were more efficient against lung cancer cell lines than liver cancer cell lines.

## 3. Materials and Methods

### 3.1. General Information

Oven-dried laboratory glassware was used for all the synthetic procedures. Melting points were taken in open capillaries on a Sisco melting point apparatus and are presented uncorrected. ^1^H NMR and ^13^C NMR spectra were recorded on a JEOL ECS-400 spectrometer (2-channel console with a flexible broadband RF performance), which was operating at 400 MHz for ^1^H and 100 MHz for ^13^C NMR and utilized CDCl_3_ as a solvent for all the sample preparations. Tetramethylsilane (δ 0.00 ppm) and CDCl_3_ both were served as an internal standard in ^1^H NMR (δ 7.246 ppm) and ^13^C (δ 77.0 ppm) NMR. Patterns of chemical shifts were reported in parts per million. Peak splitting patterns were described as singlet (s), broad singlet (brs), doublet (d), double doublet (dd), triplet (t) and multiplet (m). Coupling constants (J) were reported in Hertz (Hz). High-Resolution Electron Impact Mass Spectra (HR-EIMS) were obtained on Xevo G2-S Q-Tof (Waters, Milford, MA, USA) compatible with ACQUITY UPLC**^®^** and nano ACQUITY UPLC**^®^** systems. Column chromatography was performed over normal (particle size: 60–120 Mesh, 100–200 Mesh) and flash (particle size: 230–400 Mesh) silica gel, which were procured from QualigensTM (Mumbai, India), Rankem (Haryana, India), and Spectrochem (Mumbai, India). TLC plates coated with silica gel (Kiesel 60-F254, Merck (Bengaluru, India) were used for the monitoring of the progress of the reactions. Visualizing agents used for TLC were UV light. BUCHI’s Rotavapor R-210 was used for all drying and concentration procedures. All the analytical-grade supplied solvents such as MeOH and EtOH were used without further purification. All the remaining chemicals and reagents obtained from Sigma Aldrich (Bangalore, India), or Merck (Bengaluru, India) and TCI (Chennai, India) were used without further purification.

### 3.2. General Procedure for the Synthesis of (E)-2-Aryl-1-nitroethenes *(**3a**–**e**)*

**Scheme 5:**
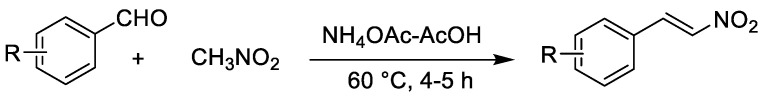
Synthesis of (E)-2-aryl-1-nitroethenes (**3a**–**e**).

The substituted benzaldehyde (1.0 mmol) and nitromethane (5.0 mmol) were added dissolved in the glacial acetic acid. The ammonium acetate (2.5 mmol) was added into the reaction mixture and the whole reaction mixture was sonicated at 60 °C for 4–5 h (Scheme 5) [86]. After the completion of the reaction mixture, the reaction mixture was concentrated under reduced pressure using rotavapor to get the crude mixture, which was further purified through silica-gel column chromatography using the ethyl acetate/hexane (03:97 as an eluent to furnish the pure product **3a**–**e** in 82–85% yield as a yellow solid.

### 3.3. General Procedure for the Synthesis of Spiro[pyrrolidine-2,3′-oxindoles] *(**4a**–**w**)*

Initially, (E)-2-aryl-1-nitroethenes was prepared using the literature procedure [86]. An oven-dried reaction vial was charged with (E)-2-aryl-1-nitroethenes (0.5 mmol), isatin (0.5 mmol) and amino acid (0.5 mmol) followed by dry MeOH (2 mL). The reaction mixture was heated to 60 °C in microwave for 15 min. The reaction mixture was concentrated under vacuo to get a crude compound which was further purified by silica-gel (100–200 mesh) column chromatography using ethyl acetate/hexane as an eluent which furnished the desired product **4a**–**w** in a 70–92% combined yield of isolated mixture of regioisomers.

### 3.4. Characterization Data of Spiro[pyrrolidine-2,3′-oxindoles] *(**4a**–**w**)*

**Compound 4a: 3′-(2,5-dimethoxyphenyl)-5′-isobutyl-4′-nitrospiro[indoline-3,2′-pyrrolidin]-2-one.** The crude product was purified by column chromatography over silica gel (100–200 mesh) using ethyl acetate/hexane (20:80) as an eluent; combined yield: (±) 72%; yellow solid; m.p. 182–184 °C; ^1^H NMR (400 MHz, CDCl_3_) δ 7.64 (d, *J* = 7.2 Hz, 1H), 7.28 (s, 1H), 7.20–7.16 (m, 1H), 7.11 (td, *J* = 7.6, 0.8 Hz, 1H), 6.79 (d, *J* = 2.8 Hz, 1H), 6.66–6.63 (m, 2H), 6.55 (d, *J* = 9.2 Hz, 1H), 5.79 (dd, *J* = 6.8, 6.4 Hz, 1H), 5.00 (d, *J* = 6.4 Hz, 1H), 4.69–4.62 (m, 1H), 3.65 (s, 3H), 3.27 (s, 3H), 2.56 (s, 1H), 1.84–1.74 (m, 1H), 1.44–1.33 (m, 2H), 0.96 (dd, *J* = 6.4, 5.2 Hz, 6H); ^13^C NMR (100 MHz, CDCl_3_) δ 178.49, 153.27, 151.75, 140.71, 129.45, 129.38, 124.28, 123.73, 122.85, 114.85, 113.39, 111.71, 109.41, 94.90, 73.05, 59.83, 55.72, 55.69, 52.63, 39.41, 25.60, 23.61, 21.88; HRMS (ESI/QTOF) *m*/*z*: [M + H]^+^ calcd for C_23_H_28_N_3_O_5_ 426.2024; found 426.2017.

**Compound 4b: 3′-(2,5-dimethoxyphenyl)-5′-methyl-4′-nitrospiro[indoline-3,2′-pyrrolidin]-2-one.** The crude product was purified by column chromatography over silica gel (100–200 mesh) using ethyl acetate/hexane (20:80) as an eluent; combined yield: (±) 91%; yellow solid; m.p. 150–152 °C; ^1^H NMR (400 MHz, CDCl_3_) δ 7.63 (d, *J* = 7.6 Hz, 1H), 7.51 (s, 1H), 7.18 (td, *J* = 7.6, 1.2 Hz, 1H), 7.13–7.01 (m, 1H), 6.82 (d, *J* = 2.8 Hz, 1H), 6.66–6.62 (m, 2H), 6.55 (d, *J* = 9.2 Hz, 1H), 5.83–5.80 (m, 1H), 5.08 (d, *J* = 7.2 Hz, 1H), 4.75–4.72 (m, 1H), 3.62 (s, 3H), 3.28 (s, 3H), 2.56 (s, 1H), 1.28 (d, *J* = 6.8 Hz, 3H); ^13^C NMR (100 MHz, CDCl_3_) δ 178.85, 153.29, 151.79, 140.73, 129.43, 129.12, 124.34, 123.39, 122.85, 114.62, 113.57, 111.77, 109.52, 94.63, 73.14, 56.62, 55.74, 55.66, 51.83, 16.08; HRMS (ESI/QTOF) *m*/*z*: [M + H]^+^ calcd for C_20_H_22_N_3_O_5_ 384.1554; found 384.1565.

**Compound 4c: 5-bromo-3′-(2**,**5-dimethoxyphenyl)-5′-isobutyl-4′-nitrospiro[indoline-3**,**2′-pyrrolidin]-2-one.** The crude product was purified by column chromatography over silica gel (100–200 mesh) using ethyl acetate/hexane (22:78) as an eluent; combined yield: (±) 76%; yellow solid; m.p. 142–144 °C; ^1^H NMR (400 MHz, CDCl_3_) δ 7.76 (d, *J* = 1.6 Hz, 1H), 7.31 (dd, *J* = 8.4, 2.0 Hz, 1H), 7.25 (s, 1H), 6.80 (d, *J* = 2.8 Hz, 1H), 6.65 (dd, *J* = 8.8, 2.8 Hz, 1H), 6.57–6.52 (m, 2H), 5.81 (t, *J* = 7.2 Hz, 1H), 5.01 (d, *J* = 6.8 Hz, 1H), 4.67–4.62 (m, 1H), 3.66 (s, 3H), 3.34 (s, 3H), 2.59 (br, 1H), 1.79–1.72 (m, 1H), 1.46–1.32 (m, 2H), 0.96 (dd, *J* = 9.2, 6.8 Hz, 6H); ^13^C NMR (100 MHz, CDCl_3_) δ 178.16, 153.28, 151.65, 139.66, 132.14, 131.37, 127.97, 122.88, 115.35, 114.68, 113.46, 111.43, 110.87, 93.83, 72.79, 59.07, 55.72, 55.58, 51.76, 39.63, 25.46, 23.67, 21.74; HRMS (ESI/QTOF) *m*/*z*: [M + H]^+^ calcd for C_23_H_27_BrN_3_O_5_ 504.1129; found 504.1125.

**Compound 4d: 3′-(2**,**5-dimethoxyphenyl)-5′-isopropyl-4′-nitrospiro[indoline-3**,**2′-pyrrolidin]-2-one.** The crude product was purified by column chromatography over silica gel (100–200 mesh) using ethyl acetate/hexane (22:78) as an eluent; combined yield: (±) 78%; off white solid; m.p. 210–212 °C; ^1^H NMR (400 MHz, Chloroform-d) δ 7.74 (s, 1H), 7.65 (d, *J* = 7.6 Hz, 1H), 7.19–7.14 (m, 2H), 6.73 (d, *J* = 2.8 Hz, 1H), 6.66–6.57 (m, 2H), 6.51 (d, *J* = 8.8 Hz, 1H), 5.78–5.75 (m, 1H), 4.88 (d, *J* = 4.8 Hz, 1H), 4.23–4.27 (m, 1H), 3.60 (s, 3H), 3.20 (s, 3H), 2.71 (s, 1H), 1.70–1.62 (m, 1H), 1.07 (dd, *J* = 6.4, 4.8 Hz, 6H); ^13^C NMR (100 MHz, Chloroform-d) δ 178.85, 153.16, 151.55, 140.86, 130.12, 129.26, 124.41, 124.15, 122.88, 115.25, 113.02, 111.46, 109.48, 94.53, 72.91, 69.90, 55.65, 55.58, 54.42, 29.66, 20.82, 20.79; HRMS (ESI/QTOF) *m*/*z*: [M + H]^+^ calcd for C_22_H_25_N_3_O_5_ 412.1867; found 412.1832.

**Compound 4e: 5-bromo-3′-(2**,**5-dimethoxyphenyl)-5′-methyl-4′-nitrospiro[indoline-3**,**2′-pyrrolidin]-2-one.** The crude product was purified by column chromatography over silica gel (100–200 mesh) using ethyl acetate/hexane (30:70) as an eluent; combined yield: (±) 85%; white solid; m.p. 108–110 °C; ^1^H NMR (400 MHz, CDCl_3_) δ 7.76 (d, *J* = 1.6 Hz, 1H), 7.31 (dd, *J* = 8.4, 2.0 Hz, 1H), 7.22 (s, 1H), 6.81 (t, *J* = 3.2 Hz, 1H), 6.65 (dd, *J* = 8.8, 3.2 Hz, 1H), 6.57–6.52 (m, 2H), 5.84 (t, *J* = 7.68 Hz, 1H), 5.08 (d, *J* = 7.6 Hz, 1H), 4.74–4.70 (m, 1H), 3.67 (s, 3H), 3.35 (s, 3H), 2.54 (s, 1H), 1.28 (d, *J* = 6.4 Hz, 3H); ^13^C NMR (100 MHz, CDCl_3_) δ 178.30, 153.31, 151.71, 139.59, 132.18, 131.06, 128.10, 122.53, 115.38, 114.45, 113.64, 111.49, 110.90, 93.38, 72.80, 55.93, 55.72, 55.60, 50.94, 16.51; HRMS (ESI/QTOF) *m*/*z*: [M + H]^+^ calcd for C_20_H_21_BrN_3_O_5_ 462.0659; found 462.0677.

**Compound 4f: 3′-(3-(benzyloxy)-4-methoxyphenyl)-5-bromo-5′-isobutyl-4′-nitrospiro[indoline-3**,**2′-pyrrolidin]-2-one.** The crude product was purified by column chromatography over silica gel (100–200 mesh) using ethyl acetate/hexane (25:75) as an eluent; combined yield: (±) 79%; solid; m.p. 95–97 °C; ^1^H NMR (400 MHz, CDCl_3_) δ 7.64 (s, 1H), 7.36–7.29 (m, 6H), 7.13 (s, 1H), 6.65 (d, *J* = 8.4 Hz, 1H), 6.58 (t, *J* = 7.2 Hz, 1H), 6.49–6.50 (m, 2H), 5.83–5.79 (m, 1H), 4.89 (s, 2H), 4.63–4.57 (m, 1H), 4.22 (d, *J* = 8.4 Hz, 1H), 3.76 (s, 3H), 2.38 (s, 1H), 1.76–1.67 (m, 1H), 1.45–1.38 (m, 1H), 1.30–1.24 (m, 1H), 0.94 (dd, *J* = 13.2, 6.4 Hz, 6H); ^13^C NMR (100 MHz, CDCl_3_) δ 178.62, 149.53, 147.71, 139.90, 136.90, 132.66, 131.09, 128.64, 127.91, 127.30, 127.05, 124.68, 120.85, 115.80, 113.28, 111.50, 111.37, 92.00, 72.61, 70.70, 58.31, 57.03, 50.95, 40.22, 25.14, 23.83, 21.46; HRMS (ESI/QTOF) *m*/*z*: [M + H]^+^ calcd for C_29_H_31_BrN_3_O_5_ 580.1442; found 580.1433.

**Compound 4g: 5′-benzyl-3′-(3-(benzyloxy)-4-methoxyphenyl)-5-bromo-4′-nitrospiro[indoline-3**,**2′-pyrrolidin]-2-one.** The crude product was purified by column chromatography over silica gel (100–200 mesh) using ethyl acetate/hexane (20:80) as an eluent. combined yield: (±) 70%; yellow solid; m.p. 155–157 °C; ^1^H NMR (400 MHz, Chloroform-d) δ 7.69 (d, *J* = 2.0 Hz, 1H), 7.37–7.29 (m, 11H), 6.72–6.67 (m, 3H), 6.50 (d, *J* = 2.0 Hz, 1H), 6.43 (d, *J* = 8.4 Hz, 1H), 6.02–5.98 (m, 1H), 4.92 (d, *J* = 3.6 Hz, 2H), 4.85–4.79 (m, 1H), 3.80 (s, 3H), 2.89 (dd, *J* = 13.2, 3.6 Hz, 1H), 2.67 (dd, *J* = 13.1, 10.8 Hz, 1H); ^13^C NMR (100 MHz, Chloroform-d) δ 178.82, 149.59, 147.60, 139.64, 137.25, 136.85, 132.52, 130.71, 129.34, 128.69, 128.55, 127.25, 126.81, 123.94, 120.89, 115.78, 113.04, 111.44, 111.20, 89.86, 71.73, 70.48, 58.11, 57.19, 55.80, 38.32;HRMS (ESI/QTOF) *m*/*z*: [M + H]^+^ calcd for C_32_H_28_BrN_3_O_5_ 614.1285; found 614.1265.

**Compound 4h: 3′-(3**,**4-dimethoxyphenyl)-5′-isobutyl-4′-nitrospiro[indoline-3**,**2′-pyrrolidin]-2-one.** The crude product was purified by column chromatography over silica gel (100–200 mesh) using ethyl acetate/hexane (20:80) as an eluent. combined yield: (±) 82%; yellow solid; m.p. 73–75 °C; ^1^H NMR (400 MHz,) δ 7.52 (d, *J* = 7.4 Hz, 1H), 7.11–7.07 (m, 1H), 6.96 (s, 1H), 6.59–6.53 (m, 3H), 6.24 (d, *J* = 2.0 Hz, 1H), 5.93–5.88 (m, 1H), 4.63–4.58 (m, 1H), 4.29 (d, *J* = 8.4 Hz, 1H), 3.82 (d, *J* = 18.0 Hz, 1H), 3.70 (s, 3H), 3.49 (s, 3H), 2.39 (br, 1H), 1.43–1.35 (m, 1H), 0.90 (dd, *J* = 10.4, 6.4 Hz, 6H), 0.80–0.74 (m, 2H); ^13^C NMR (100 MHz, Chloroform-d) δ 179.65, 148.62, 148.45, 141.33, 129.83, 128.99, 125.23, 123.68, 123.21, 119.48, 110.95, 110.90, 110.14, 92.02, 72.92, 60.54, 58.25, 57.09, 55.65, 55.48, 40.26, 25.17, 23.85, 21.51; HRMS (ESI/QTOF) *m*/*z*: [M + H]^+^ calcd for C_23_H_27_N_3_O_5_ 426.2024; found 426.2001.

**Compound 4i: 3′-(3**,**4-dimethoxyphenyl)-5′-methyl-4′-nitrospiro[indoline-3**,**2′-pyrrolidin]-2-one.** The crude product was purified by column chromatography over silica gel (100–200 mesh) using ethyl acetate/hexane (30:70) as an eluent; combined yield: (±) 86%; white solid; m.p. 165–167 °C; ^1^H NMR (400 MHz, CDCl_3_) δ = 7.58 (d, *J* = 7.2 Hz, 1H), 7.17–7.12 (m, 2H), 6.65–6.62 (m, 3H), 6.30 (s, 1H), 5.98 (t, *J* = 8.4 Hz, 1H), 4.76–4.73 (m, 1H), 4.38 (d, *J* = 8.8 Hz, 1H), 4.13–4.08 (m, 1H), 3.75 (s, 3H), 3.54 (s, 3H), 2.47 (br, 1H), 1.27 (d, *J* = 6.8 Hz, 3H); ^13^C NMR (100 MHz, CDCl_3_) δ 178.93, 148.72, 148.50, 140.94, 129.88, 128.75, 124.97, 123.75, 123.32, 119.56, 110.90, 109.94, 91.70, 72.89, 60.51, 57.93, 55.75, 55.56, 54.52, 17.18; HRMS (ESI/QTOF) *m*/*z*: [M + H]^+^ calcd for C_20_H_22_N_3_O_5_ 384.1554; found 384.1567.

**Compound 4j: 5-bromo-3′-(3**,**4-dimethoxyphenyl)-5′-methyl-4′-nitrospiro[indoline-3**,**2′-pyrrolidin]-2-one.** The crude product was purified by column chromatography over silica gel (100–200 mesh) using ethyl acetate/hexane (35:65) as an eluent; combined yield: (±) 89%; solid; m.p. 155–157 °C; ^1^H NMR (400 MHz, CDCl_3_) δ 7.71–7.69 (m, 2H), 7.35 (d, *J* = 8.0 Hz, 1H), 6.63–6.53 (m, 3H), 6.36 (s, 1H), 5.94 (t, *J* = 8.8 Hz, 1H), 4.73–4.69 (m, 1H), 4.35 (d, *J* = 8.8 Hz, 1H), 3.73 (s, 3H), 3.58 (s, 3H), 2.31 (br, 1H), 1.26 (d, *J* = 6.4 Hz, 3H); ^13^C NMR (100 MHz, CDCl_3_) δ 179.07, 148.88, 148.60, 140.03, 132.72, 130.98, 127.08, 124.53, 119.86, 115.84, 111.60, 111.00, 110.84, 91.29, 72.87, 57.76, 55.76, 55.66, 54.37, 17.31; HRMS (ESI/QTOF) *m*/*z*: [M + H]^+^ calcd for C_20_H_21_BrN_3_O_5_ 462.0659 found 464.0626.

**Compound 4k: 3′-(4-(benzyloxy)-3-methoxyphenyl)-5′-isobutyl-4′-nitrospiro[indoline-3**,**2′-pyrrolidin]-2-one.** The crude product was purified by column chromatography over silica gel (100–200 mesh) using ethyl acetate/hexane (30:70) as an eluent; combined yield: (±) 86%; white solid; m.p. 68–70 °C; ^1^H NMR (400 MHz, CDCl_3_) δ 7.57 (d, *J* = 7.2 Hz, 1H), 7.35–7.26 (m, 6H), 7.15–7.13 (m, 1H), 6.65–6.62 (m, 2H), 6.55–6.52 (m, 1H), 6.31 (d, *J* = 1.6 Hz, 1H), 5.96–5.92 (m, 1H), 4.99 (s, 2H), 4.68–4.63 (m, 1H), 4.35–4.33 (m, 1H), 3.54 (s, 3H), 3.46 (d, *J* = 3.2 Hz, 2H), 2.42 (br, 1H), 1.48–1.41 (m, 1H), 1.34–1.28 (m, 1H), 0.97–0.92 (m, 6H); ^13^C NMR (100 MHz, CDCl_3_) δ 178.96, 149.09, 147.91, 141.04, 136.88, 129.85, 128.94, 128.61, 127.98, 127.37, 125.70, 123.73, 123.28, 119.48, 113.41, 111.42, 109.97, 92.14, 72.84, 70.84, 58.43, 57.26, 55.66, 40.21, 25.22, 23.85, 21.52; HRMS (ESI/QTOF) *m*/*z*: [M + H]^+^ calcd for C_29_H_32_N_3_O_5_ 502.2337; found 502.2331.

**Compound 4l: 3′-(4-(benzyloxy)-3-methoxyphenyl)-5′-methyl-4′-nitrospiro[indoline-3**,**2′-pyrrolidin]-2-one.** The crude product was purified by column chromatography over silica gel (100–200 mesh) using ethyl acetate/hexane (30:70) as an eluent; combined yield: (±) 82%; white solid; m.p. 168–170 °C; ^1^H NMR (400 MHz, CDCl_3_) δ 7.58 (d, *J* = 7.2 Hz, 1H), 7.36–7.27 (m, 5H), 7.23–7.21 (m, 1H), 7.17–7.13 (m, 1H), 6.94 (s, 1H), 6.65 (t, *J* = 8.4 Hz, 2H), 6.55–6.53 (m, 1H), 6.32 (d, *J* = 2.0 Hz, 1H), 5.96 (t, *J* = 8.8 Hz, 1H), 5.02 (s, 2H), 4.78–4.71 (m, 1H), 4.37 (d, *J* = 8.8 Hz, 1H), 3.56 (s, 3H), 2.46 (br, 1H), 1.27 (d, *J* = 6.8 Hz, 3H); ^13^C NMR (100 MHz, CDCl_3_) δ 178.73, 149.16, 147.99, 140.89, 136.92, 129.87, 128.74, 128.61, 127.97, 127.35, 125.52, 123.74, 123.32, 119.54, 113.51, 111.47, 109.89, 91.78, 72.86, 70.89, 58.01, 55.68, 54.57, 17.13; HRMS (ESI/QTOF) *m*/*z*: [M + H]^+^ calcd for C_26_H_26_N_3_O_5_ 460.1867; found 460.1894.

**Compound 4m: 3′-(4-(benzyloxy)-3-methoxyphenyl)-5-bromo-5′-methyl-4′-nitrospiro[indoline-3**,**2′-pyrrolidin]-2-one.** The crude product was purified by column chromatography over silica gel (100–200 mesh) using ethyl acetate/hexane (22:78) as an eluent; combined yield: (±) 82%; solid; m.p. 168–170 °C; ^1^H NMR (400 MHz, CDCl_3_) δ 7.69 (d, *J* = 1.6 Hz, 1H), 7.36–7.28 (m, 6H), 7.19 (s, 1H), 6.66 (d, *J* = 8.4 Hz, 1H), 6.54–6.51 (m, 2H), 6.39 (d, *J* = 2.0 Hz, 1H), 5.93 (t, *J* = 8.8 Hz, 1H), 5.01 (s, 2H), 4.74–4.70 (m, 1H), 4.34 (t, *J* = 8.8 Hz, 1H), 3.61 (s, 3H), 2.59 (br, 1H), 1.26 (d, *J* = 6.4 Hz, 3H); ^13^C NMR (100 MHz, CDCl_3_) δ 178.52, 149.11, 148.02, 139.74, 136.69, 132.58, 130.86, 128.52, 127.90, 127.25, 126.99, 124.89, 119.69, 115.73, 113.39, 111.33, 111.24, 91.21, 72.68, 70.76, 57.74, 55.67, 54.26, 17.19; HRMS (ESI/QTOF) *m*/*z*: [M + H]^+^ calcd for C_26_H_25_BrN_3_O_5_ 538.0972; found 538.0953.

**Compound 4n: 2′-(4-(benzyloxy)-3-methoxyphenyl)-1′-nitro-1′**,**2′**,**5′**,**6′**,**7′**,**7a′-hexahydrospiro[indoline-3**,**3′-pyrrolizin]-2-one.** The crude product was purified by column chromatography over silica gel (100–200 mesh) using ethyl acetate/hexane (18:82) as an eluent; combined yield: (±) 92%; yellow solid; m.p. 66–68 °C; ^1^H NMR (400 MHz, CDCl_3_) δ 7.49 (d, *J* = 7.2 Hz, 1H), 7.34–7.31 (m, 5H), 7.23–7.19 (m, 1H), 7.09–7.04 (m, 2H), 6.68–6.58 (m, 4H), 6.15 (dd, *J* = 10.4, 9.2 Hz, 1H), 4.91–4.81 (m, 3H), 4.36 (d, *J* = 10.4 Hz, 1H), 3.74 (s, 3H), 3.24–3.18 (m, 1H), 2.87–2.83 (m, 1H), 2.15–2.08 (m, 1H), 1.98–1.94 (m, 1H), 1.83–1.79 (m, 1H), 1.49–1.44 (m, 1H); ^13^C NMR (100 MHz, CDCl_3_) δ 177.73, 149.26, 147.68, 141.86, 137.03, 130.00, 128.56, 127.79, 127.45, 126.19, 125.52, 124.74, 122.47, 121.25, 113.41, 111.56, 110.23, 91.76, 75.00, 70.52, 64.19, 55.84, 52.99, 51.15, 27.90, 25.75; HRMS (ESI/QTOF) *m*/*z*: [M + H]^+^ calcd for C_28_H_28_N_3_O_5_ 486.2024; found 486.2015.

**Compound 4o: 2′-(4-(benzyloxy)-3-methoxyphenyl)-5-bromo-1′-nitro-1′**,**2′**,**5′**,**6′**,**7′**,**7a′-hexahydrospiro[indoline-3**,**3′-pyrrolizin]-2-one.** The crude product was purified by column chromatography over silica gel (100–200 mesh) using ethyl acetate/hexane (20:80) as an eluent; combined yield: (±) 83%; solid; m.p. 95–97 °C; ^1^H NMR (400 MHz, CDCl_3_) δ 7.63 (d, *J* = 2.0 Hz, 1H), 7.36–7.31 (m, 6H), 7.20 (s, 1H), 6.65 (s, 2H), 6.60 (s, 1H), 6.50 (d, *J* = 8.4 Hz, 1H), 6.12–6.07 (m, 1H), 4.92–4.77 (m, 3H), 4.32 (d, *J* = 10.4 Hz, 1H), 3.78 (s, 3H), 3.17–3.14 (m, 1H), 2.86 (t, *J* = 7.2 Hz, 1H), 2.13–2.08 (m, 1H), 2.03–1.97 (m, 1H), 1.84–1.79 (m, 1H), 1.48–1.45 (m, 1H); ^13^C NMR (100 MHz, CDCl_3_) δ 177.30, 149.41, 147.70, 140.89, 136.96, 132.91, 129.18, 128.59, 127.81, 127.70, 127.40, 124.23, 121.34, 114.98, 113.18, 111.72, 111.67, 91.51, 75.01, 70.44, 64.29, 55.87, 53.28, 51.14, 27.82, 25.75; HRMS (ESI/QTOF) *m*/*z*: [M + H]^+^ calcd for C_28_H_27_BrN_3_O_5_ 564.1129; found 566.1119.

**Compound 4p: 2′-(3-(benzyloxy)-4-methoxyphenyl)-1′-nitro-1′**,**2′**,**5′**,**6′**,**7′**,**7a′-hexahydrospiro[indoline-3**,**3′-pyrrolizin]-2-one.** The crude product was purified by column chromatography over silica gel (100–200 mesh) using ethyl acetate/hexane (20:80) as an eluent; combined yield: (±) 79%; white solid; m.p. 83–85 °C; ^1^H NMR (400 MHz, CDCl_3_) δ 7.56 (d, *J* = 7.6 Hz, 1H), 7.33–7.25 (m, 6H), 7.24–7.21 (m, 1H), 7.12–7.07 (m, 1H), 6.68–6.62 (m, 3H), 6.48 (d, *J* = 1.2 Hz, 1H), 6.26–6.21 (m, 1H), 4.99 (s, 2H), 4.84 (dd, *J* = 16.8, 8.0 Hz, 1H), 4.44 (d, *J* = 10.4 Hz, 1H), 3.58 (s, 3H), 3.27–3.24 (m, 1H), 2.89–2.86 (m, 1H), 2.17–2.10 (m, 1H), 2.00–1.96 (m, 1H), 1.87–1.79 (m, 1H), 1.52–1.46 (m, 1H); ^13^C NMR (100 MHz, CDCl_3_) δ 177.65, 149.16, 147.84, 141.90, 136.94, 130.11, 128.61, 127.95, 127.33, 126.30, 125.62, 125.27, 122.47, 120.08, 113.52, 111.67, 110.34, 91.51, 75.19, 70.85, 64.26, 55.72, 53.09, 51.18, 27.90, 25.76; HRMS (ESI/QTOF) *m*/*z*: [M + H]^+^ calcd for C_28_H_28_N_3_O_5_ 486.2024; found 486.2076.

**Compound 4q: 2′-(3-(benzyloxy)-4-methoxyphenyl)-5-bromo-1′-nitro-1′**,**2′**,**5′**,**6′**,**7′**,**7a′-hexahydrospiro[indoline-3**,**3′-pyrrolizin]-2-one.** The crude product was purified by column chromatography over silica gel (100–200 mesh) using ethyl acetate/hexane (20:80) as an eluent; combined yield: (±) 88%; solid; m.p. 98–100 °C; ^1^H NMR (400 MHz, CDCl_3_) δ 7.69 (d, *J* = 2.0 Hz, 1H), 7.45 (s, 1H), 7.38–7.28 (m, 6H), 6.67–6.62 (m, 2H), 6.57 (d, *J* = 8.4 Hz, 1H), 6.51 (d, *J* = 2.0 Hz, 1H), 6.22–6.17 (m, 1H), 5.00 (s, 2H), 4.85–4.79 (m, 1H), 4.39 (d, *J* = 10.4 Hz, 1H), 3.64 (s, 3H), 3.22–3.16 (m, 1H), 2.89 (t, *J* = 7.2 Hz, 1H), 2.17–2.10 (m, 1H), 2.03–1.99 (m, 1H), 1.86–1.82 (m, 1H), 1.51–1.48 (m, 1H); ^13^C NMR (100 MHz, CDCl_3_) δ 177.29, 149.27, 147.99, 140.93, 136.86, 132.96, 129.28, 128.63, 128.00, 127.76, 127.34, 124.76, 120.09, 115.00, 113.60, 111.83, 111.63, 91.29, 75.21, 70.86, 64.33, 55.75, 53.34, 51.17, 27.83, 25.76; HRMS (ESI/QTOF) *m*/*z*: [M + H]^+^ calcd for C_28_H_27_BrN_3_O_5_ 564.1129; found 564.1123.

**Compound 4r: 2′-(4-methoxyphenyl)-1′-nitro-1′**,**2′**,**5′**,**6′**,**7′**,**7a′-hexahydrospiro[indoline-3**,**3′-pyrrolizin]-2-one.** The crude product was purified by column chromatography over silica gel (100–200 mesh) using ethyl acetate/hexane (18:82) as an eluent; combined yield: (±) 77%; white solid; m.p. 116–118 °C; ^1^H NMR (400 MHz, CDCl_3_) δ 7.56 (d, *J* = 7.2 Hz, 1H), 7.44 (s, 1H), 7.26–7.21 (m, 1H), 7.10 (td, *J* = 7.6, 0.8 Hz, 1H), 7.02 (t, *J* = 8.0 Hz, 1H), 6.71–6.64 (m, 3H), 6.58–6.56 (m, 1H), 6.30–6.25 (m, 1H), 4.88–4.82 (m, 1H), 4.48 (d, *J* = 10.4 Hz, 1H), 3.56 (s, 3H), 3.28–3.22 (m, 1H), 2.87 (t, *J* = 7.2 Hz, 1H), 2.18–2.10 (m, 1H), 2.00 –1.96 (m, 1H), 1.85–1.77 (m, 1H), 1.54–1.46 (m, 1H); ^13^C NMR (100 MHz, CDCl_3_) δ 177.60, 159.48, 141.85, 134.11, 130.15, 129.63, 126.29, 125.40, 122.53, 120.43, 113.85, 113.68, 110.32, 91.72, 75.13, 64.23, 55.07, 53.23, 51.18, 27.90, 25.73; HRMS (ESI/QTOF) *m*/*z*: [M + H]^+^ calcd for C_21_H_22_N_3_O_4_ 380.1605; found 380.1601.

**Compound 4s: 2′-(2**,**5-dimethoxyphenyl)-1′-nitro-1′**,**2′**,**5′**,**6′**,**7′**,**7a′-hexahydrospiro[indoline-3**,**3′-pyrrolizin]-2-one.** The crude product was purified by column chromatography over silica gel (100–200 mesh) using ethyl acetate/hexane (18:82) as an eluent; combined yield: (±) 78%; solid; m.p. 174–176 °C; ^1^H NMR (400 MHz, DMSO-d_6_) δ 10.32 (s, 1H), 7.56 (d, *J* = 7.6 Hz, 1H), 7.13 (td, *J* = 7.6, 0.8 Hz, 1H), 6.96–6.91 (m, 2H), 6.66–6.57 (m, 3H), 6.19–6.14 (m, 1H), 5.13 (d, *J* = 11.2 Hz, 1H), 4.60 (q, *J* = 8.0 Hz, 1H), 3.57 (s, 3H), 3.42 (s, 3H), 3.23–3.17 (m, 1H), 2.58 (t, *J* = 7.2 Hz, 1H), 2.02–1.85 (m, 2H), 1.64–1.57 (m, 1H), 1.31–1.25 (m, 1H); ^13^C NMR (100MHz, DMSO-d_6_) δ 177.86, 153.23, 152.02, 143.31, 130.17, 128.02, 124.90, 122.97, 121.43, 114.56, 113.22, 112.74, 109.90, 92.73, 74.92, 63.81, 56.29, 55.61, 51.24, 43.28, 28.07, 25.58; HRMS (ESI/QTOF) *m*/*z*: [M + H]^+^ calcd for C_22_H_24_N_3_O_5_ 410.1711; found 410.1710.

**Compound 4t: 5-bromo-2′-(3-methoxyphenyl)-1′-nitro-1′**,**2′**,**5′**,**6′**,**7′**,**7a′-hexahydrospiro[indoline-3**,**3′-pyrrolizin]-2-one.** The crude product was purified by column chromatography over silica gel (100–200 mesh) using ethyl acetate/hexane (20:80) as an eluent; combined yield: (±) 80%; solid; m.p. 145–147 °C; ^1^H NMR (400 MHz, DMSO-d_6_) δ 10.39 (s, 1H), 7.34 (dd, *J* = 8.0, 2.0 Hz, 1H), 7.08–7.04 (m, 1H), 6.73–6.66 (m, 3H), 6.55 (d, *J* = 8.4 Hz, 1H), 6.33–6.28 (m, 1H), 4.58–4.52 (m, 2H), 3.54 (s, 3H), 2.57–2.54 (m, 1H), 1.94–1.82 (m, 3H), 1.64–1.29 (m, 3H); ^13^C NMR (100 MHz, DMSO-d_6_) δ 177.44, 159.40, 142.88, 135.27, 133.16, 130.63, 130.05, 127.79, 120.57, 114.66, 113.86, 113.49, 112.06, 90.76, 75.00, 63.63, 55.26, 51.10, 51.05, 28.25, 25.66; HRMS (ESI/QTOF) *m*/*z*: [M + H]^+^ calcd for C_21_H_20_BrN_3_O_4_ 458.0710; found 458.0714.

**Compound 4u: 5-bromo-2′-(2**,**5-dimethoxyphenyl)-1′-nitro-1′**,**2′**,**5′**,**6′**,**7′**,**7a′-hexahydrospiro[indoline-3**,**3′-pyrrolizin]-2-one.** The crude product was purified by column chromatography over silica gel (100–200 mesh) using ethyl acetate/hexane (20:80) as an eluent; combined yield: (±) 80%; solid; m.p. 190–192 °C; ^1^H NMR (400 MHz, CDCl_3_) δ 7.74 (s, 2H), 7.30 (dd, *J* = 8.0, 1.6 Hz, 1H), 6.97 (d, *J* = 2.8 Hz, 1H), 6.63–6.53 (m, 3H), 6.18–6.14 (m, 1H), 5.30 (d, *J* = 10.8 Hz, 1H), 4.81 (q, *J* = 8.0 Hz, 1H), 3.59 (s, 3H), 3.58 (s, 3H), 3.29–3.23 (m, 1H), 2.86 (t, *J* = 7.2 Hz, 1H), 2.15–2.09 (m, 1H), 2.03–1.98 (m, 1H), 1.84–1.78 (m, 1H), 1.55–1.47 (m, 1H); ^13^C NMR (100 MHz, CDCl_3_) δ 177.63, 153.39, 151.73, 140.72, 132.54, 131.01, 126.91, 121.61, 114.30, 113.87, 113.79, 111.67, 111.18, 92.15, 75.09, 64.28, 55.84, 55.57, 51.18, 43.90, 27.89, 25.69; HRMS (ESI/QTOF) *m*/*z*: [M + H]^+^ calcd for C_22_H_23_BrN_3_O_5_ 488.0816; found 488.0807.

**Compound 4v: 2′-(3**,**4-dimethoxyphenyl)-1′-nitro-1′**,**2′**,**5′**,**6′**,**7′**,**7a′-hexahydrospiro[indoline-3**,**3′-pyrrolizin]-2-one.** The crude product was purified by column chromatography over silica gel (100–200 mesh) using ethyl acetate/hexane (18:82) as an eluent; combined yield: (±) 72%; white solid; m.p. 175–177 °C; ^1^H NMR (400 MHz, CDCl_3_) δ 7.57 (d, *J* = 7.2 Hz, 2H), 7.56–7.22 (m, 1H), 7.12–7.08 (m, 1H), 6.70–6.67 (m, 2H), 6.61 (d, *J* = 8.4 Hz, 1H), 6.45 (d, *J* = 2.0 Hz, 1H), 6.25 (t, *J* = 10.4 Hz, 1H), 4.84 (q, 8.0 Hz, 1H), 4.44 (d, *J* = 10.4 Hz, 1H), 3.74 (s, 3H), 3.56 (s, 3H), 3.29–3.23 (m, 1H), 2.88 (t, *J* = 7.2 Hz, 1H), 2.17–2.10 (m, 1H), 2.02–1.96 (m, 1H), 1.86–1.78 (m, 1H), 1.55–1.45 (m, 1H); ^13^C NMR (100 MHz, CDCl_3_) δ 177.97, 148.58, 148.55, 142.03, 130.12, 126.28, 125.63, 124.76, 122.44, 120.10, 111.16, 111.01, 110.43, 91.45, 75.23, 64.24, 55.73, 55.59, 53.04, 51.21, 27.93, 25.77; HRMS (ESI/QTOF) *m*/*z*: [M + H]^+^ calcd for C_22_H_24_N_3_O_5_ 410.1711; found 410.1724.

**Compound 4w: 5-bromo-2′-(3**,**4-dimethoxyphenyl)-1′-nitro-1′**,**2′**,**5′**,**6′**,**7′**,**7a′-hexahydrospiro [indoline-3**,**3′-pyrrolizin]-2-one.** The crude product was purified by column chromatography over silica gel (100–200 mesh) using ethyl acetate/hexane (20:80) as an eluent; combined yield: (±) 77%; solid; m.p. 195–197 °C; ^1^H NMR (400 MHz, CDCl_3_) δ 7.77 (s, 1H), 7.69 (s, 1H), 7.37 (d, *J* = 7.6 Hz, 1H), 6.87–6.57 (m, 3H), 6.49 (s, 1H), 6.20 (t, *J* = 10.0 Hz, 1H), 4.82–4.78 (m, 1H), 4.39 (d, *J* = 10.4 Hz, 1H), 3.75 (s, 3H), 3.61 (s, 3H), 3.22–3.17 (m, 1H), 2.89 (t, *J* = 6.5 Hz, 1H), 2.17–2.10 (m, 1H), 2.02–2.00 (m, 1H), 1.85–1.79 (m, 1H), 1.53–1.43 (m, 1H); ^13^C NMR (100 MHz, CDCl_3_) δ 177.55, 148.74, 148.68, 141.03, 132.97, 129.29, 127.77, 124.28, 120.15, 114.99, 111.88, 111.15, 91.22, 75.24, 64.31, 55.77, 55.64, 53.29, 51.20, 27.85, 25.76; HRMS (ESI/QTOF) *m*/*z*: [M + H]^+^ calcd for C_22_H_23_BrN_3_O_5_ 488.0816; found 488.0810.

### 3.5. Cell Culture, Reagents and Cells

Adenocarcinomic human alveolar basal epithelial (A549) and human hepatoma (HepG2) cancer cell lines, immortalized normal human liver (LO2), human bronchial epithelial (BEAS-2B cells were purchased from ATCC (ATCC, Manassas, VA, USA). The cells were cultured in RPMI-1640 (A549 cell lines) and DMEM (HepG2, LO2 and BEAS-2B) medium supplemented with 10% fetal bovine serum and antibiotics: penicillin (50 U/Ml) and streptomycin (50 μg/mL; Invitrogen, Waltham, MA, USA). All cells were incubated at 37 °C in a 5% humidified CO_2_ incubator). All test compounds were dissolved in DMSO at a final concentration of 50 mM and stored at −20 °C before use.

### 3.6. Cytotoxicity Assay

Cytotoxicity was assessed by the MTT (5 mg/mL) assay, as described previously [87,88,89,90]. Briefly, 5 × 10^3^ cells per well were seeded in 96-well plates before drug treatments. After overnight cell culture, the cells were exposed to different concentrations of selected compounds (0.19–100 μM) for 72 h. Cells without drug treatment were used as controls. Subsequently, 10 μL of 5 mg/mL MTT solution was added to each well and incubated at 37 °C for 4 h, followed by the addition of 100 μL solubilization buffer (12 mM HCl in a solution of 10% SDS) and overnight incubation. The absorbance, A570 nm, was then determined in each well on the next day. The percentage of cell viability was calculated using the expression: % viability = A_treated_/A_control_ × 100, and was given as cytotoxicity (Table 2 and Table 3; Figures S1–S4).

## 4. Conclusions

In conclusion, we report a novel series of nitrostyrene-based microwave-assisted functionalized spiro[pyrrolidine-2,3′-oxindoles] **4a**–**w** via the reaction of substituted isatins **1a**–**b**, several *α*-amino acids **2a**–**e** and (*E*)-2-aryl-1-nitroethenes **3a**–**e** in a chemo-/regio-selective manner using [3+2] cycloaddition (Huisgen) reaction under microwave irradiation conditions. The structure elucidation of all the synthesized spirooxindoles was done using ^1^H and ^13^C NMR and HRMS spectral analysis. The stereochemical validation of the structure of compound **4l** was done by single crystal X-ray crystallography study. These 23 derivatives were then assessed for their in vitro anticancer activity against human lung (A549) and liver (HepG2) cancer cell lines along with immortalized normal lung (BEAS-2B) and liver (LO2) cell lines. Out of the synthesized spiro[pyrrolidine-2,3′-oxindoles]; five compounds (**4c**, **4f**, **4m**, **4q**, **4t**) (IC_50_ = 34.99–47.92 µM; SI = 0.96–2.43) displayed significant in vitro anticancer activity against human lung (A549) cancer cell lines, and six compounds (**4c**, **4f**, **4k**, **4m**, **4q**, **4t**) (IC_50_ = 41.56–86.53 µM; SI = 0.49–0.99) displayed encouraging in vitro anticancer activity against human liver (HepG2) cancer cell lines. In the case of lung (A549) cancer cell lines, these compounds were found to be more efficient and selective than standard reference artemisinin (IC_50_ = 100 µM) and chloroquine (IC_50_ = 100 µM; SI: 0.03). Succinctly, our findings qualify the studied molecules as promising anticancer agents that pave the way for further advanced structure–activity relationship studies and therapeutic applications. These synthesized spirooxindole analogs can further be utilized for various other biological activities after performing molecular docking and simulation studies on particular disease target(s).

## Figures and Tables

**Table 1:** Optimization Study ^a^: Synthesis of (3R,3′S,4′S)-3′-(2,5-dimethoxyphenyl)-5′-methyl-4′-nitrospiro[indoline-3,2′-pyrrolidin]-2-one **4b** from isatin **1a**, alanine **2b** and (E)-1,4-dimethoxy-2-(2-nitrovinyl)benzene **3a** as starting materials.

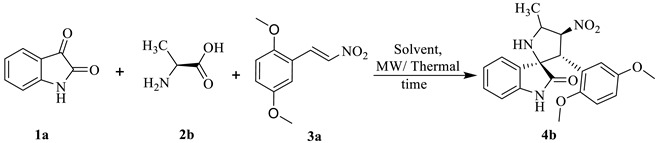						
S.No.	Solvent	Temp. (°C)	Method A ^b^		Method B ^c^	
			Time (min)	Yield (%) ^d^	Time (min)	Yield (%) ^d^
1.	H_2_O	rt	240	NR	30	Trace
2.	H_2_O	90	180	trace	20	NR
3. ^e^	H_2_O	100/120	120	trace	10	NR
4.	MeOH:H_2_O	60	240	55	15	66
5	MeOH:H_2_O	90	180	57	10	62
6. ^f^	MeOH:H_2_O	100/120	120	44	05	48
7.	**MeOH**	**60**	**240**	**65**	**15**	**91**
8.	MeOH	60	180	56	10	82
9.	MeOH	60	120	50	05	61
10.	EtOH:H_2_O	60	240	63	15	59
11.	EtOH:H_2_O	90	180	54	10	49
12. ^g^	EtOH:H_2_O	100/120	120	40	05	36
13.	MeOH	90	240	66	15	90
14. ^h^	MeOH	100/120	240	64	15	89

^a^ Reaction Conditions: **1a** (0.1 mmol), **2b** (0.1 mmol), **3b** (0.1 mmol) in solvent (1 mL), 5–240 min. ^b^ **Method A**: conventional heating; ^c^ **Method B**: microwave irradiation; ^d^ Isolated yield after column chromatography. ^e^ Temperature was set at 100 **°**C under conventional heating and 120 **°**C under microwave heating. ^f–h^ Temperature was set at 100 **°**C under conventional heating and 120 **°**C under microwave heating.

**Table 2:** In vitro cytotoxicity of substituted spiro[pyrrolidine-2,3′-oxindoles] **4a**–**w** against human lung A549 cancer cell lines and the immortalized lung BEAS-2B normal cell lines.

Entry	Trioxane	A549 Cells IC_50_ [µM] ^a^	BEAS-2B Cells IC_50_ [µM] ^a^	S.I. ^b^
1.	**4a**	>100	>100	—
2.	**4b**	>100	>100	—
3.	**4c**	34.99 (±1.77)	33.49 (±3.15)	**0.96**
4.	**4d**	>100	>100	—
5.	**4e**	85.63 (±5.97)	>100	**1.17**
6.	**4f**	41.12 (±7.77)	>100	**2.43**
7.	**4g**	>100	>100	—
8.	**4h**	>100	>100	—
9.	**4i**	>100	>100	—
10.	**4j**	>100	>100	—
11.	**4k**	>100	>100	—
12.	**4l**	>100	>100	—
13.	**4m**	45.94 (±3.43)	63.5 (±4.22)	**1.38**
14.	**4n**	>100	>100	—
15.	**4o**	>100	>100	—
16.	**4p**	>100	>100	—
17.	**4q**	47.92 (±1.61)	>100	**2.09**
18.	**4r**	>100	>100	—
19.	**4s**	>100	>100	—
20.	**4t**	45.22 (±2.40)	67.7 (±2.18)	**1.50**
21.	**4u**	>100	>100	—
22.	**4v**	>100	>100	—
23.	**4w**	>100	>100	—
24.	**ART**	100	100	—
25.	**AS**	9.85	7.53	0.76
26.	**CQ**	100	3.07	0.03
27.	*Paclitaxel* ^c^	0.003	0.1	33.3

^a^ IC_50_: Concentration at which the inhibition of 50% cells was observed. Values expressed are means from at least three independent experiments conducted in duplicates; ^b^ S.I.: Selectivity index lung (IC_50_ for cytotoxicity against normal cells/IC_50_ for cytotoxicity against cancer cells); ^c^ See ref. [80].

**Table 3:** In vitro cytotoxicity of substituted spiro[pyrrolidine-2,3′-oxindoles] **4a**–**w** against human HepG2 cancer cell lines and the immortalized liver LO2 normal cell lines.

Entry	Trioxane	HepG2 Cells IC_50_ [µM] ^a^	LO2 Cells IC_50_ [µM] ^a^	S.I. ^b^
1.	**4a**	>100	>100	—
2.	**4b**	>100	>100	—
3.	**4c**	**60.28** (**±4.15**)	**37.6** (**±3.32**)	**0.62**
4.	**4d**	>100	>100	**—**
5.	**4e**	>100	81.26 (±0.97)	**0.81**
6.	**4f**	81.95 (±4.05)	>100	**—**
7.	**4g**	>100	>100	**—**
8.	**4h**	>100	>100	**—**
9.	**4i**	>100	>100	**—**
10.	**4j**	>100	>100	**—**
11.	**4k**	67.76 (±8.27)	33.32 (±4.02)	**0.49**
12.	**4l**	>100	>100	—
13.	**4m**	41.56 (±8.24)	36.65 (±2.20)	**0.88**
14.	**4n**	>100	>100	**—**
15.	**4o**	>100	>100	**—**
16.	**4p**	>100	>100	**—**
17.	**4q**	86.53 (±17.30)	46.8 (±9.17)	**0.54**
18.	**4r**	>100	>100	—
19.	**4s**	>100	>100	—
20.	**4t**	46.05 (±2.23)	45.49 (±2.50)	**0.99**
21.	**4u**	>100	>100	—
22.	**4v**	>100	>100	—
23.	**4w**	>100	>100	—
24.	**ART**	>100	>100	---
25.	**AS**	4.09 ± 0.4	8.25	2.01
26.	**CQ**	49.02 ± 0.4	15	0.30
27.	**Paclitaxel ^c^**	0.19 ± 0.4	<0.1	0.52

^a^ IC_50_: Concentration at which the inhibition of 50% cells was observed. Values expressed are means from at least three independent experiments conducted in duplicates; ^b^ S.I.: Selectivity index liver (IC_50_ for cytotoxicity against normal cells/IC_50_ for cytotoxicity against cancer cells); ^c^ See ref. [82].

## Data Availability

Not required.

## Supplementary Materials

The following supporting information can be downloaded at: https://www.mdpi.com/article/10.3390/molecules28186503/s1, Figure S1: The X-ray crystal structure of compound **4l** showing with ORTEP diagram using 30% ellipsoidal plot. Phenyl ring is found disorder due to presence of two major rotamer [C19, C20, C21, C22, C23 C24 and C19, C20A, C21A, C22, C23A, C24A]. Figure S2: Cytotoxicity against A459 cell lines. Figure S3: Cytotoxicity against BEAS2B cell lines. Figure S4: Cytotoxicity against HepG2 cell lines. Figure S5: Cytotoxicity against LO2 cell lines.
