# Supplementary material for: Chemo-/Regio-Selective Synthesis of Novel Functionalized Spiro[pyrrolidine-2,3′-oxindoles] under Microwave Irradiation and Their Anticancer Activity
Source: Molecules. 2023 Sep 7;28(18):6503. doi: 10.3390/molecules28186503 (PMC10537280; doi:10.3390/molecules28186503)

Supporting Information

Chemo-/Regio-selective Synthesis and Anticancer Activity of Novel Functionalized Spiro[pyrrolidine-2,3'-oxindoles] Under Microwave Irradiations

Richa Sharma [1], Lalit Yadav [1], Ali Adnan Nasim [2], Ravi Kant Yadav [1], Chen Ruihong [2], Neha Kumari [3], Fan Ruiqi [2], Ashoke Sharon [3], Nawal K. Sahu [1,4], Sirish K. Ippagunta [5], Paolo Coghi [2,6,*], Vincent Kam Wai Wong [7,*] and Sandeep Chaudhary [1,8,*]

[1]Laboratory of Organic and Medicinal Chemistry (OMC Lab), Department of Chemistry, Malaviya National Institute of Technology, Jawaharlal Nehru Marg, Jaipur-302017, India. Fax: 91-141-2529029; Tel: 91-141-2713319 (MNIT-J)

[2]State Key Laboratory of Quality Research in Chinese Medicine, Macau University of Science and Technology, Avenida Wai Long, Taipa, Macau, China.

[3]Department of Applied Chemistry, Birla Institute of Technology Mesra, Ranchi, Jharkhand-835215, India.

[4]Department of Chemistry, Engineering College, Bharatpur (A Constituent College of Rajasthan Technical University Kota), Bharatpur-321303, Rajasthan, India.

[5]Department of Biotechnology, All India Institute of Medical Sciences (AIIMS), New Delhi-110029, India.

[6]School of Pharmacy, Macau University of Science and Technology, Avenida wai long, Taipa, Macau 999078, China.

[7]Dr. Neher's Biophysics Laboratory for Innovative Drug Discovery, State Key Laboratory of Quality Research in Chinese Medicine, Macau University of Science and Technology, Avenida Wai Long, Taipa, Macau, China.

[8]Laboratory of Bioactive Heterocycles and Catalysis (BHC lab), Department of Medicinal Chemistry, National Institute of Pharmaceutical Education and Research-Raebareli (Transit Campus), Bijnor–Sisendi Road, Near CRPF Base Camp, Sarojini Nagar, Lucknow-226002, India. Tel: 91-522-2975587 (NIPER-R)

*****_Correspondence E-mail:_ coghips@must.edu.mo , bowaiwong@gmail.com , schaudhary.chy@mnit.ac.in , schaudhary.chy@niperraebareli.edu.in

Fax: 91-141-2529029; Tel: 91-141-2713319 (MNIT-J)

Tel: 91-522-2975587 (NIPER-R)

S. No.	Contents	Page No.
1.	Single Crystal X-ray data	S1-S2
2.	^{1}H and ^{13}C spectral data of Spiro[pyrrolidine-2,3’-oxindoles] (**4a-w)**	S3-S25
3.	Mass Spectrum of of Spiro[pyrrolidine-2,3’-oxindoles] **4a-w**	S26-S48
4.	Figure 2. Cytotoxicity against A459 cell lines	S-49
5.	Figure 3. Cytotoxicity against BEAS2B cell lines	S-50
6.	Figure 4. Cytotoxicity against HepG2 cell lines	S-51
7.	Figure 5. Cytotoxicity against LO2 cell lines	S-52

1. Single Crystal Data of cycloadduct 4l

CCDC NO:	2251566
Identification code	4l
Empirical formula	$C_{26}H_{25}N_3O_5$
Formula weight	459.49
Temperature/K	293(2)
Crystal system	Triclinic
Space group	P-1
a/Å	5.9974(3)
b/Å	11.0284(10)
c/Å	17.8408(19)
α/°	78.962(8)
β/°	86.539(7)
γ/°	80.455(7)
Volume/Å^3	1141.65(18)
Z	1
ρ_{calc}g/cm^3	0.946
μ/mm^{-1}	0.047
F(000)	242.0
D_c /Mg m^{-1}	1.321
Crystal size/mm^3	0.20 × 0.15 × 0.10
Reflections measured	9585
Final R indexes [I>=2σ (I)]	R_1 = 0.0476
Final R indexes [all data]	R_1 = 0.0627
Flack parameter	0.7(4)

The Unit cell determination and intensity data collection was performed at 293 (2) K. The structure solutions by direct methods and refinements was performed by full-matrix least-squares methods on F_2. The flack parameter of the crystal structure obtained is 0.7(4), which shows that the crystal is a mixture of stereoisomers.

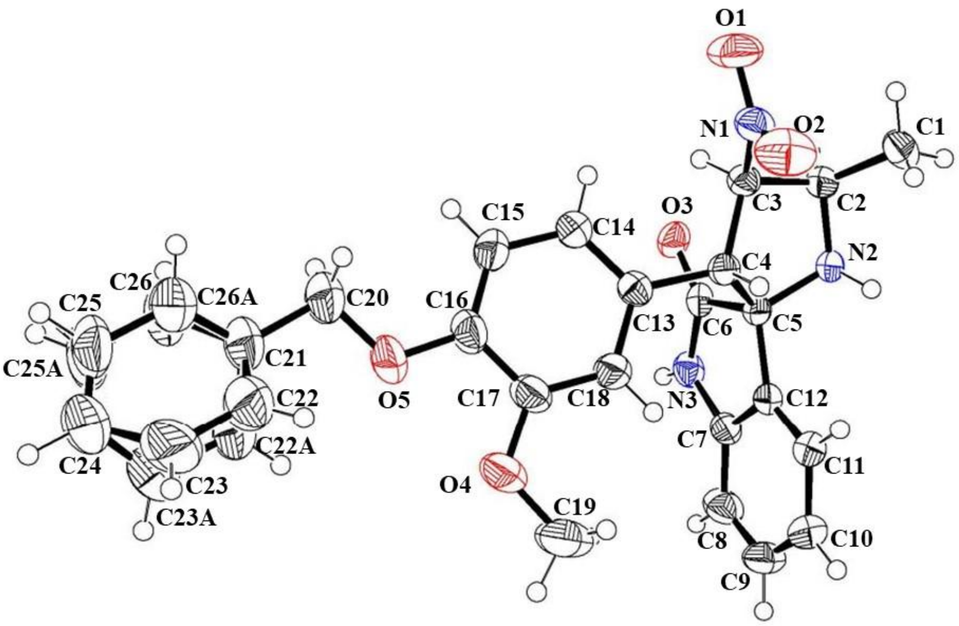

Figure 1. The X-ray crystal structure of compound **4l** showing with ORTEP diagram using 30% ellipsoidal plot. Phenyl ring is found disorder due to presence of two major rotamer [C19, C20, C21, C22, C23 C24 and C19, C20A, C21A, C22, C23A, C24A].

Characterization spectral data (^{1}HNMR and ^{13}CNMR) of Spiro[pyrrolidine-2,3'-oxindoles] 4a-w.

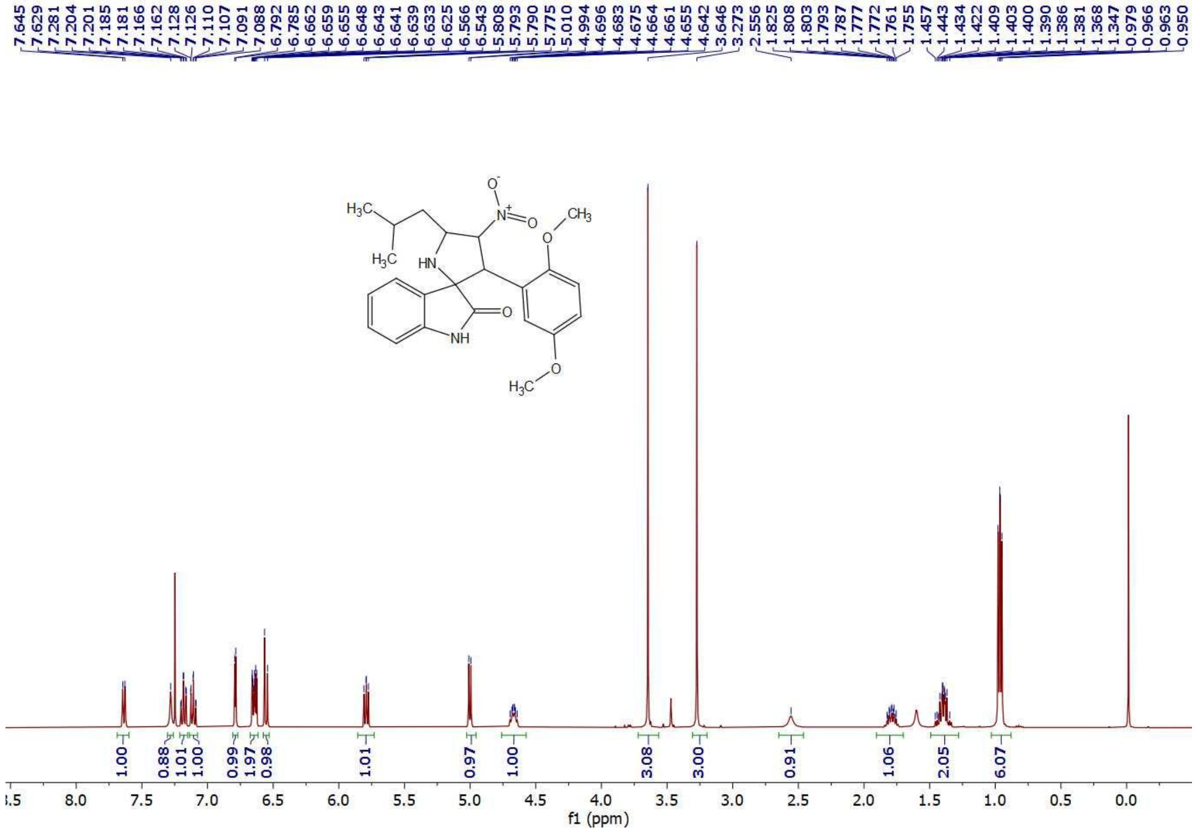

^{1}H-NMR (400 MHz, chloroform-*d*) spectrum of compound **4a**

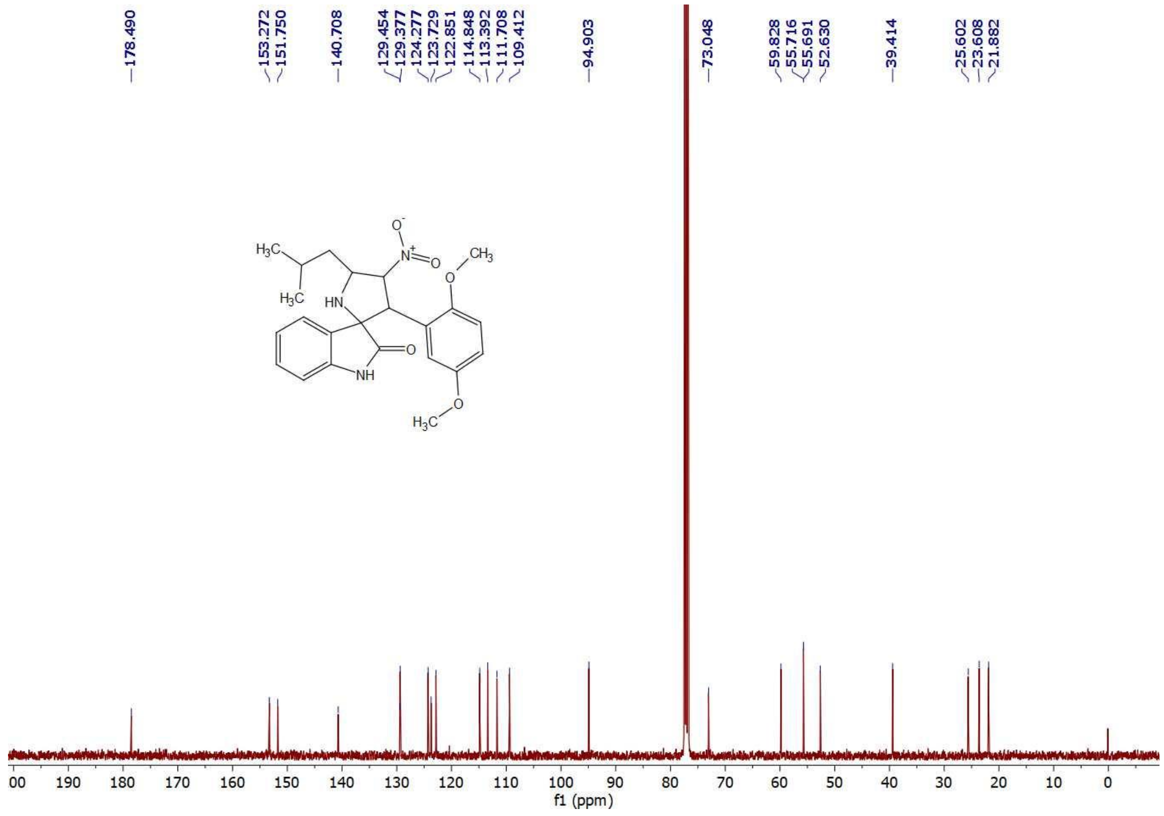

^{13}C-NMR (100 MHz, chloroform-*d*) spectrum of compound **4a**

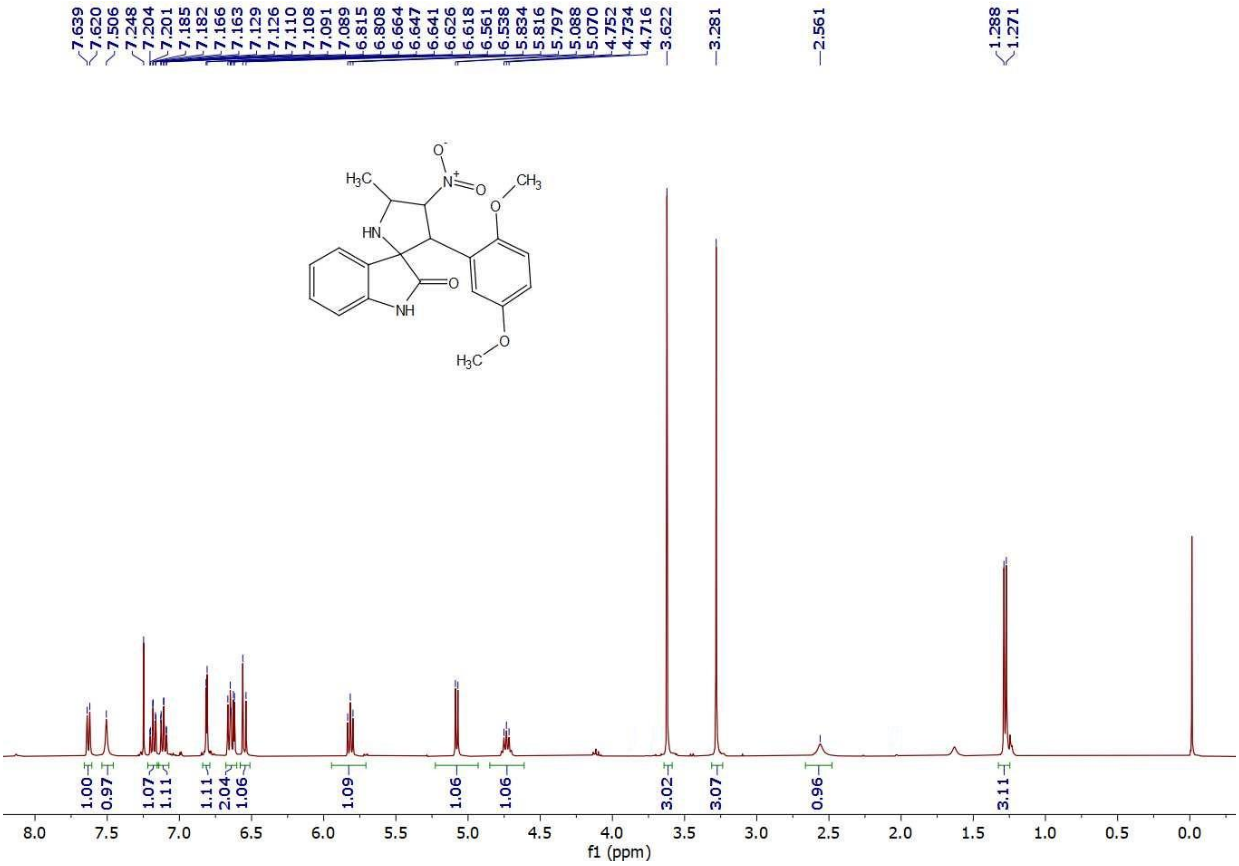

^{1}H-NMR (400 MHz, chloroform-*d*) spectrum of compound **4b**

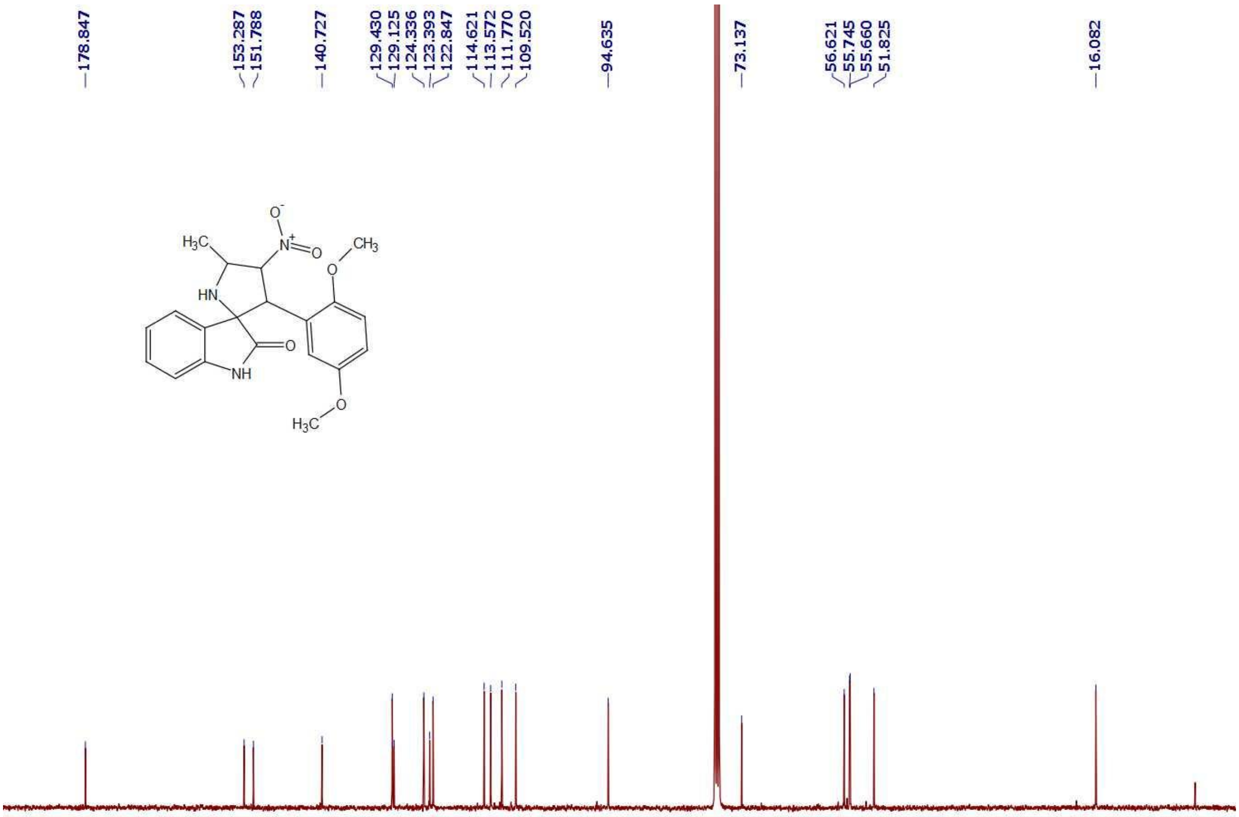

^{13}C-NMR (100 MHz, chloroform-*d*) spectrum of compound **4b**

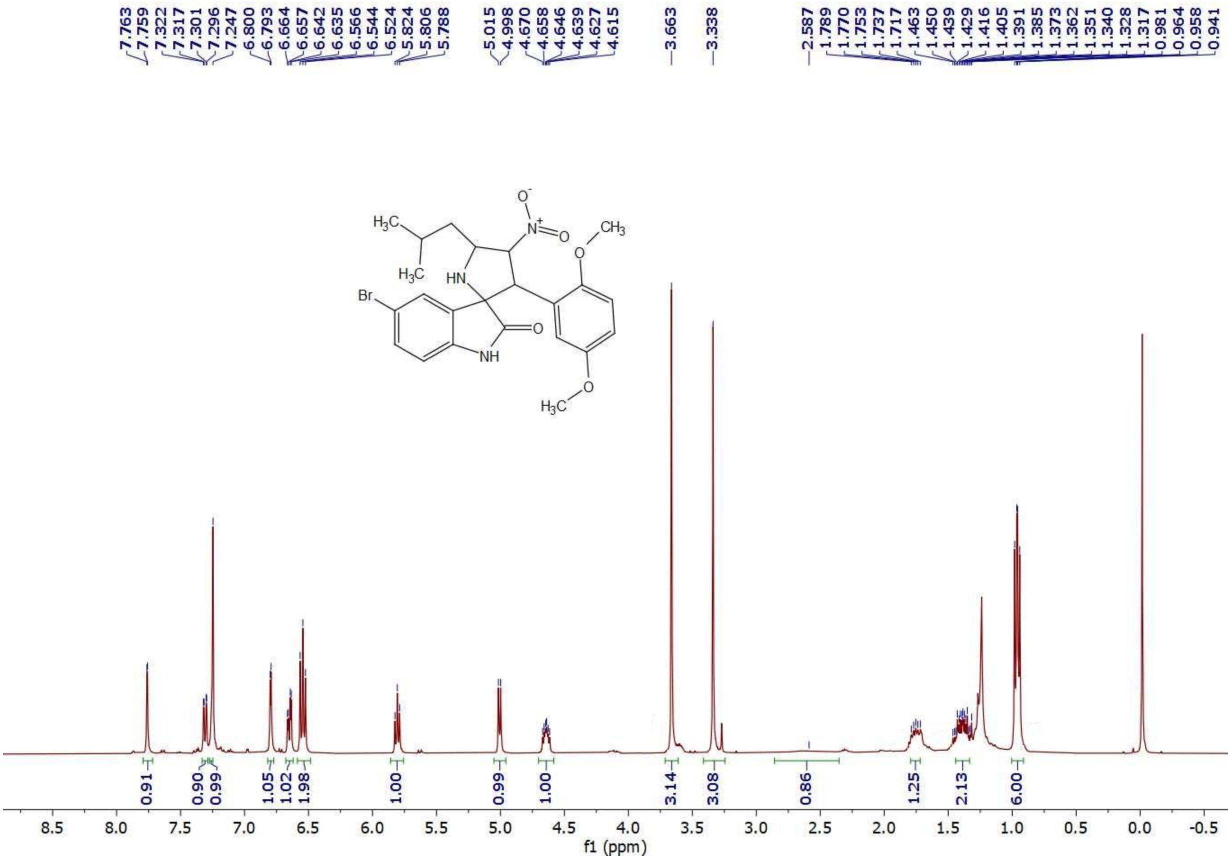

^{1}H-NMR (400 MHz, chloroform-*d*) spectrum of compound **4c**

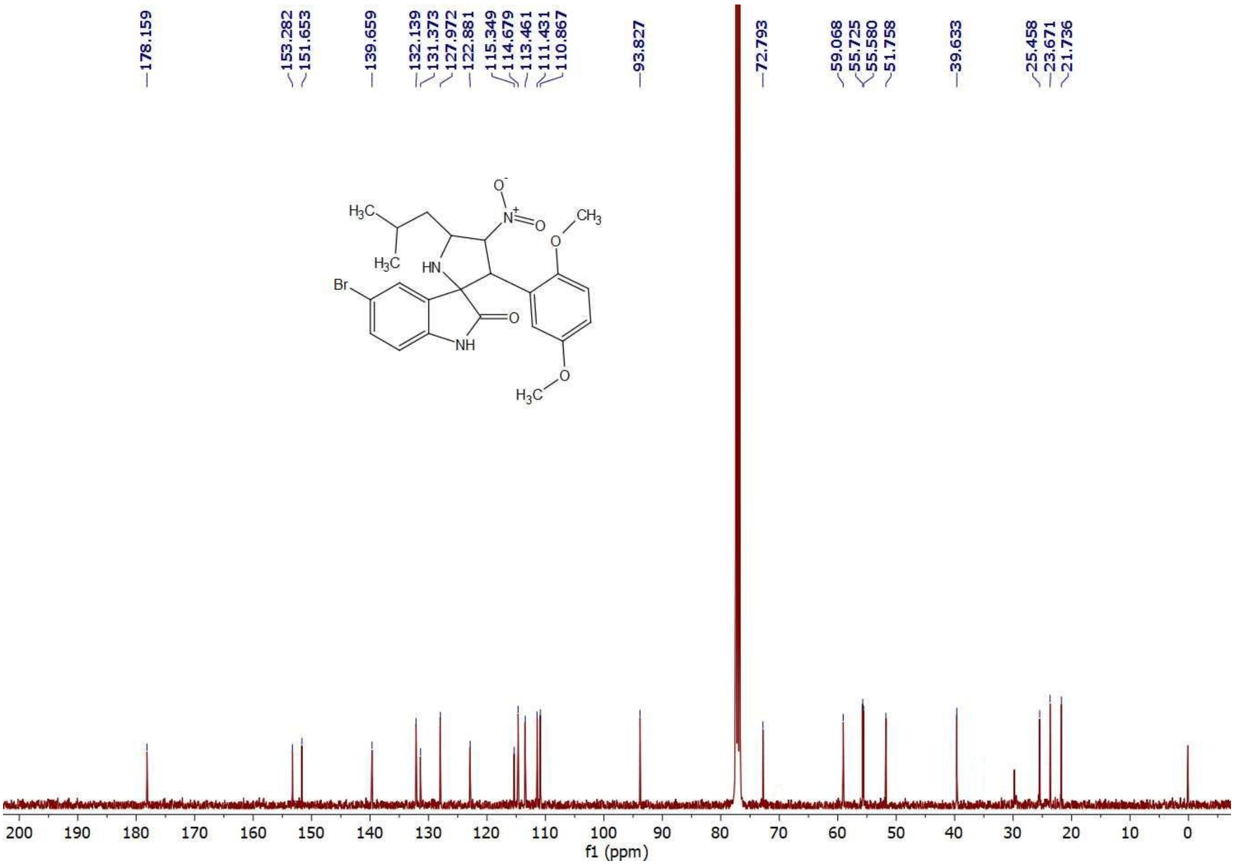

^{13}C-NMR (100 MHz, chloroform-*d*) spectrum of compound **4c**

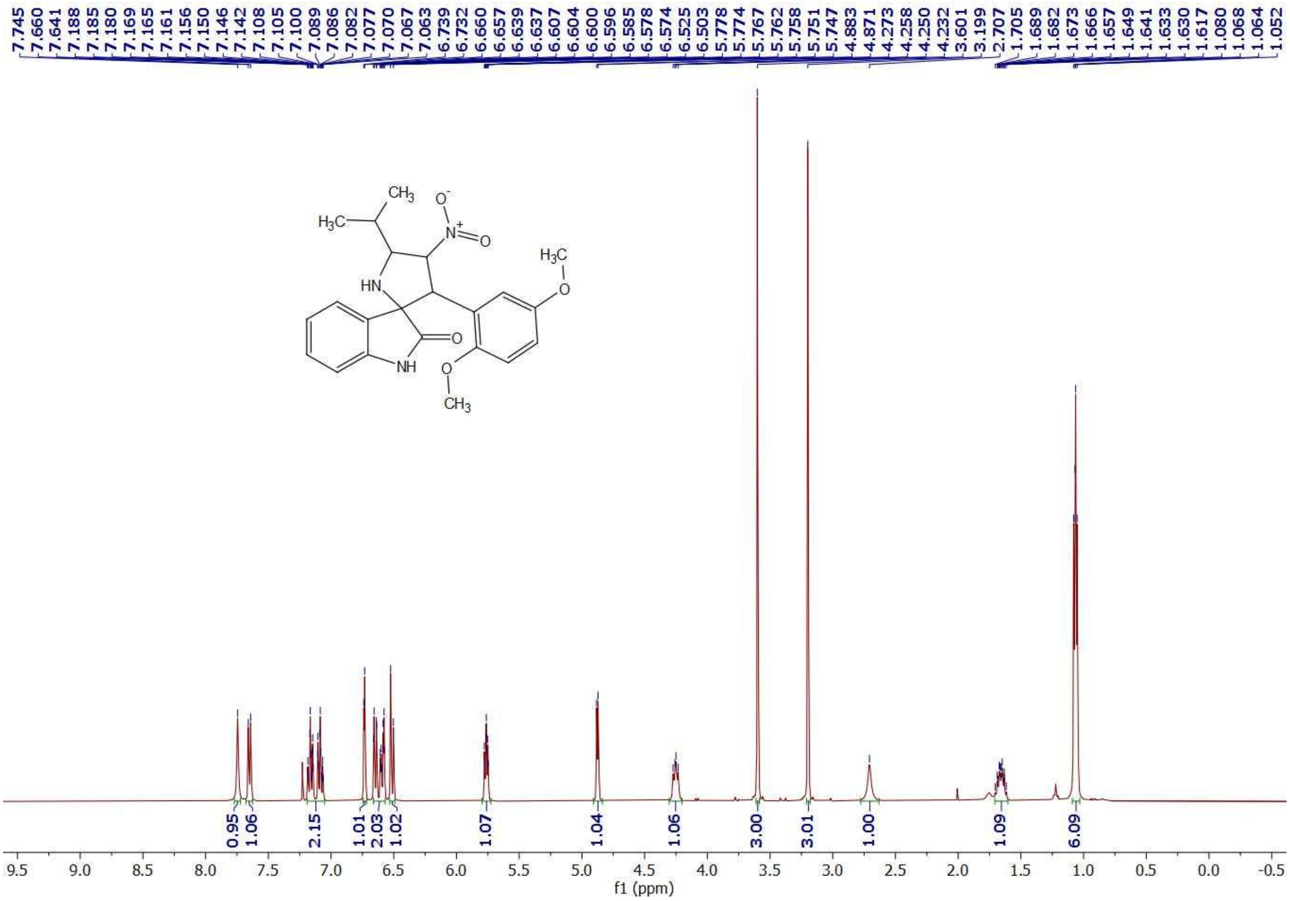

^{1}H-NMR (400 MHz, chloroform-*d*) spectrum of compound **4d**

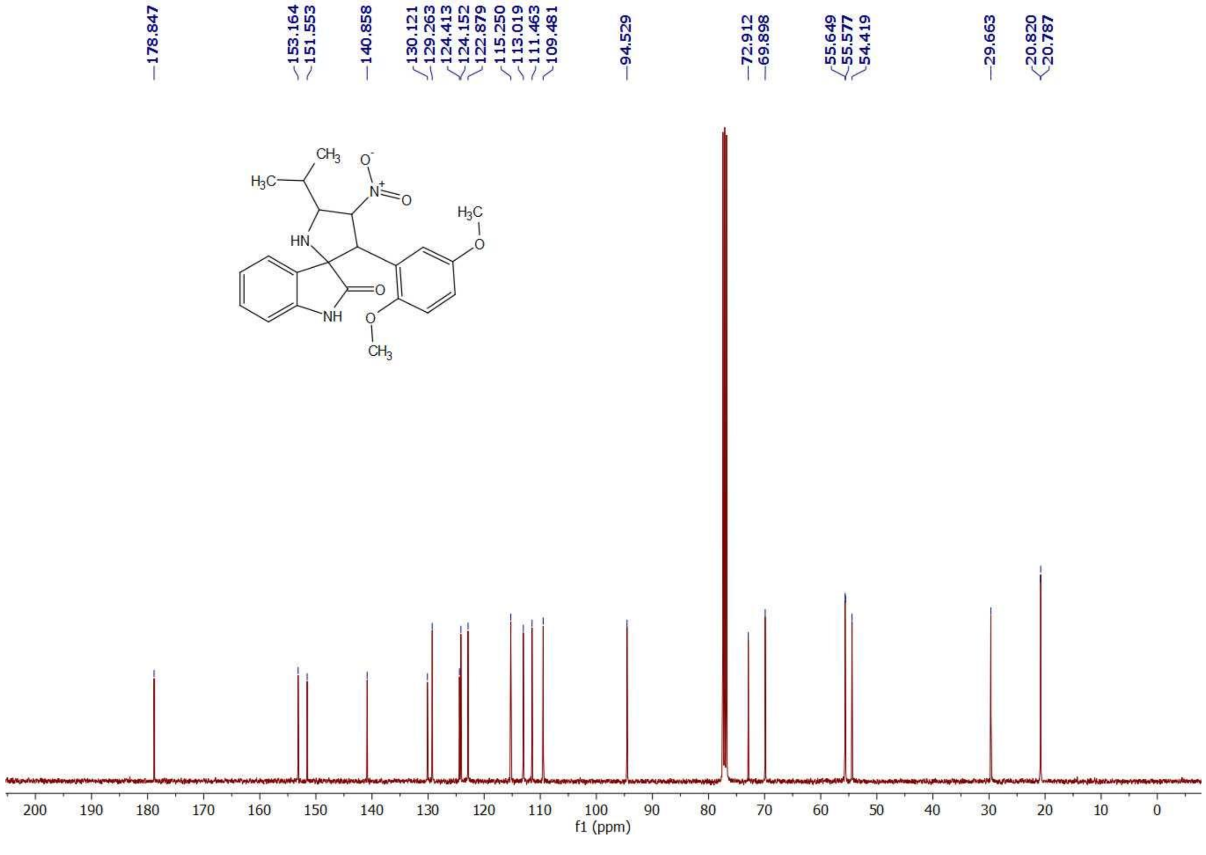

^{13}C-NMR (100 MHz, chloroform-*d*) spectrum of compound **4d**

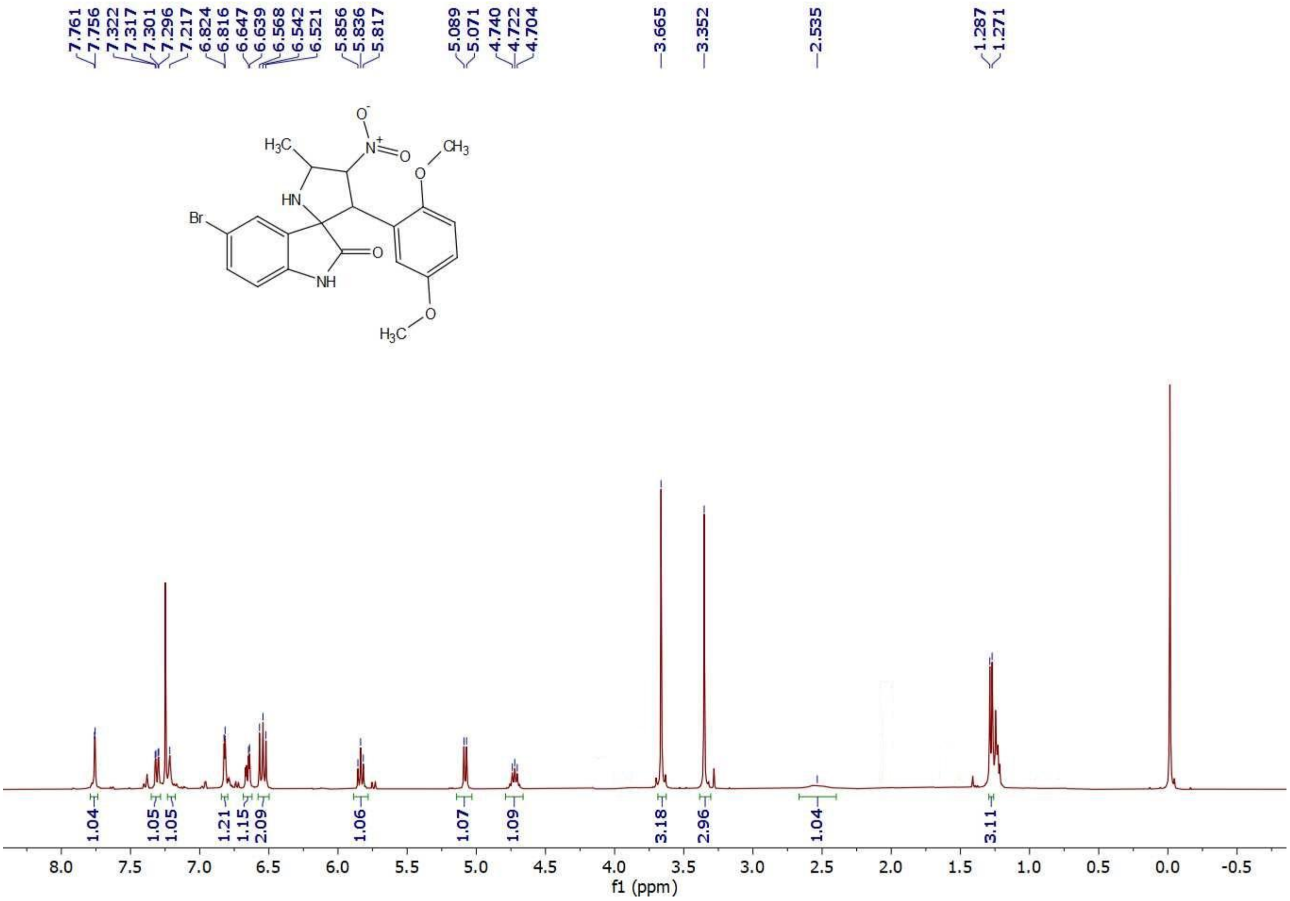

^{1}H-NMR (400 MHz, chloroform-*d*) spectrum of compound **4e**

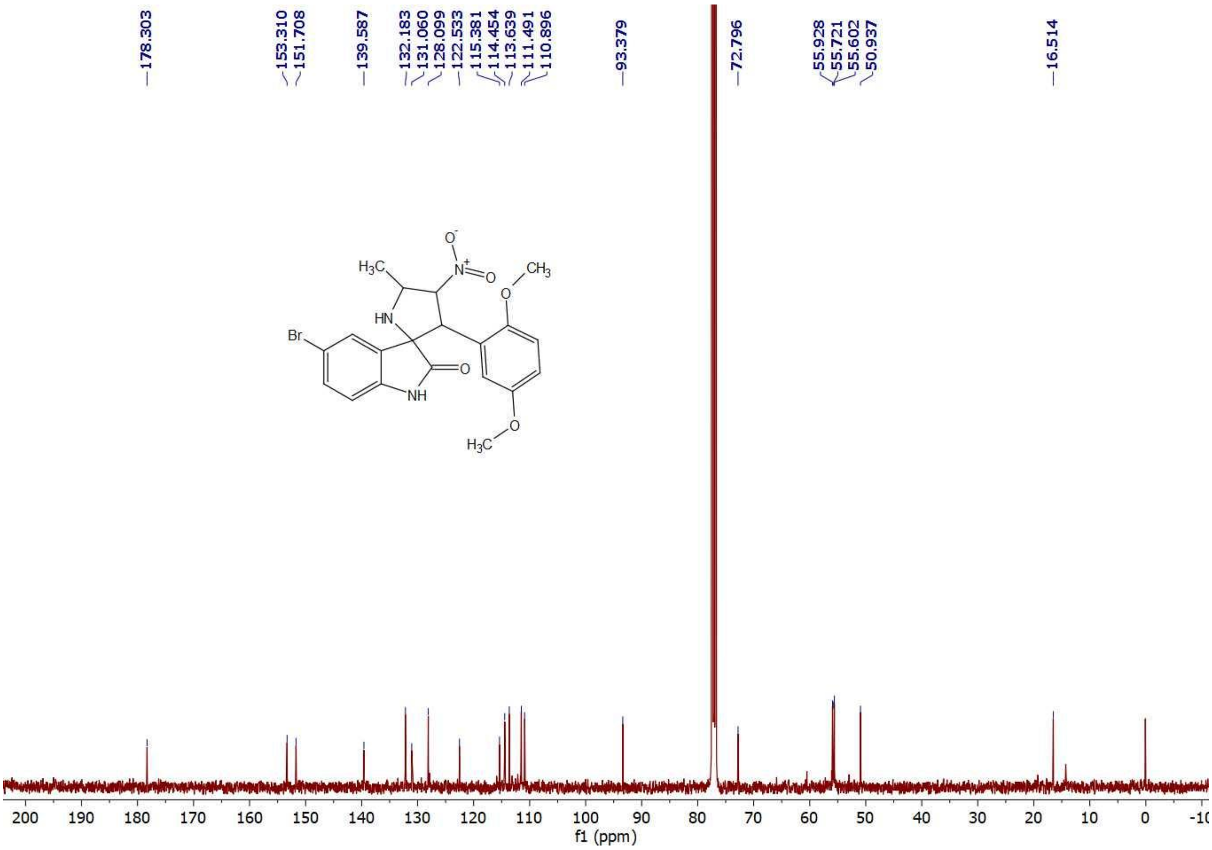

^{13}C-NMR (100 MHz, chloroform-*d*) spectrum of compound **4e**

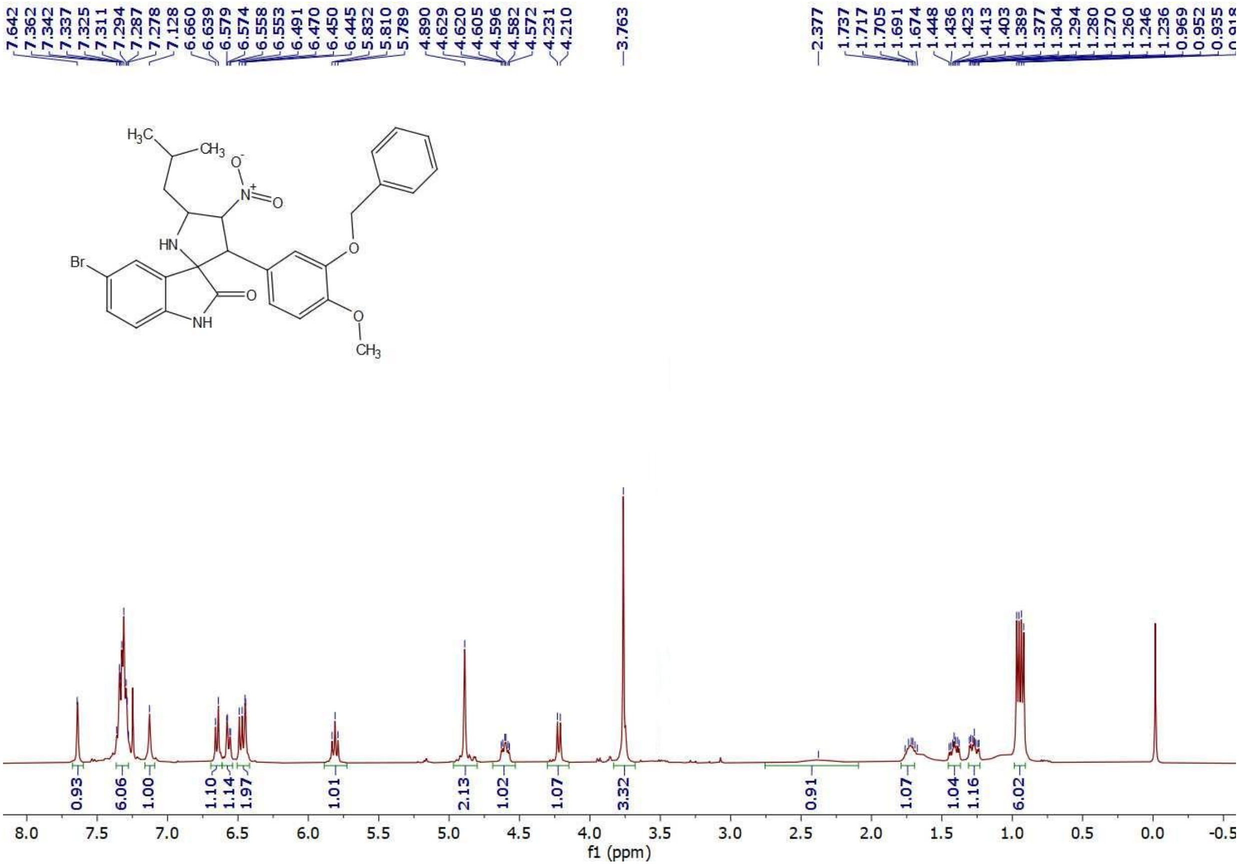

^{1}H-NMR (400 MHz, chloroform-*d*) spectrum of compound **4f**

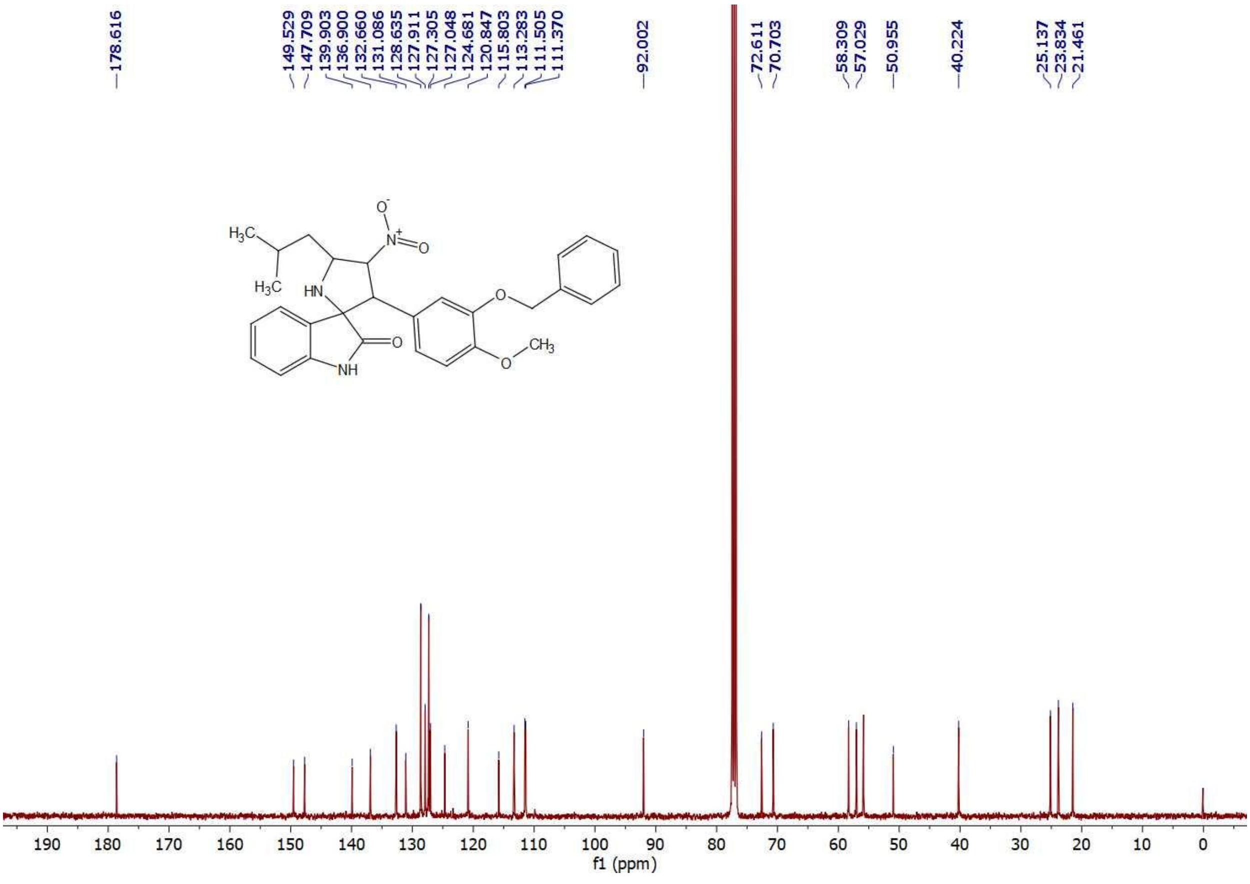

^{13}C-NMR (100 MHz, chloroform-*d*) spectrum of compound **4f**

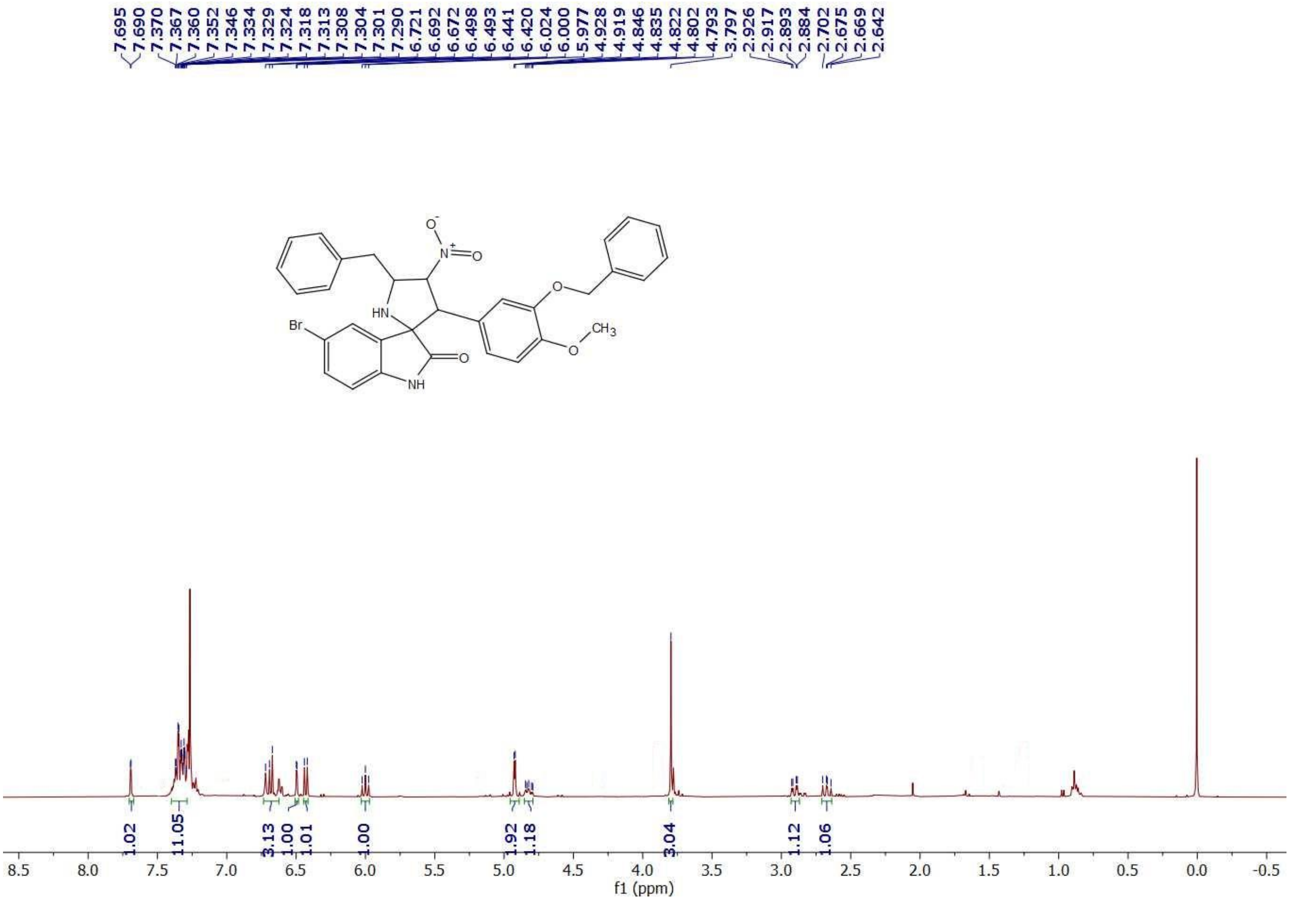

^{1}H-NMR (400 MHz, chloroform-*d*) spectrum of compound **4g**

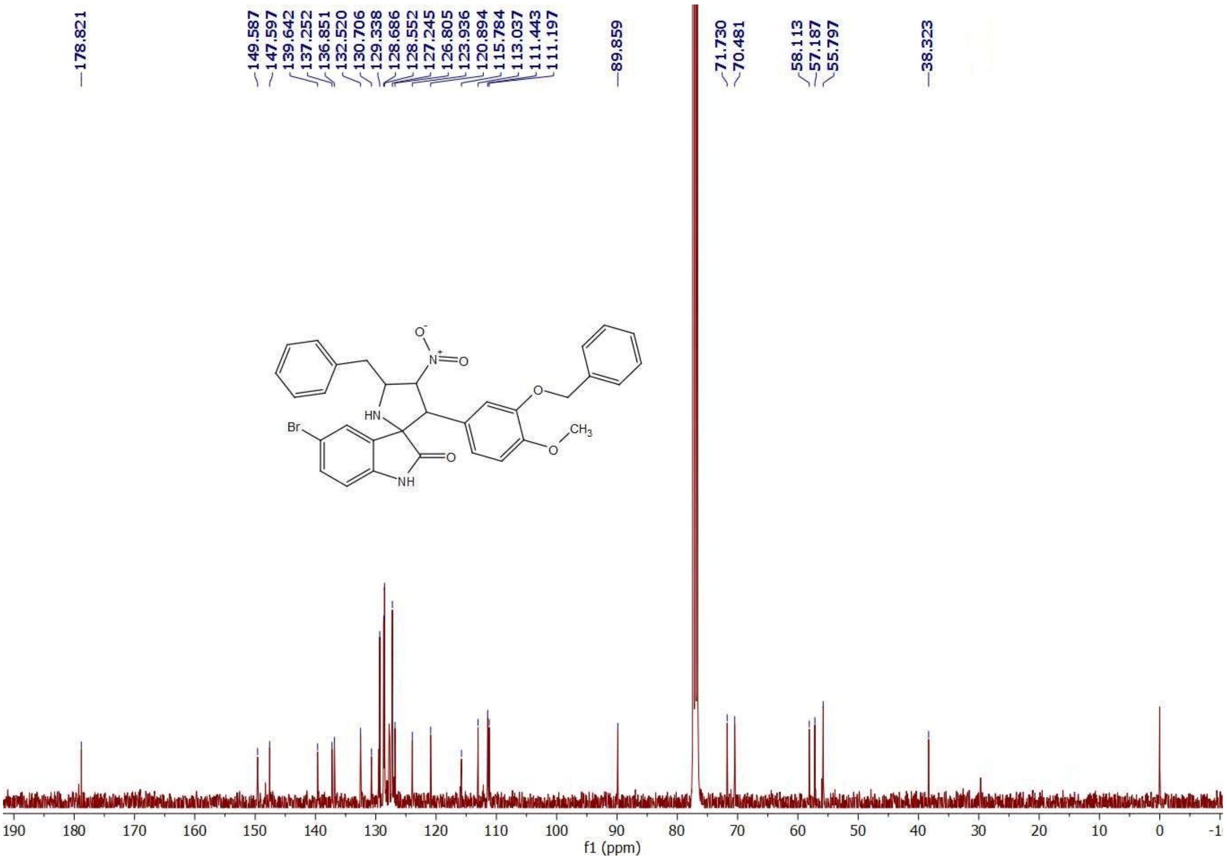

^{13}C-NMR (100 MHz, chloroform-*d*) spectrum of compound **4g**

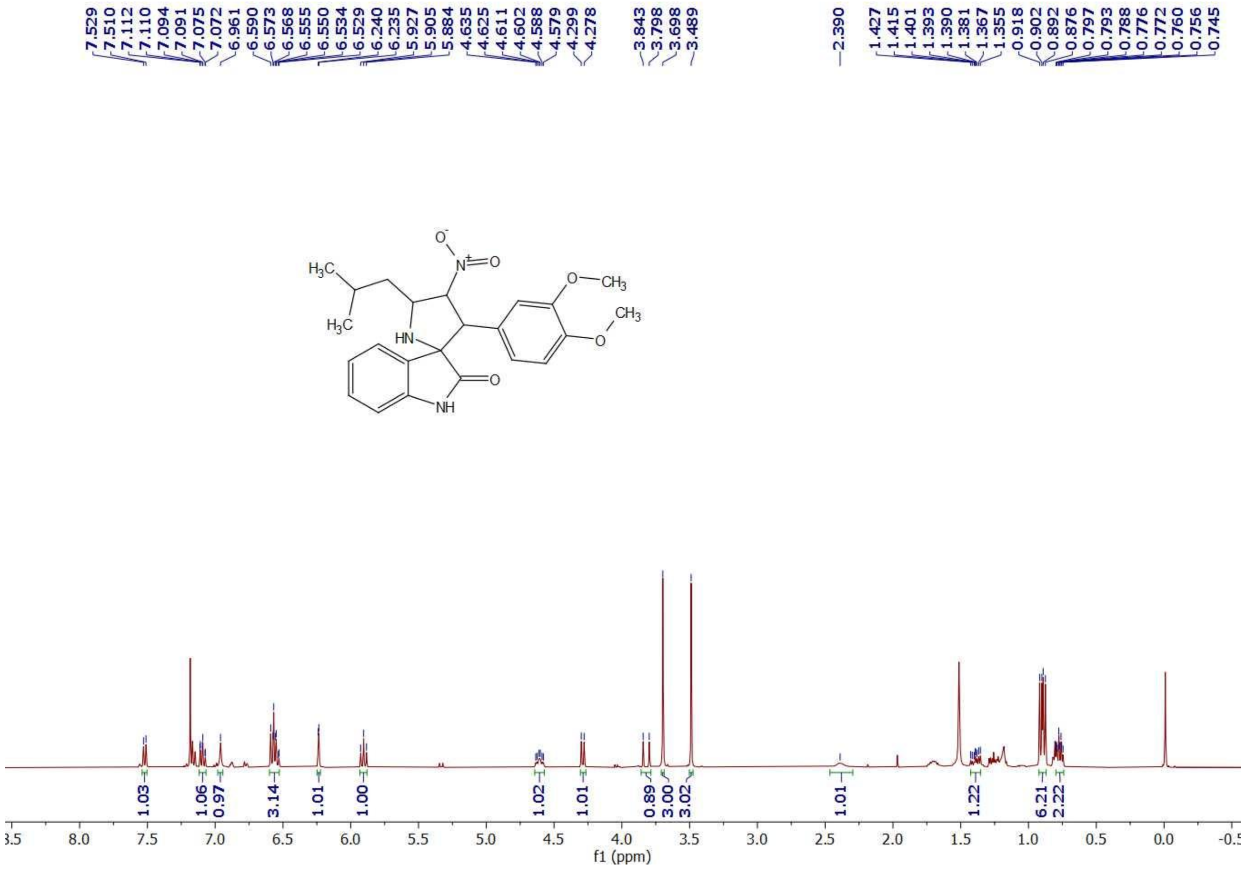

^{1}H-NMR (400 MHz, chloroform-*d*) spectrum of compound **4h**

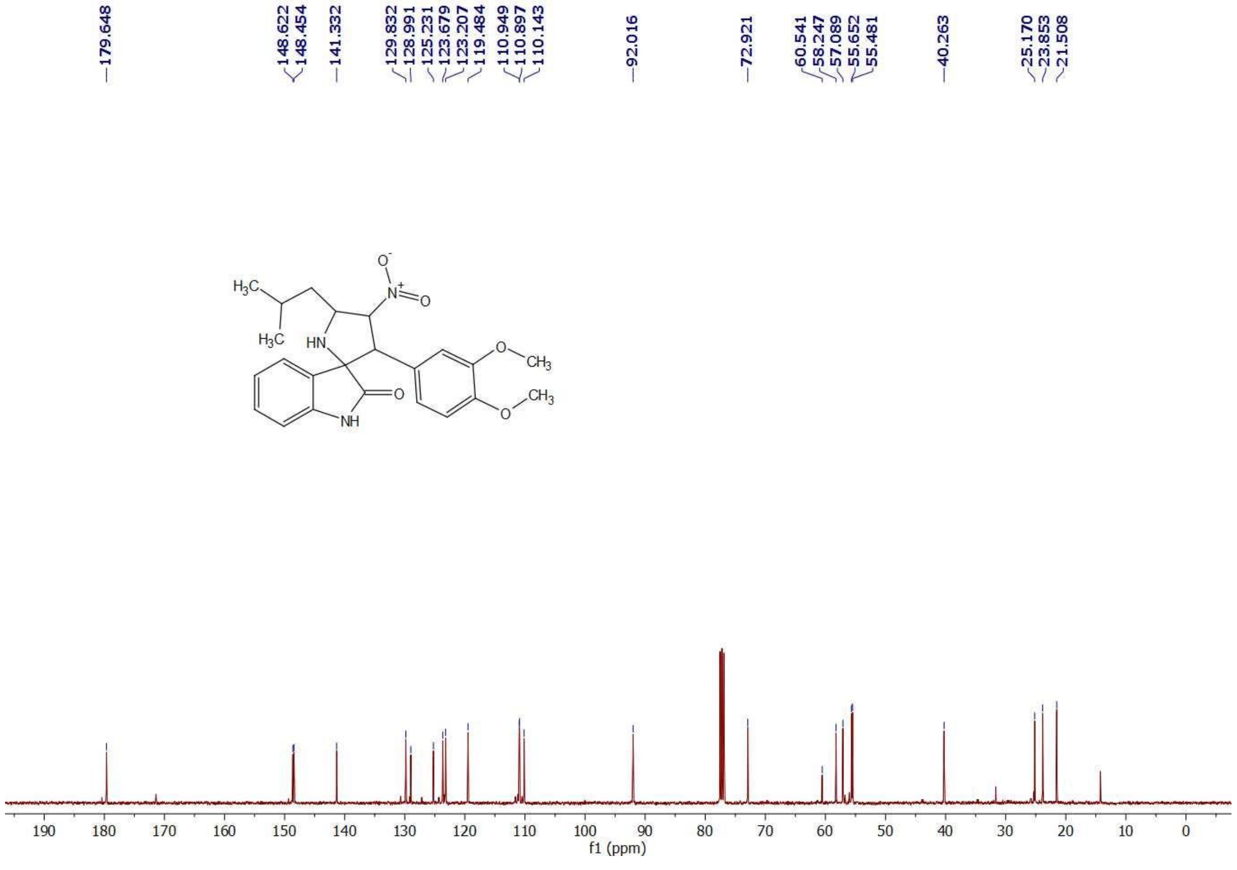

^{13}C-NMR (100 MHz, chloroform-*d*) spectrum of compound **4h**

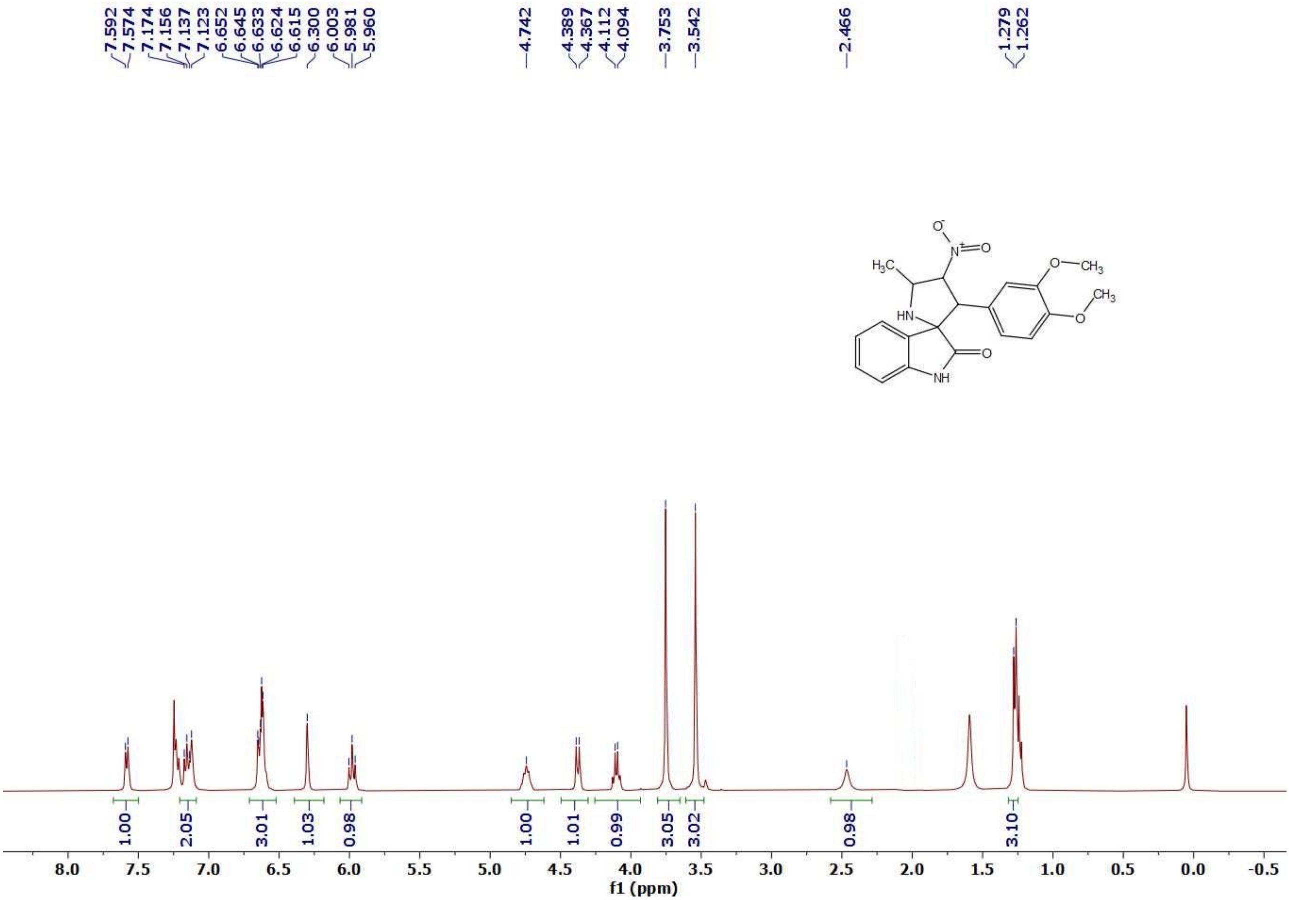

^{1}H-NMR (400 MHz, chloroform-*d*) spectrum of compound **4i**

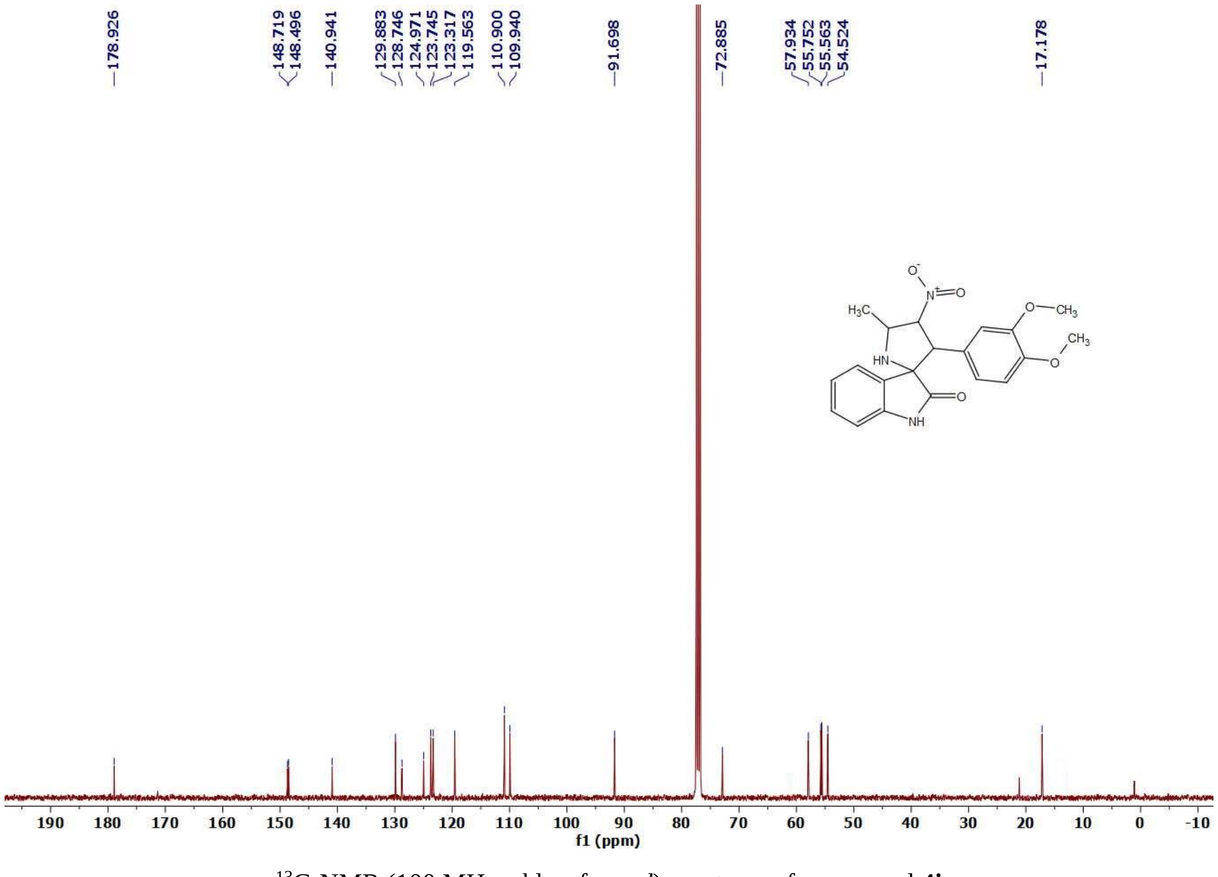

^{13}C-NMR (100 MHz, chloroform-*d*) spectrum of compound **4i**

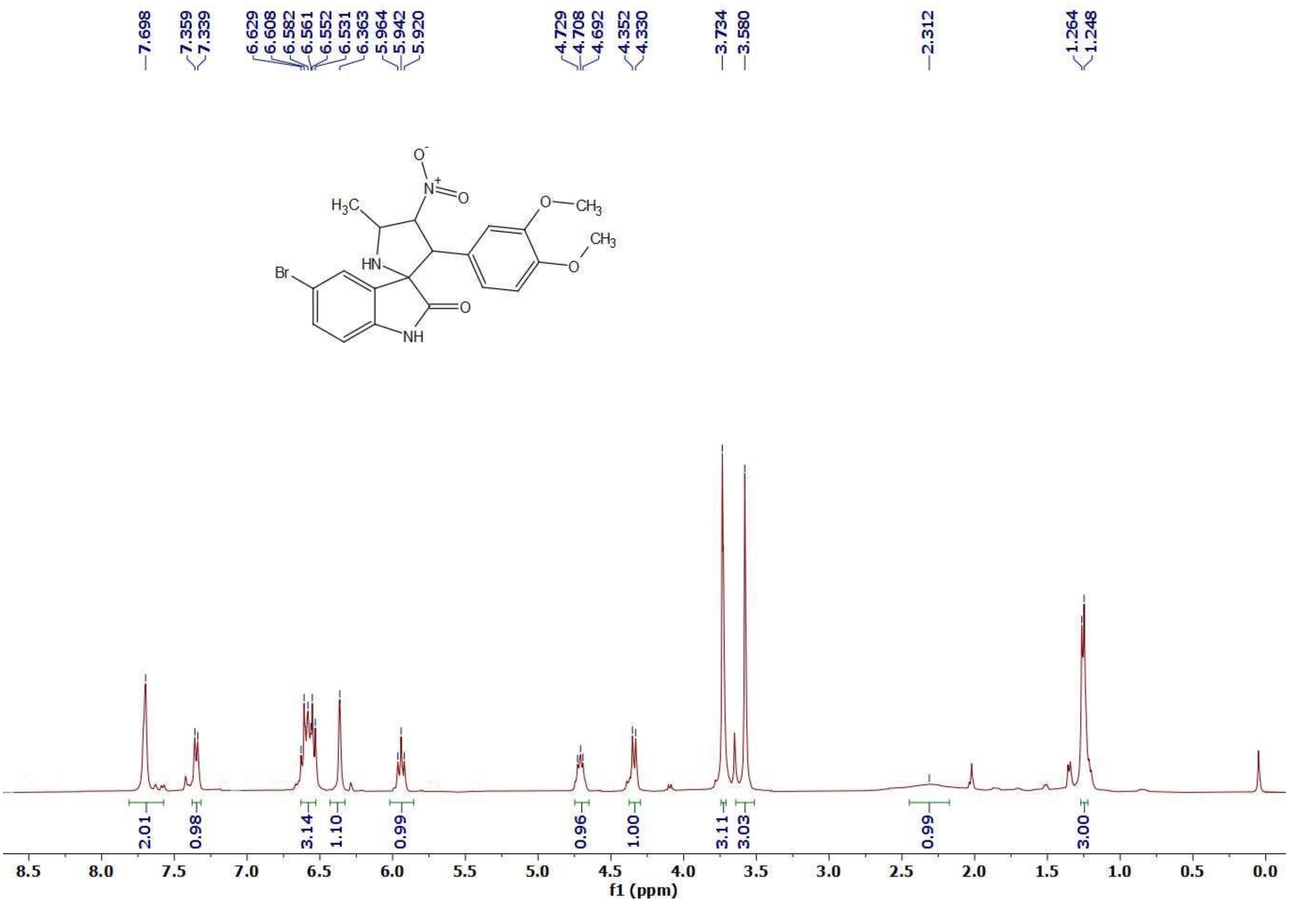

^{1}H-NMR (400 MHz, chloroform-*d*) spectrum of compound **4j**

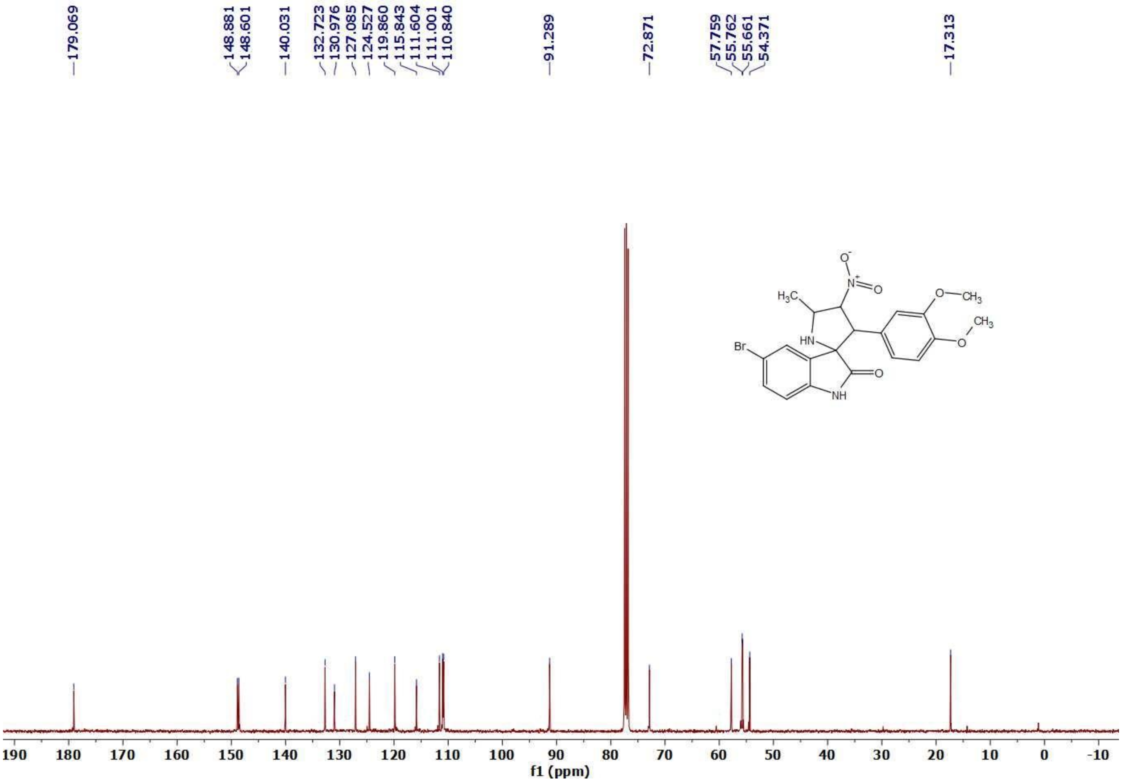

^{13}C-NMR (100 MHz, chloroform-*d*) spectrum of compound **4j**

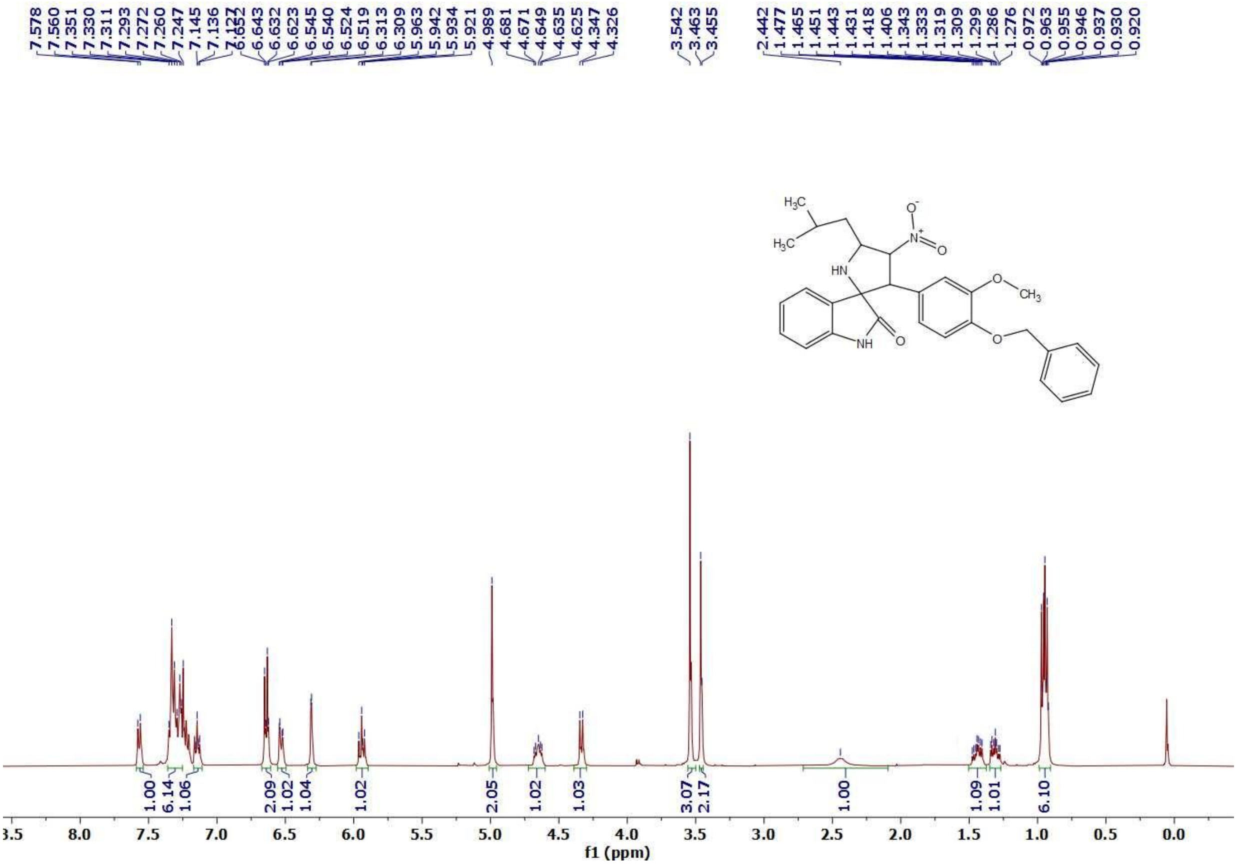

^{1}H-NMR (400 MHz, chloroform-*d*) spectrum of compound **4k**

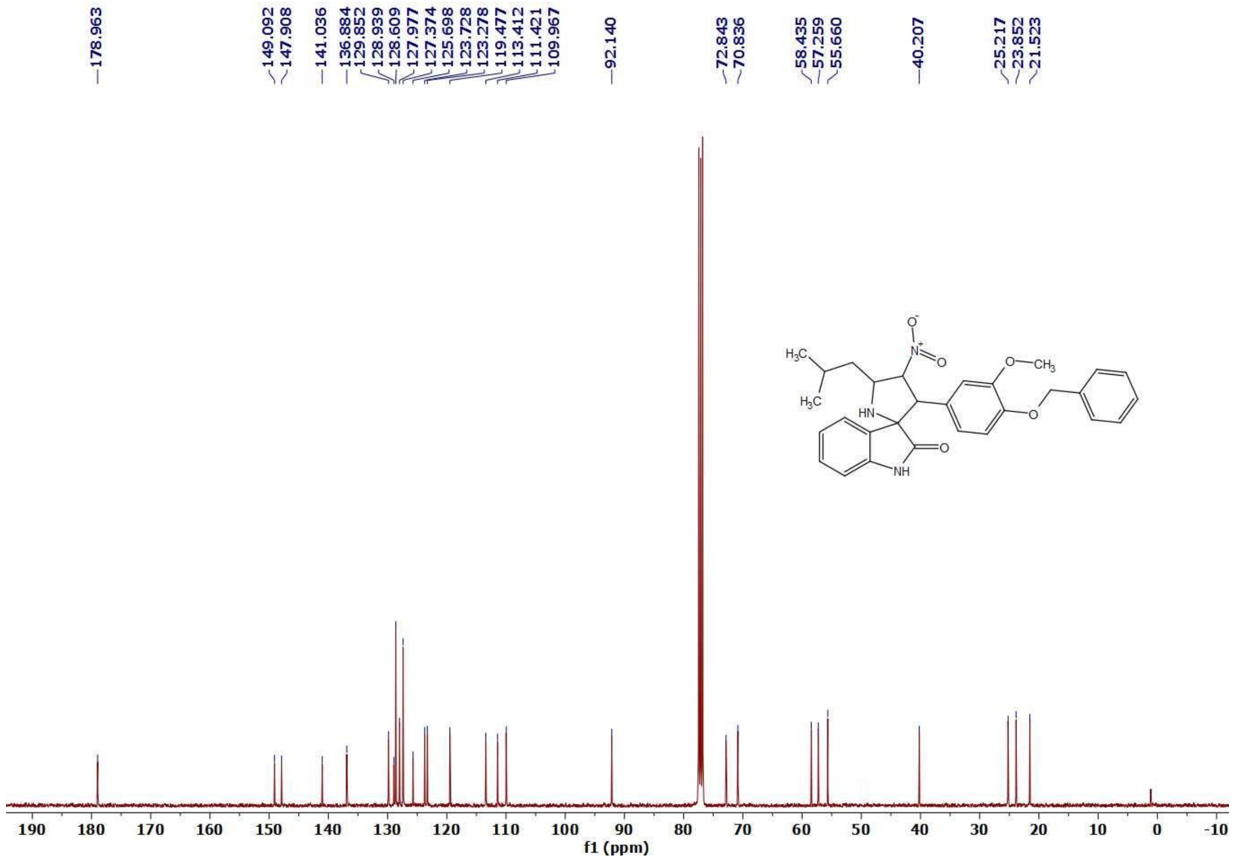

^{13}C-NMR (100 MHz, chloroform-*d*) spectrum of compound **4k**

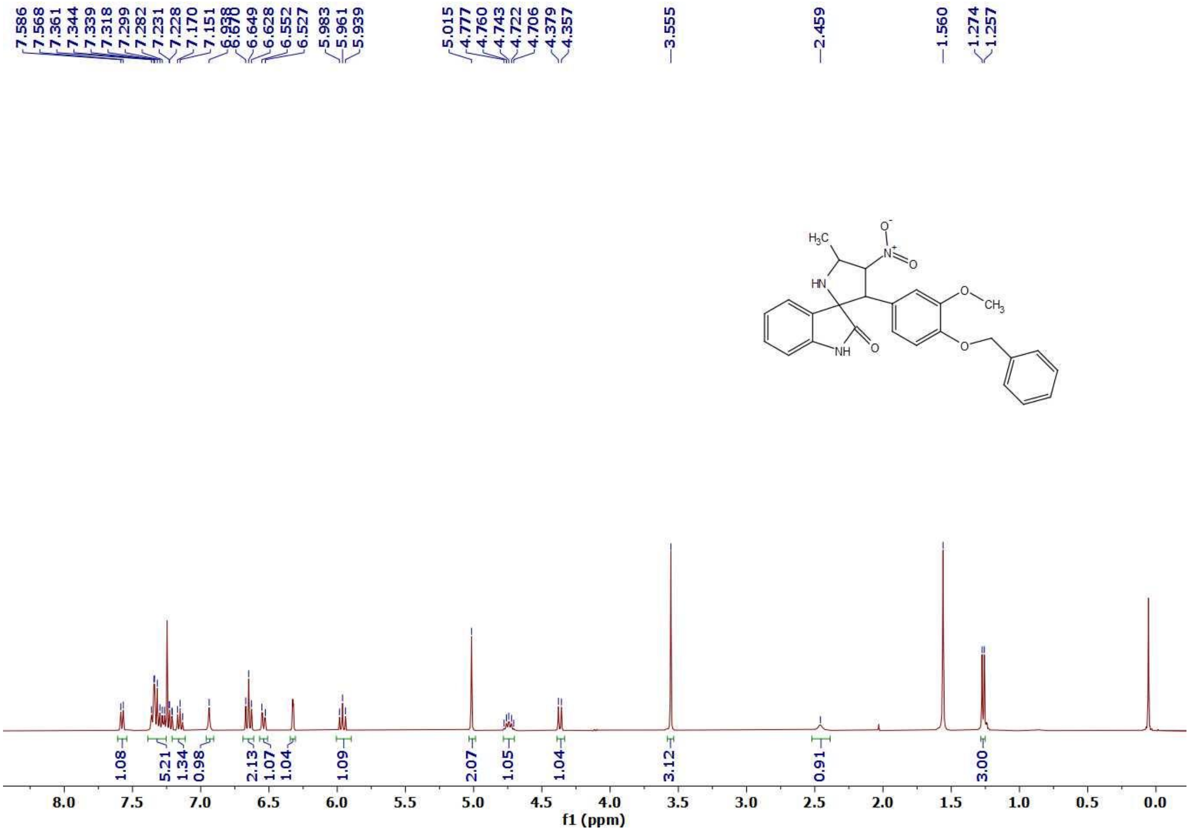

^{1}H-NMR (400 MHz, chloroform-*d*) spectrum of compound **4l**

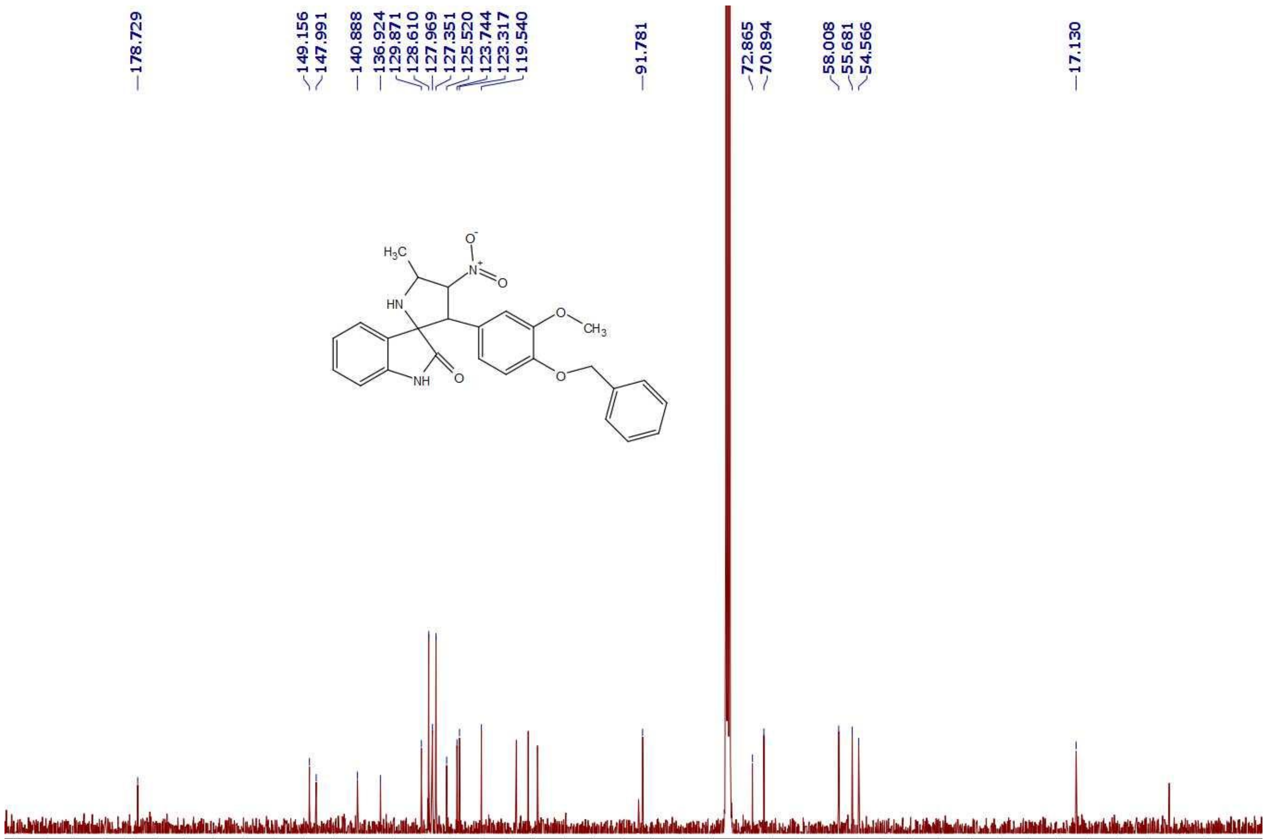

^{13}C-NMR (100 MHz, chloroform-*d*) spectrum of compound **4l**

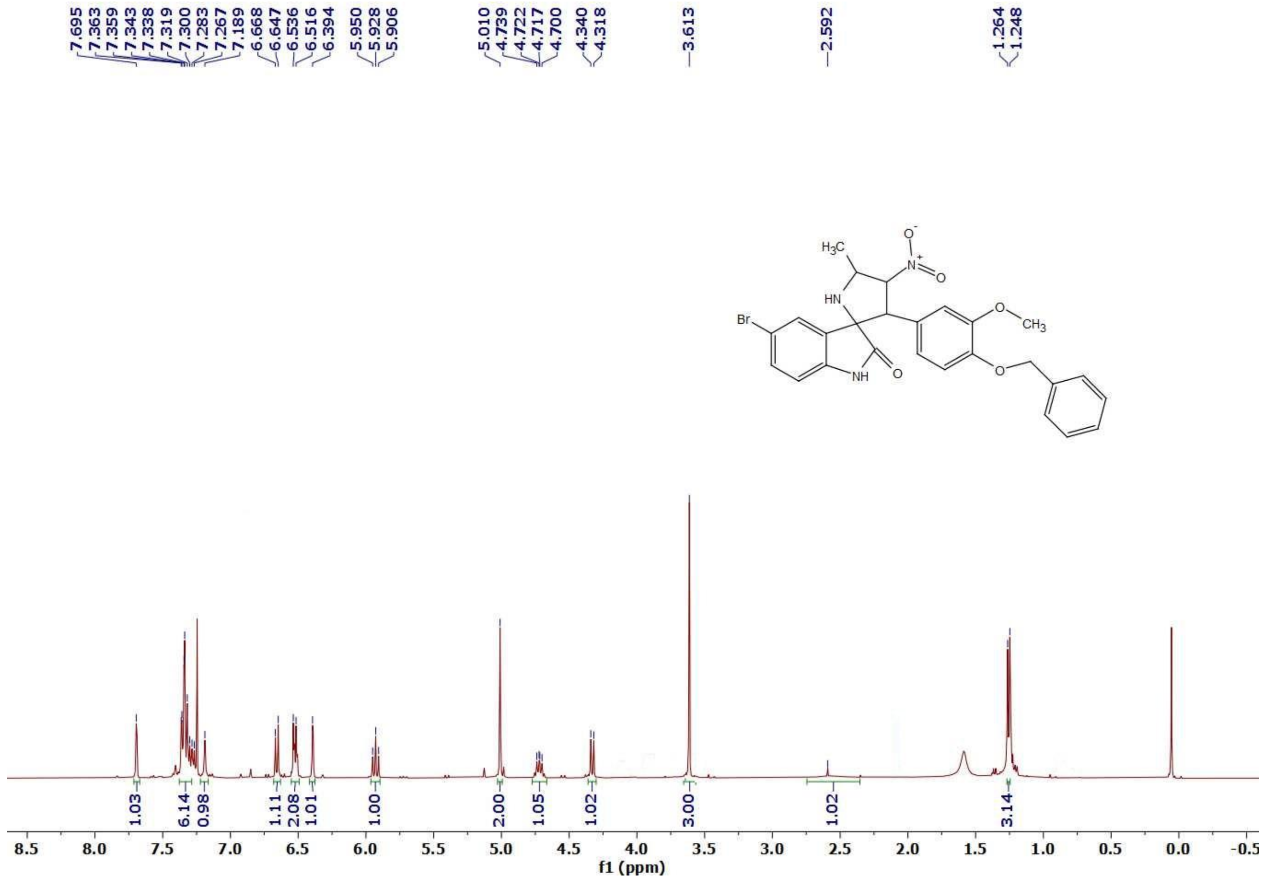

^{1}H-NMR (400 MHz, chloroform-*d*) spectrum of compound **4m**

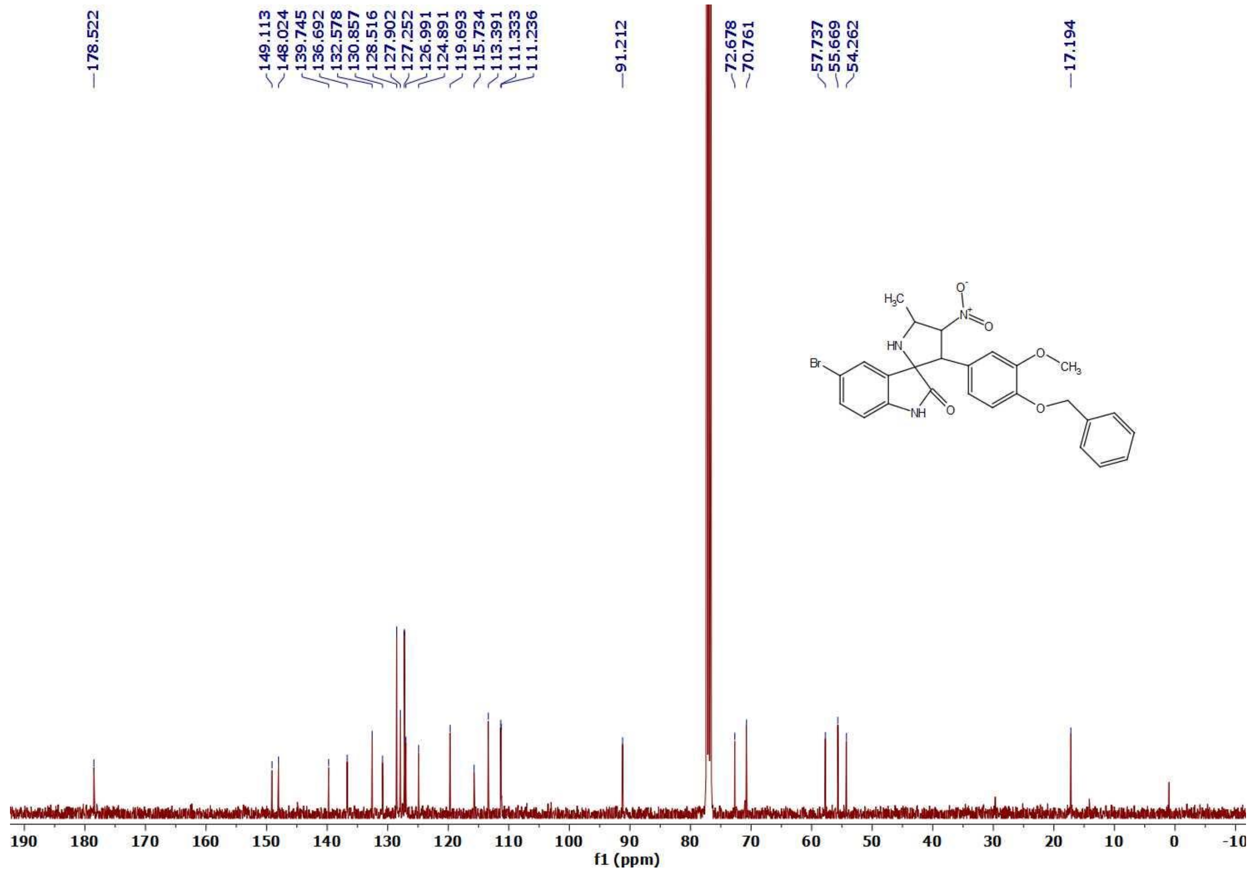

^{13}C-NMR (100 MHz, chloroform-*d*) spectrum of compound **4m**

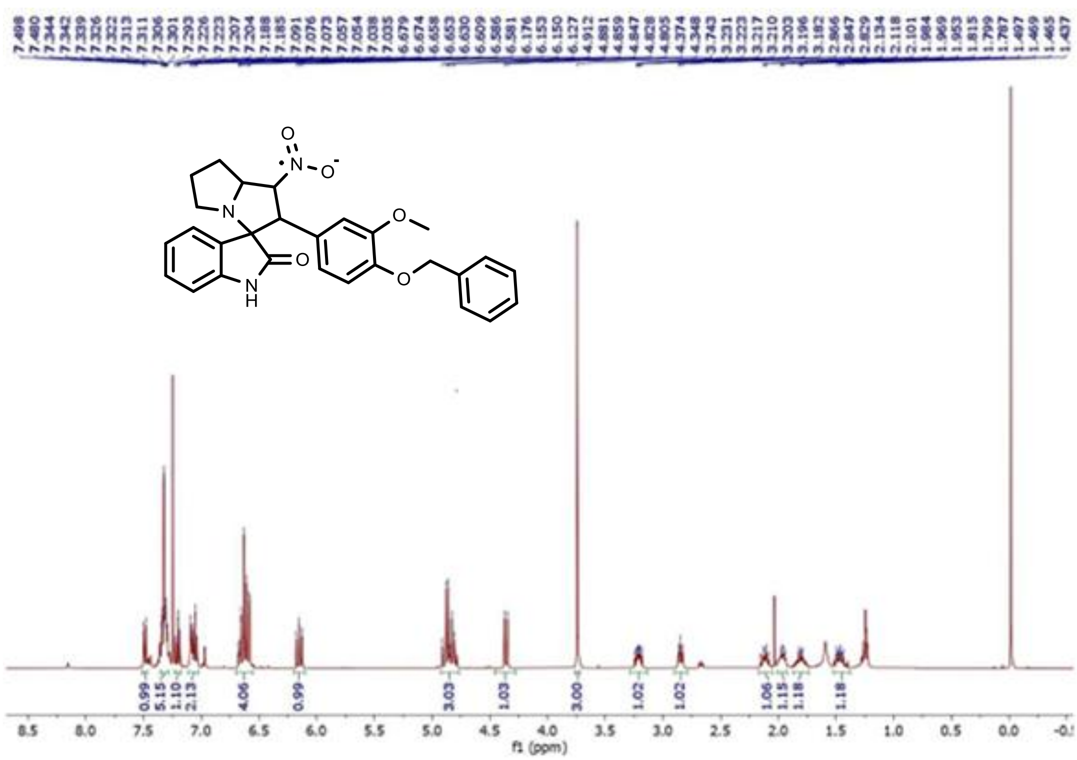

^{1}H-NMR (400 MHz, chloroform-*d*) spectrum of compound **4n**

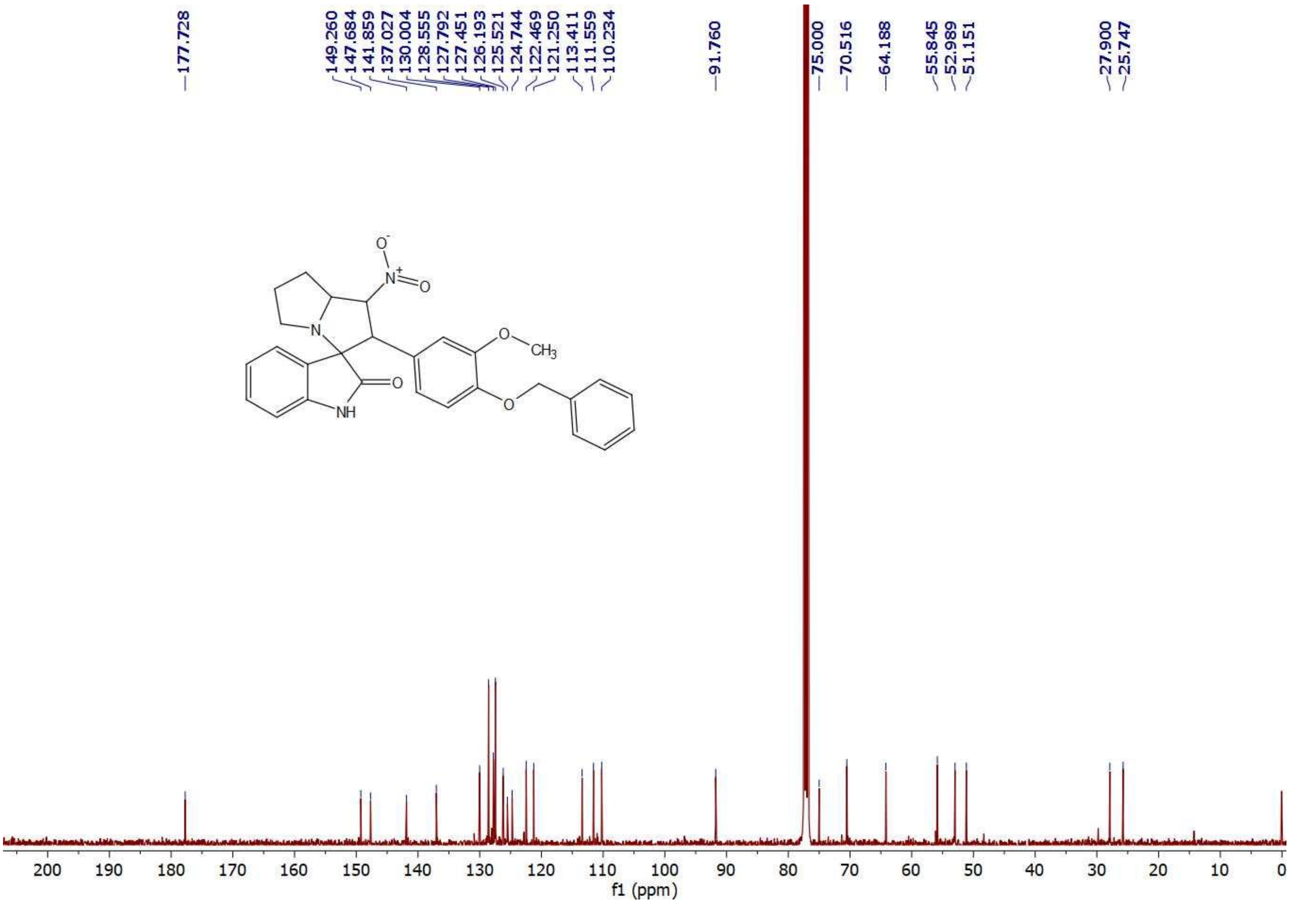

^{13}C-NMR (100 MHz, chloroform-*d*) spectrum of compound **4n**

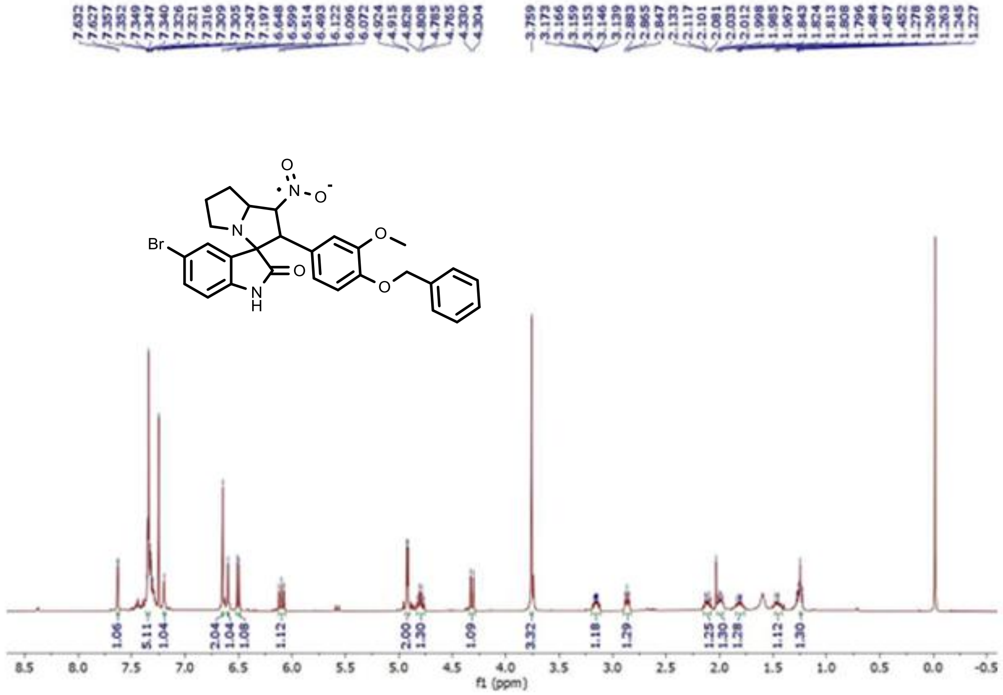

^{1}H-NMR (400 MHz, chloroform-*d*) spectrum of compound **4o**

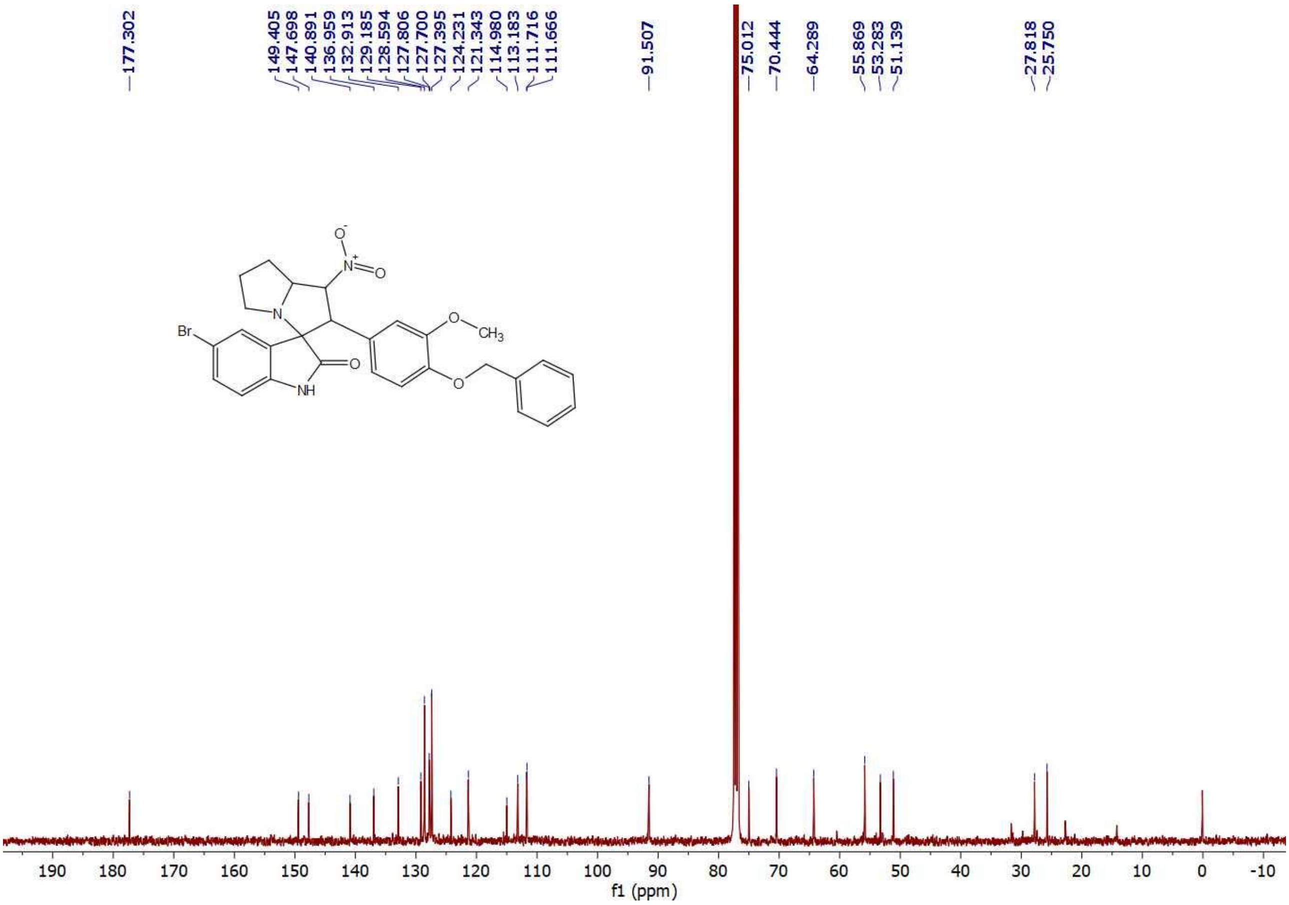

^{13}C-NMR (100 MHz, chloroform-*d*) spectrum of compound **4o**

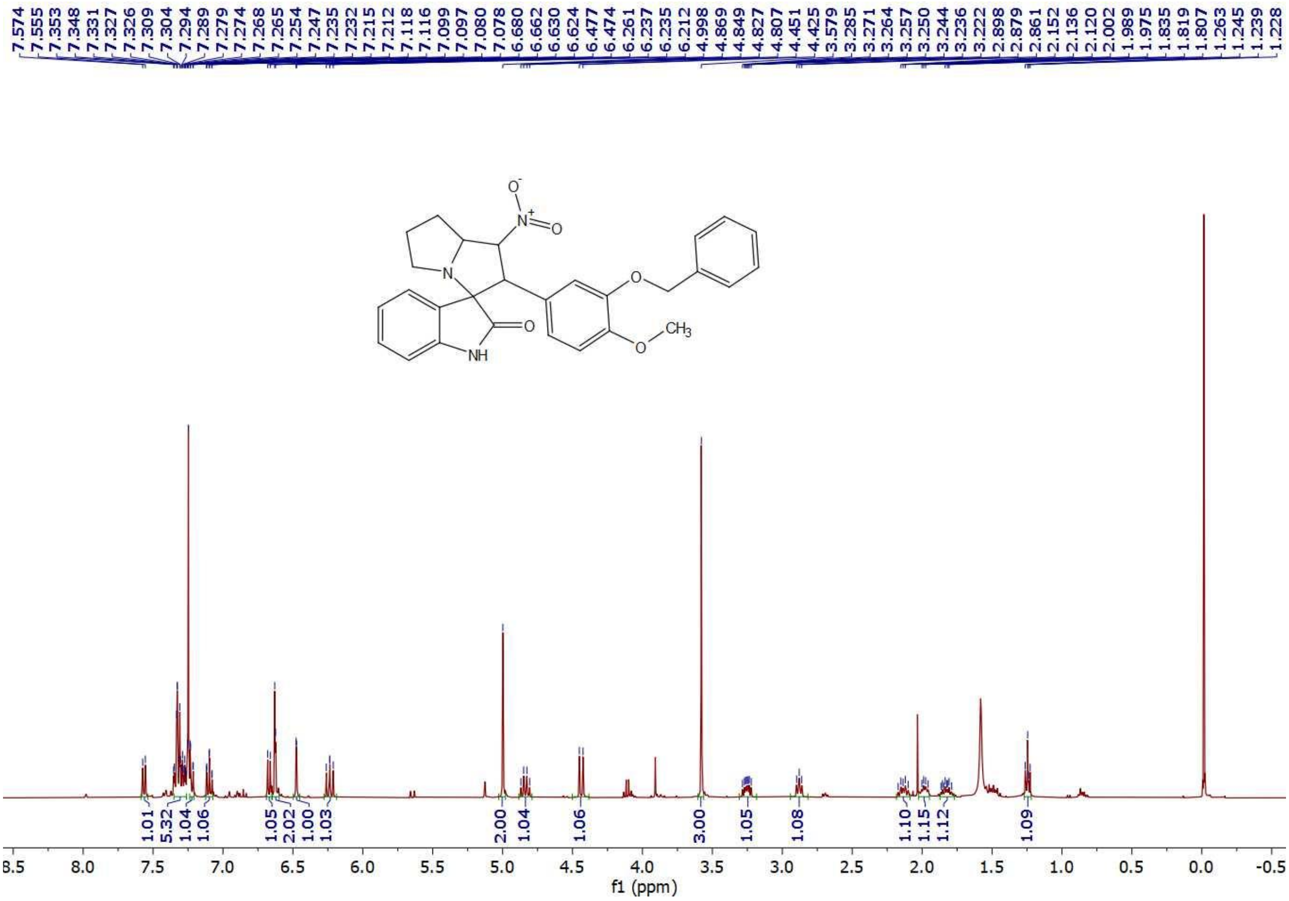

^{1}H-NMR (400 MHz, chloroform-*d*) spectrum of compound **4p**

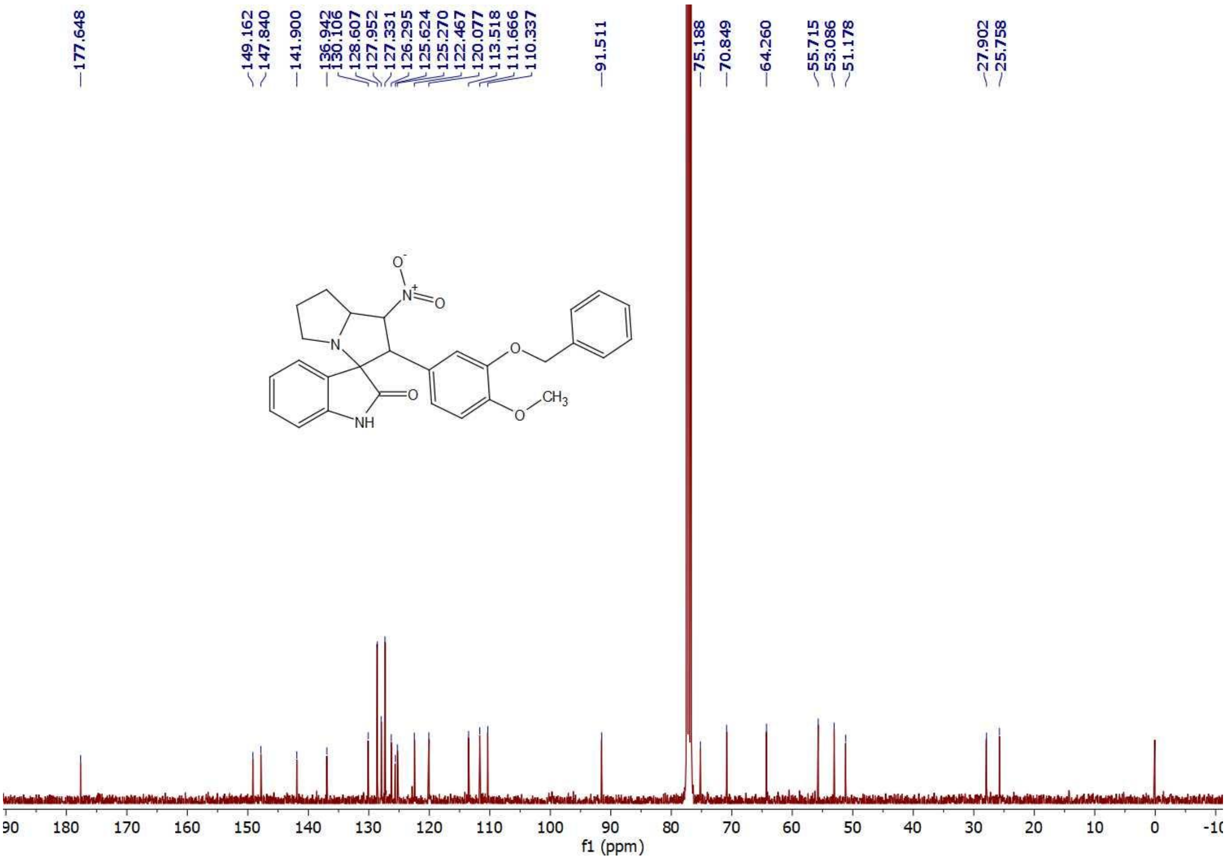

^{13}C-NMR (100 MHz, chloroform-*d*) spectrum of compound **4p**

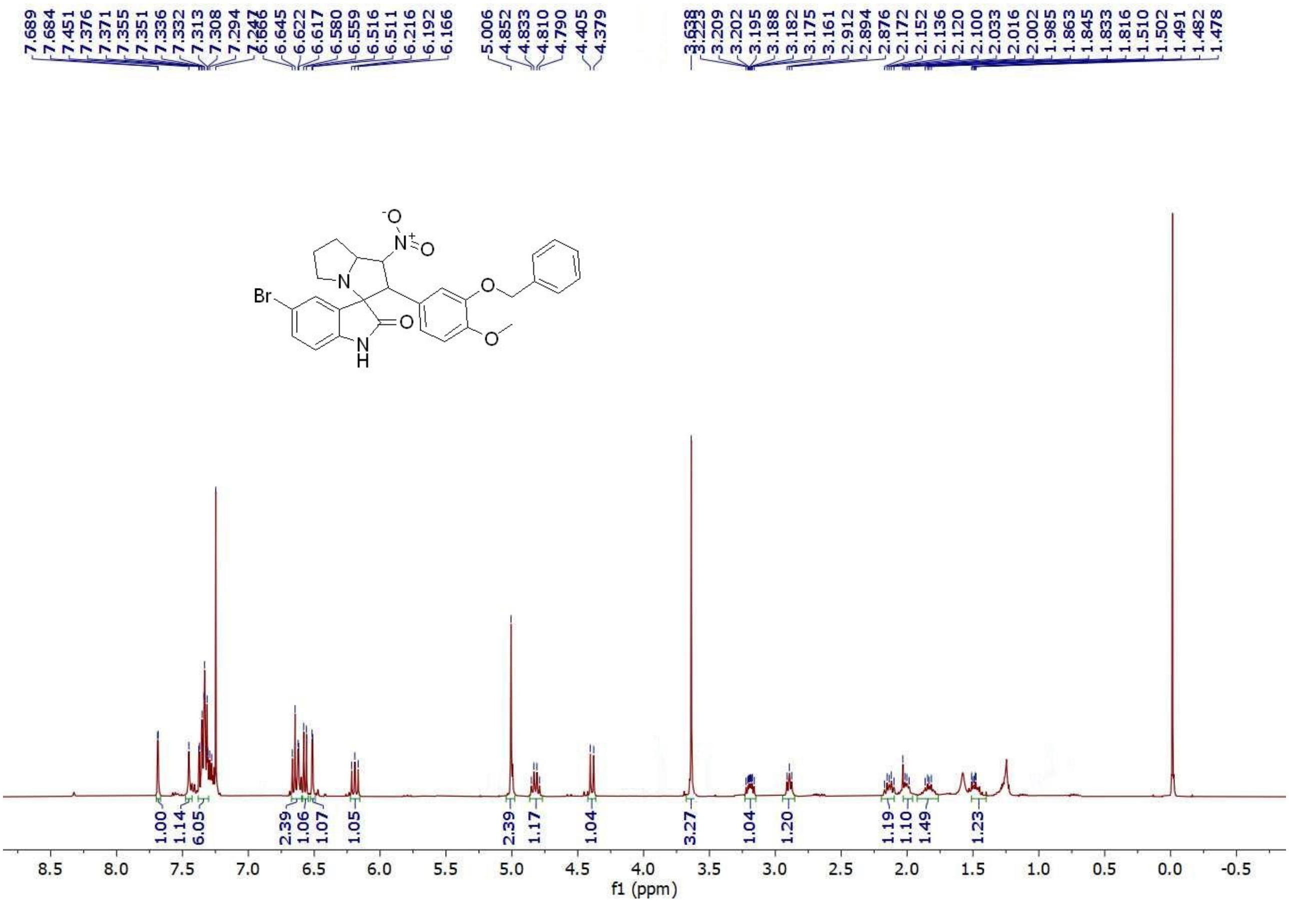

^{1}H-NMR (400 MHz, chloroform-*d*) spectrum of compound **4q**

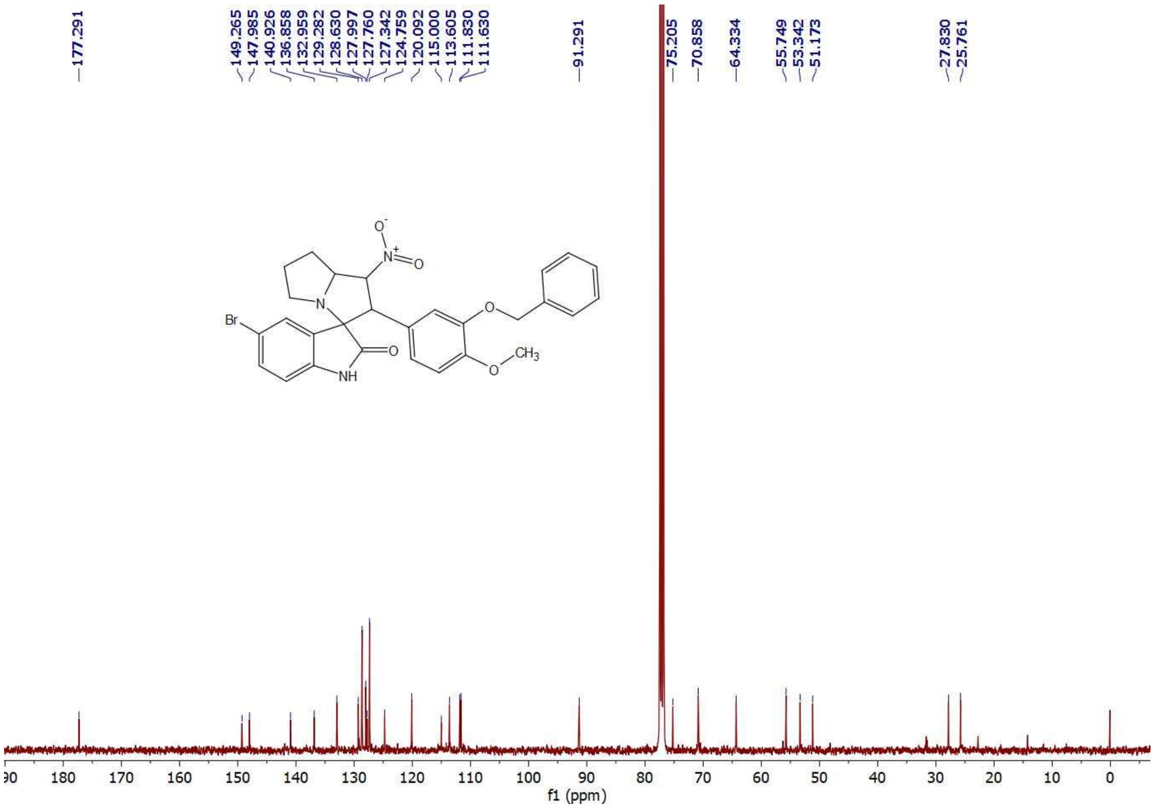

^{13}C-NMR (100 MHz, chloroform-*d*) spectrum of compound **4q**

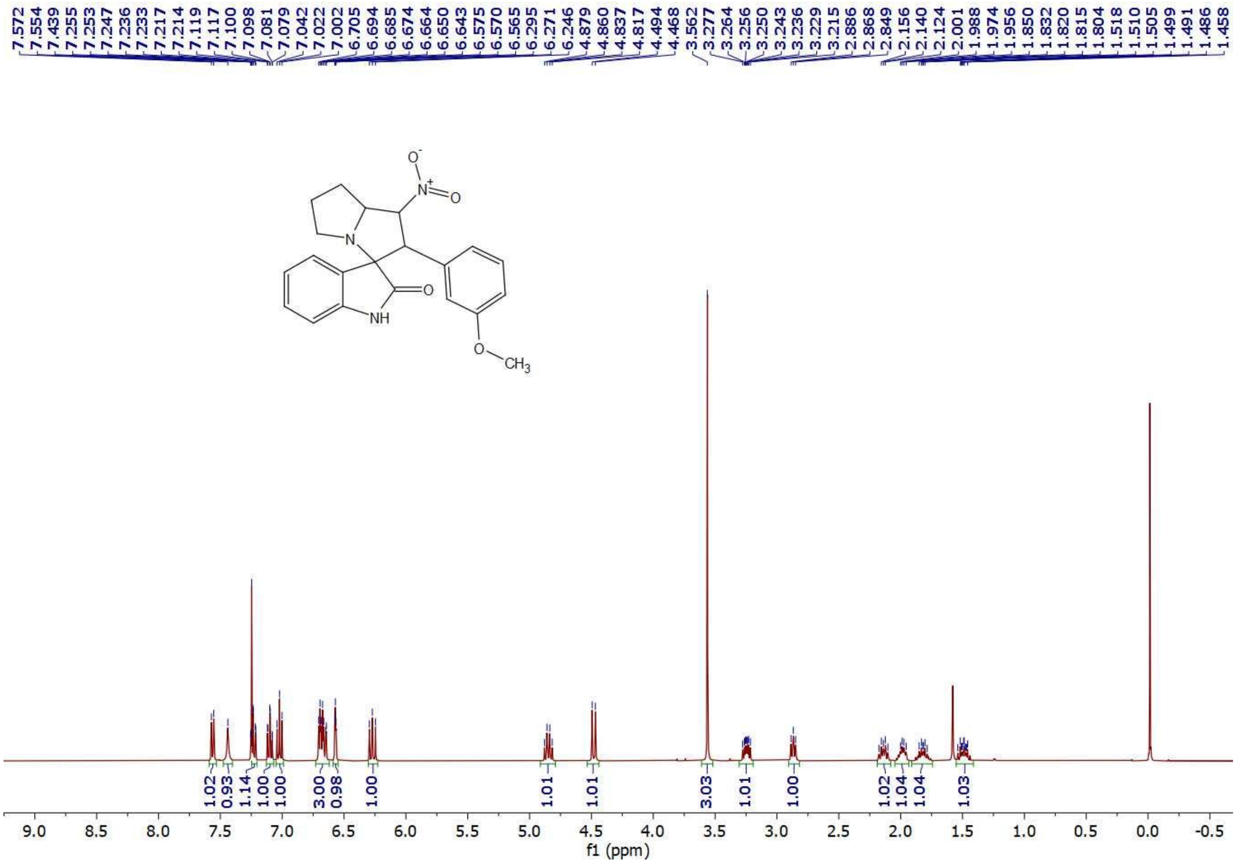

^{1}H-NMR (400 MHz, chloroform-*d*) spectrum of compound **4r**

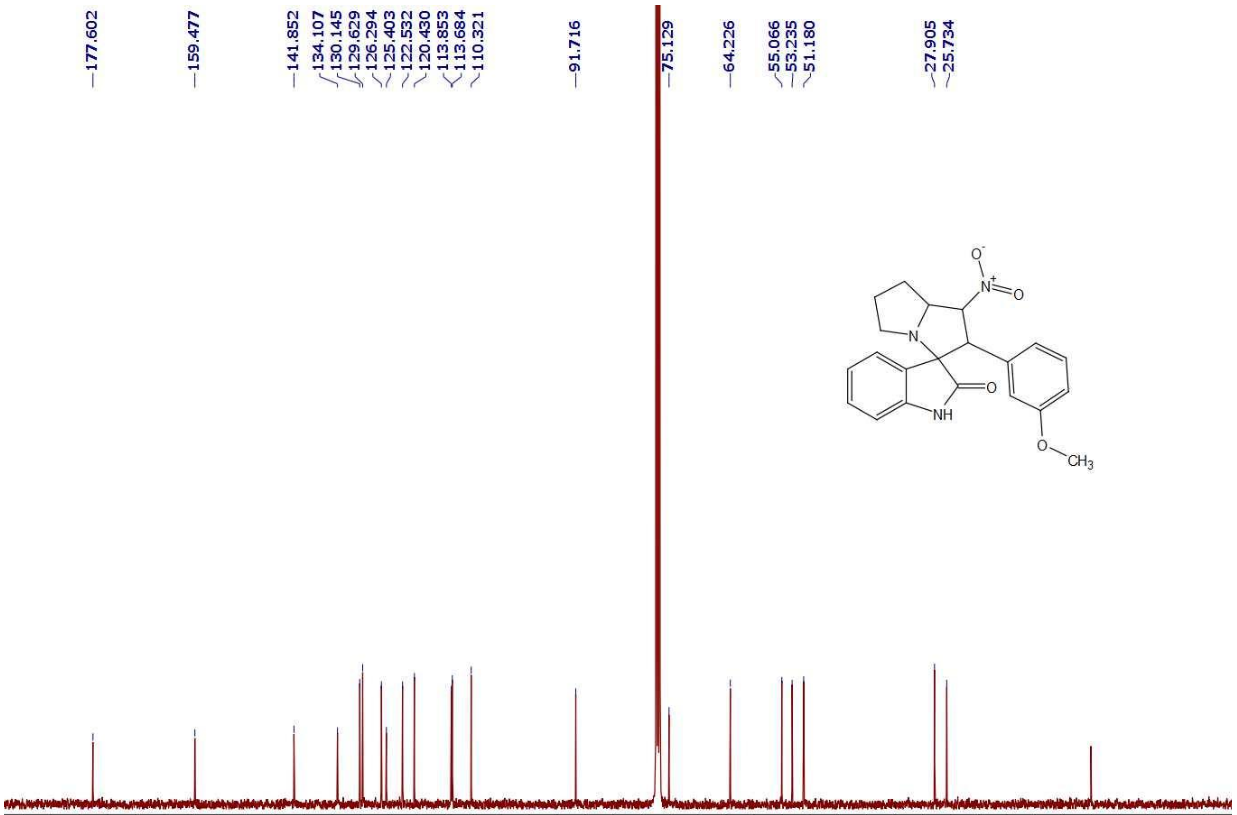

^{13}C-NMR (100 MHz, chloroform-*d*) spectrum of compound **4r**

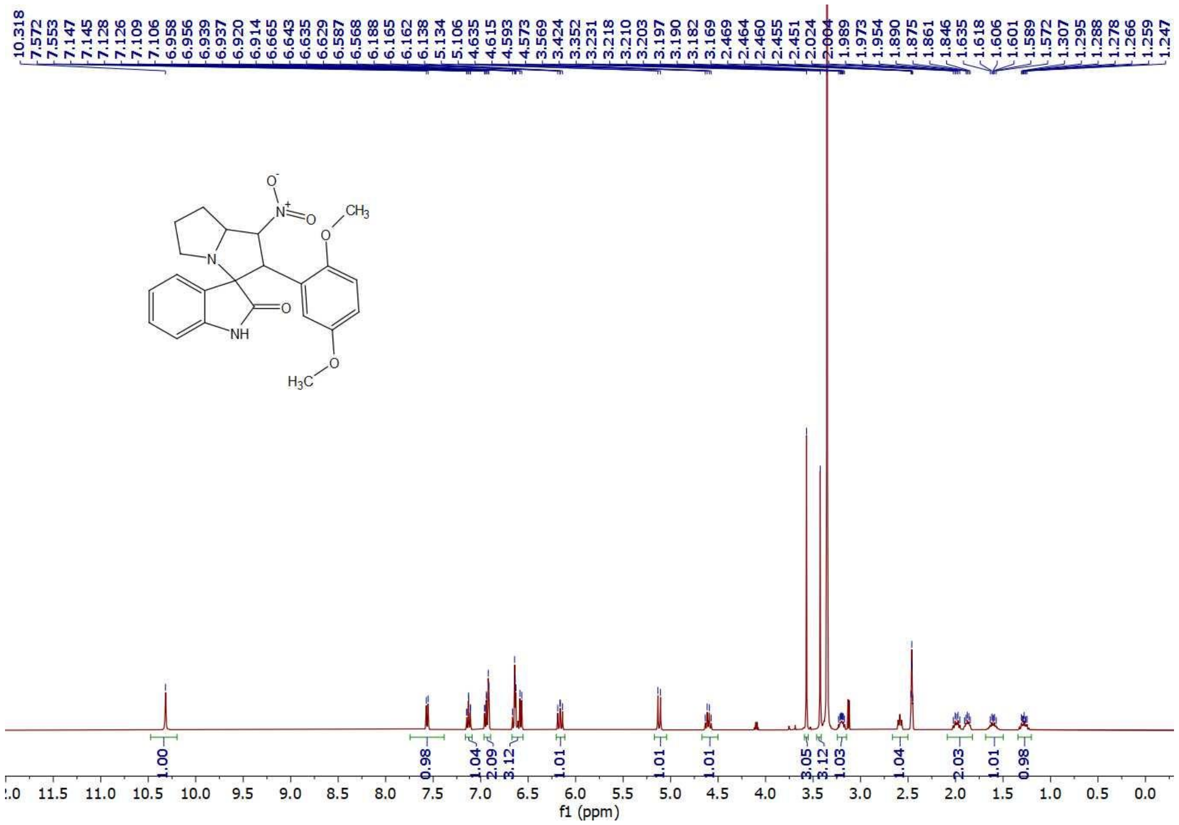

^{1}H-NMR (400 MHz, DMSO-d_6) spectrum of compound **4s**

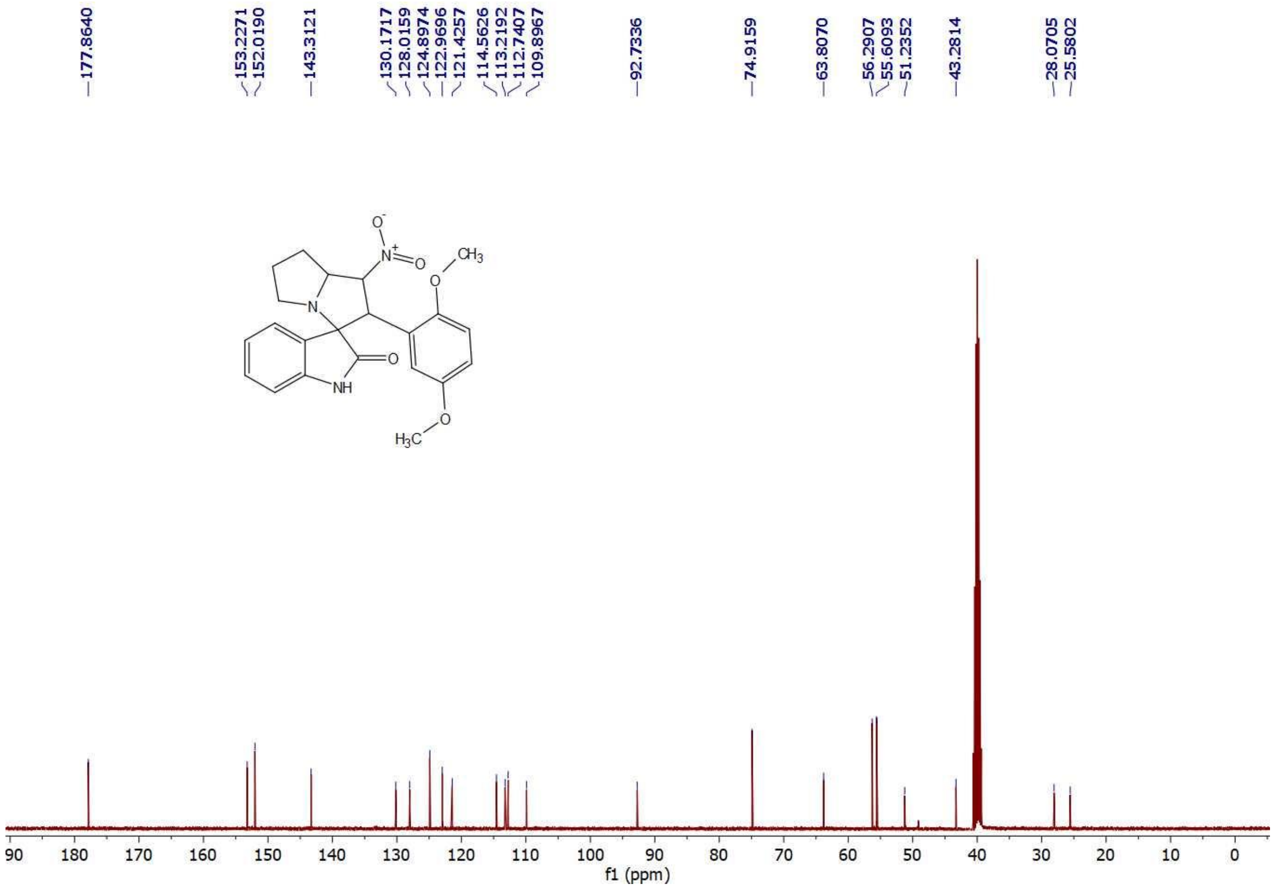

^{13}C-NMR (100 MHz, DMSO-d_6) spectrum of compound **4s**

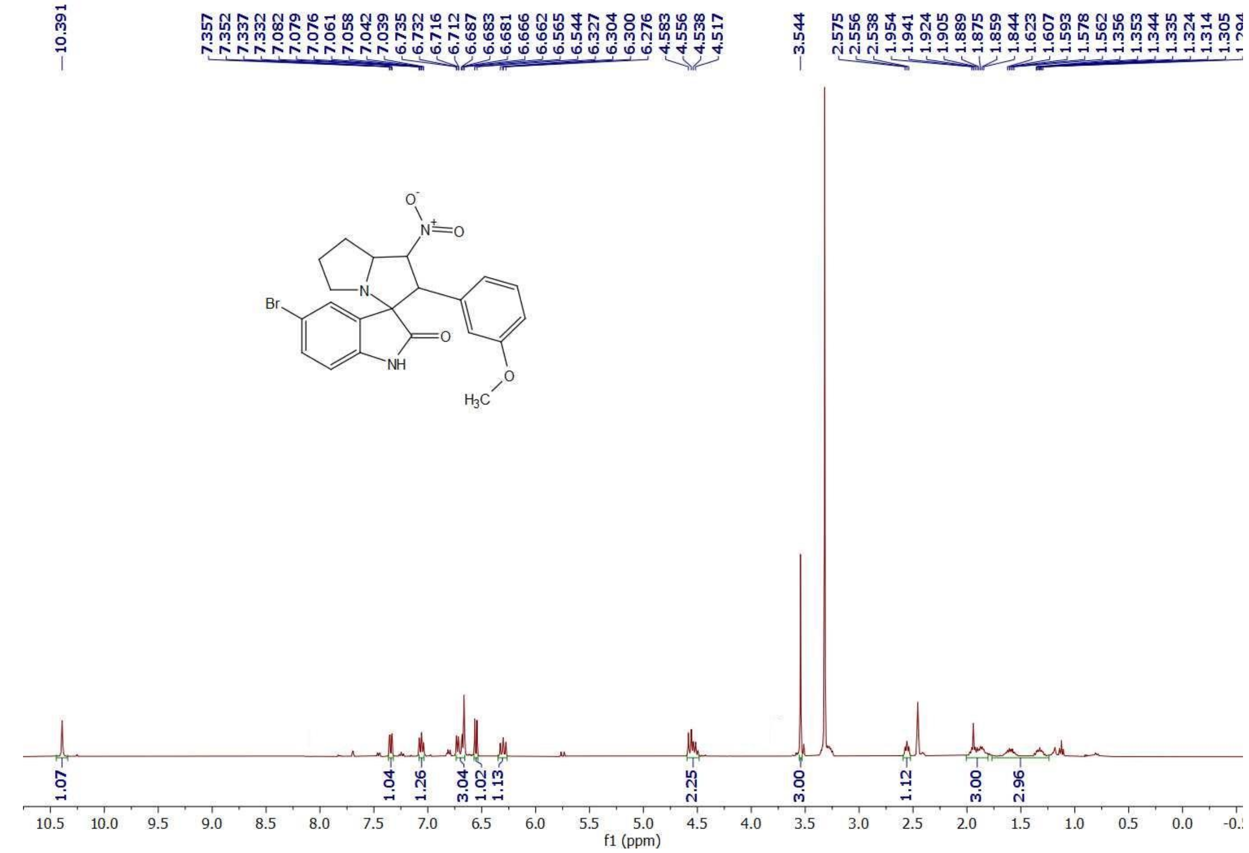

^{1}H-NMR (400 MHz, DMSO-d_6) spectrum of compound **4t**

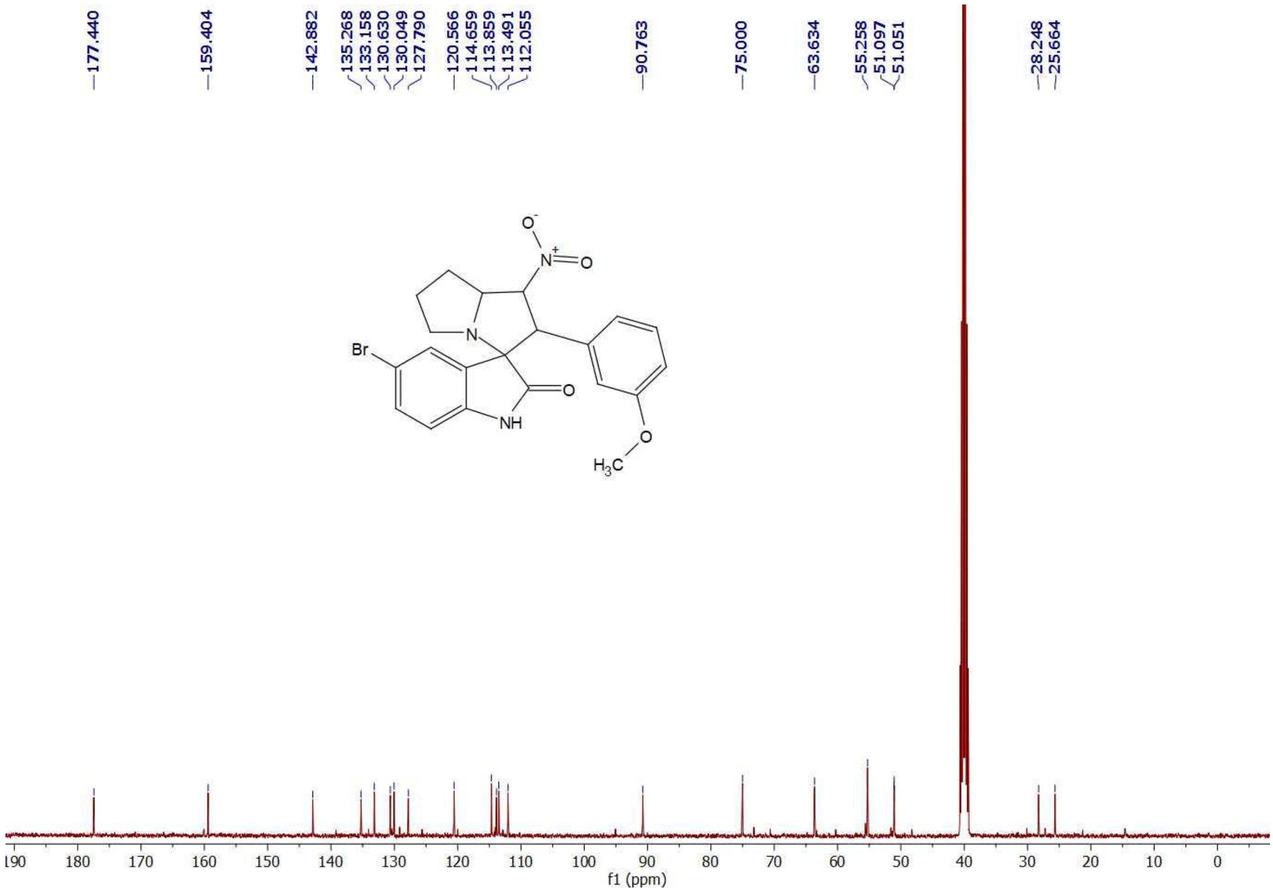

^{13}C-NMR (100 MHz, DMSO-d_6) spectrum of compound **4t**

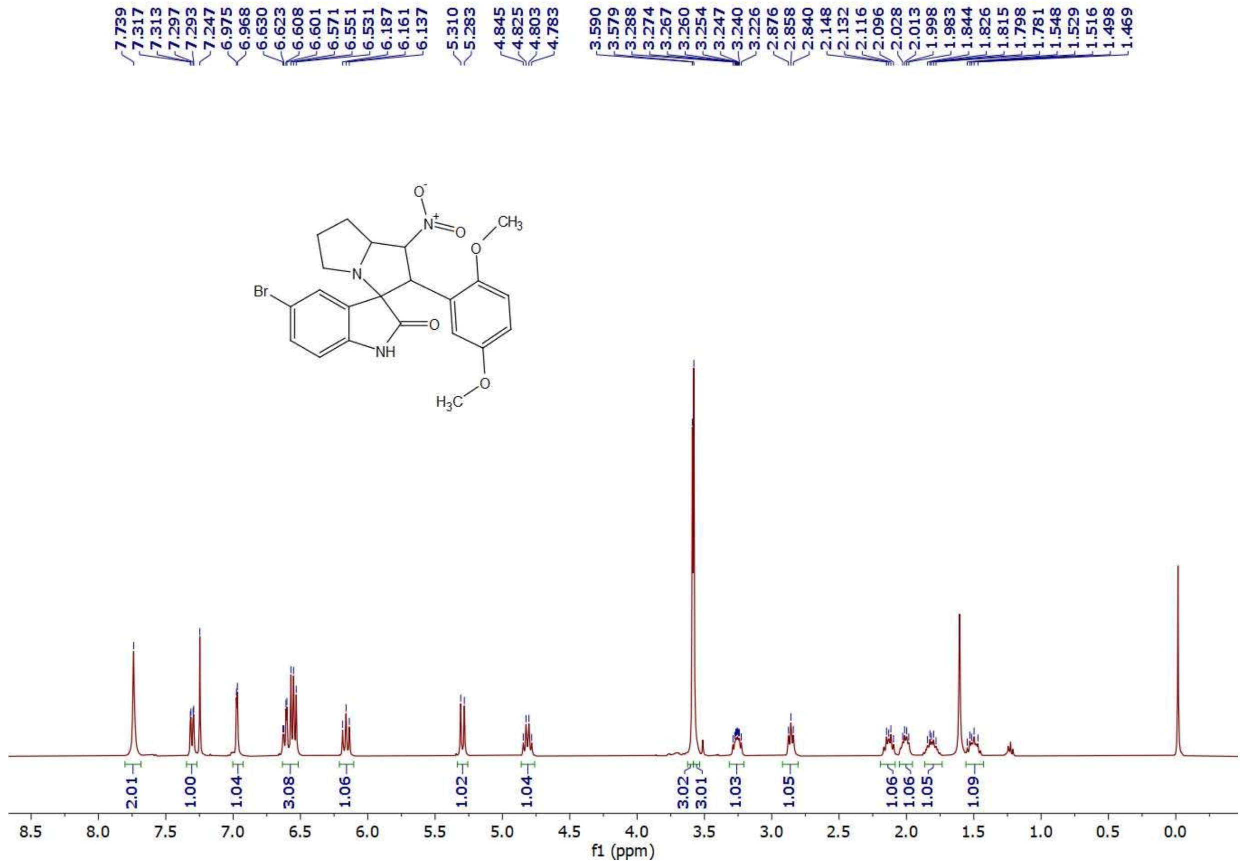

^{1}H-NMR (400 MHz, chloroform-*d*) spectrum of compound **4u**

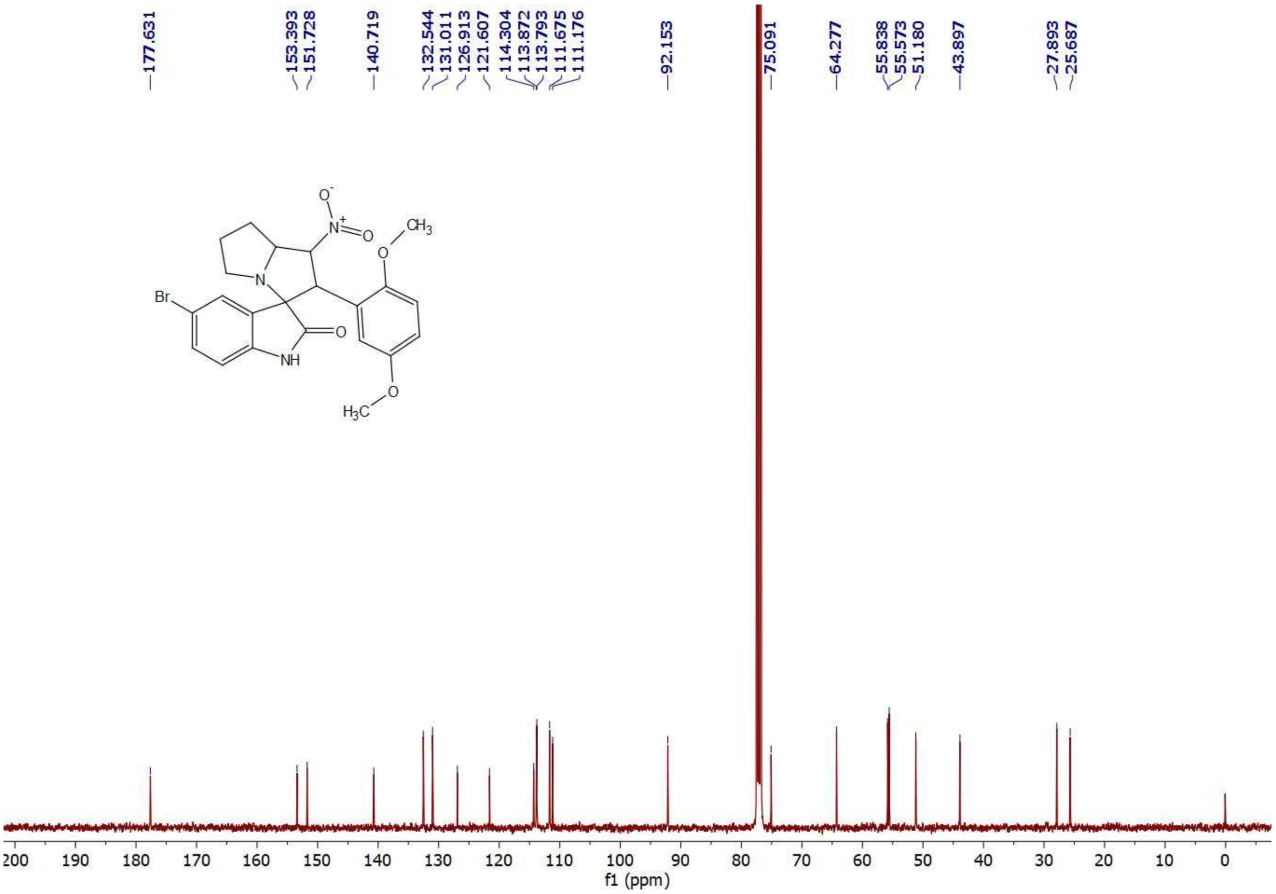

^{13}C-NMR (100 MHz, chloroform-*d*) spectrum of compound **4u**

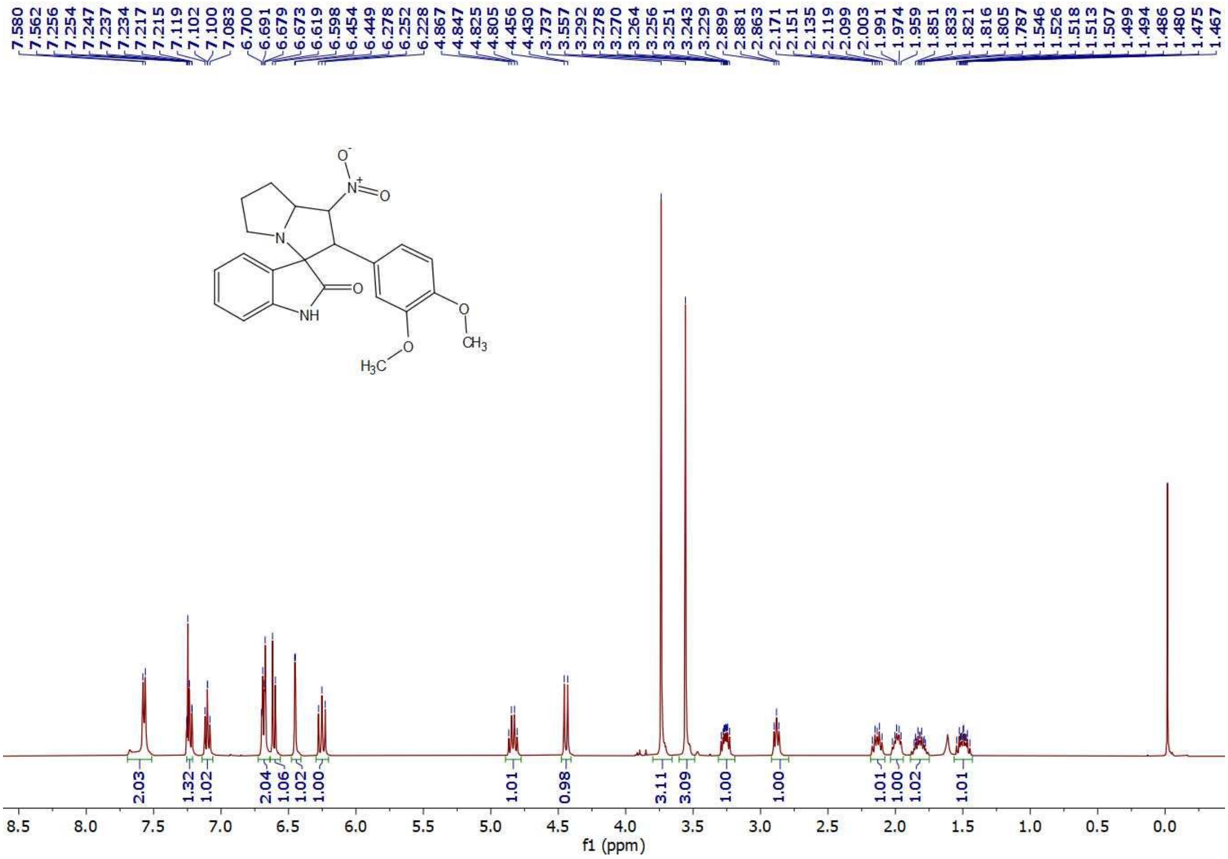

^{1}H-NMR (400 MHz, chloroform-*d*) spectrum of compound **4v**

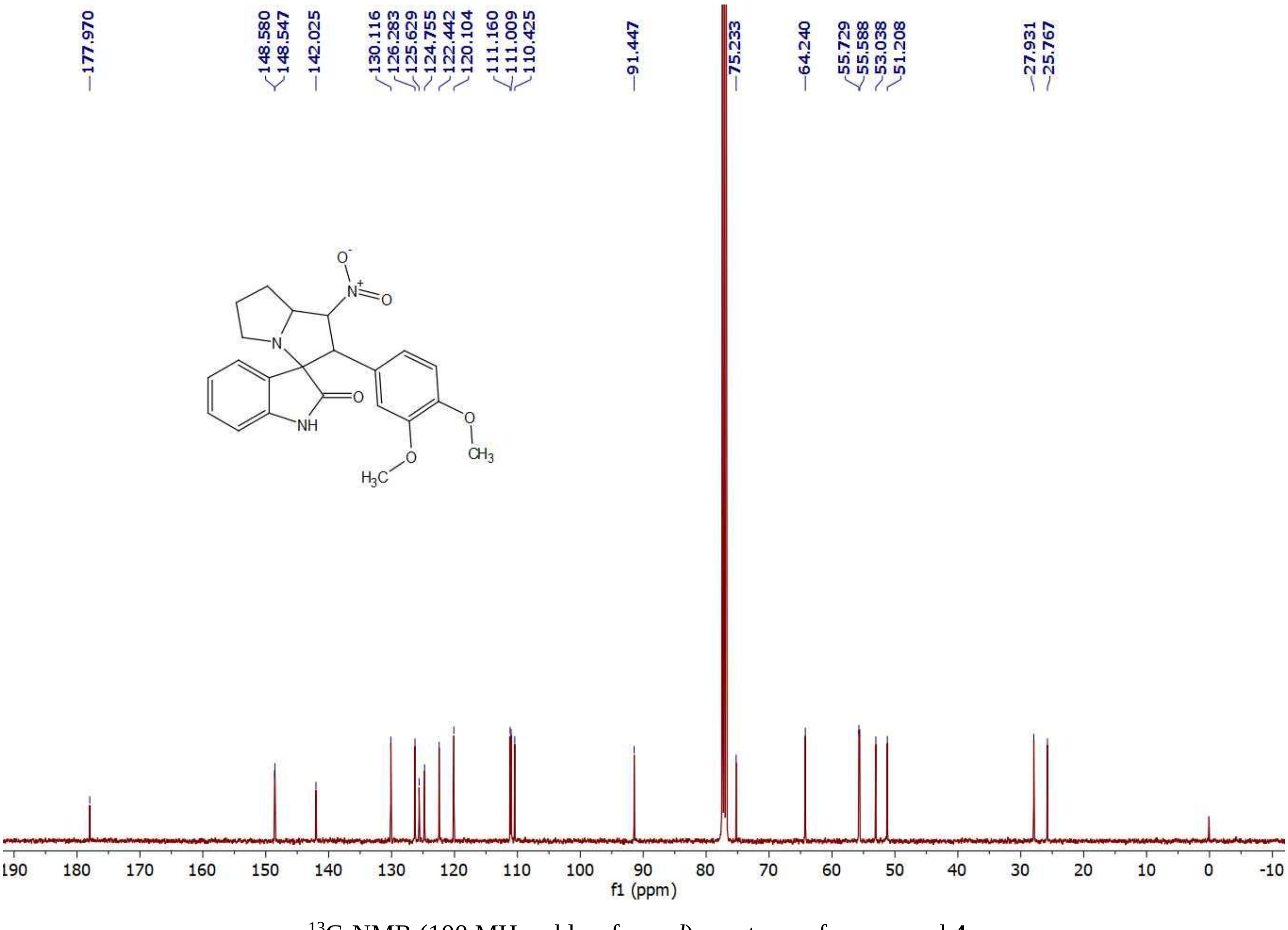

^{13}C-NMR (100 MHz, chloroform-*d*) spectrum of compound **4v**

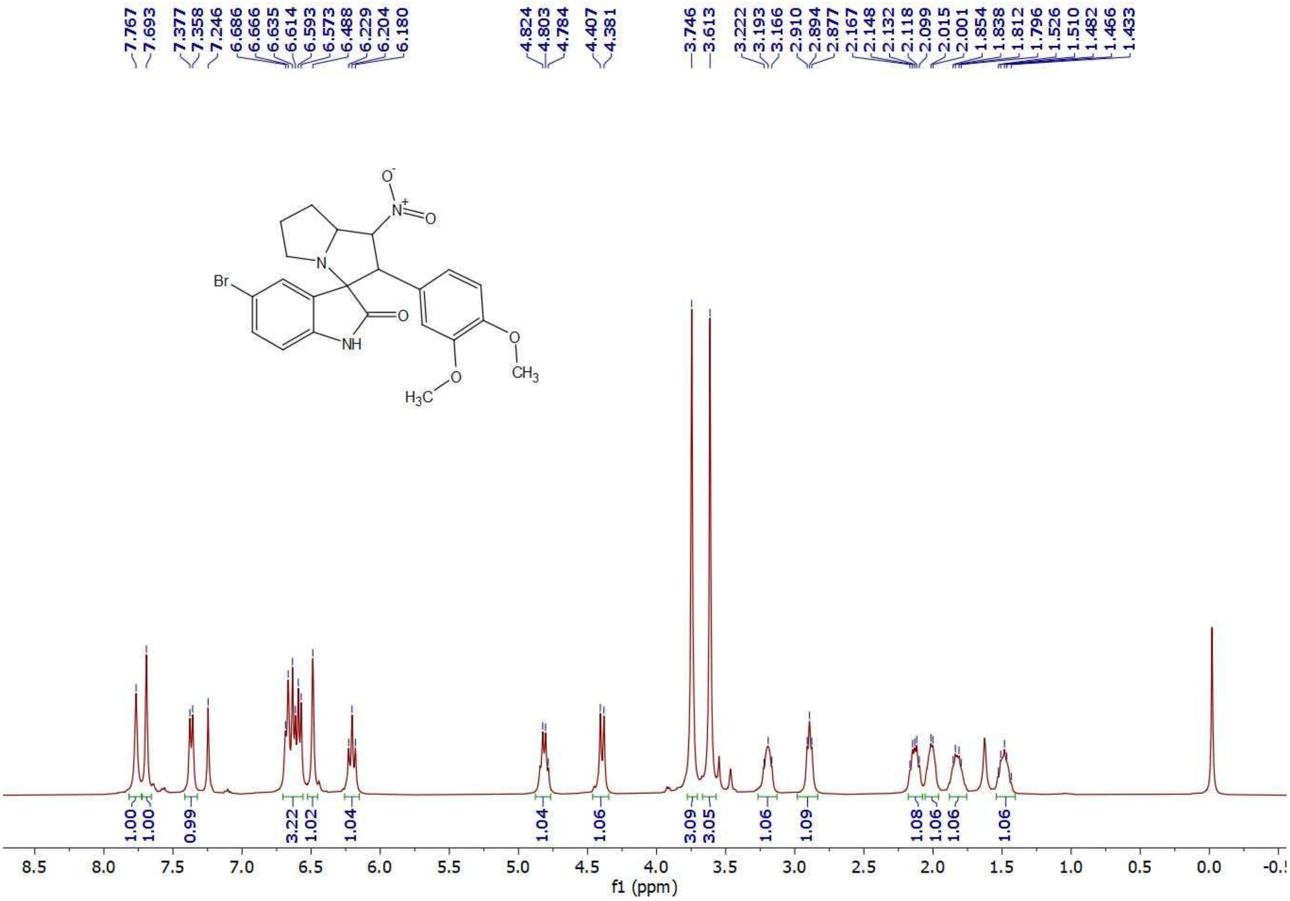

^{1}H-NMR (400 MHz, chloroform-*d*) spectrum of compound **4w**

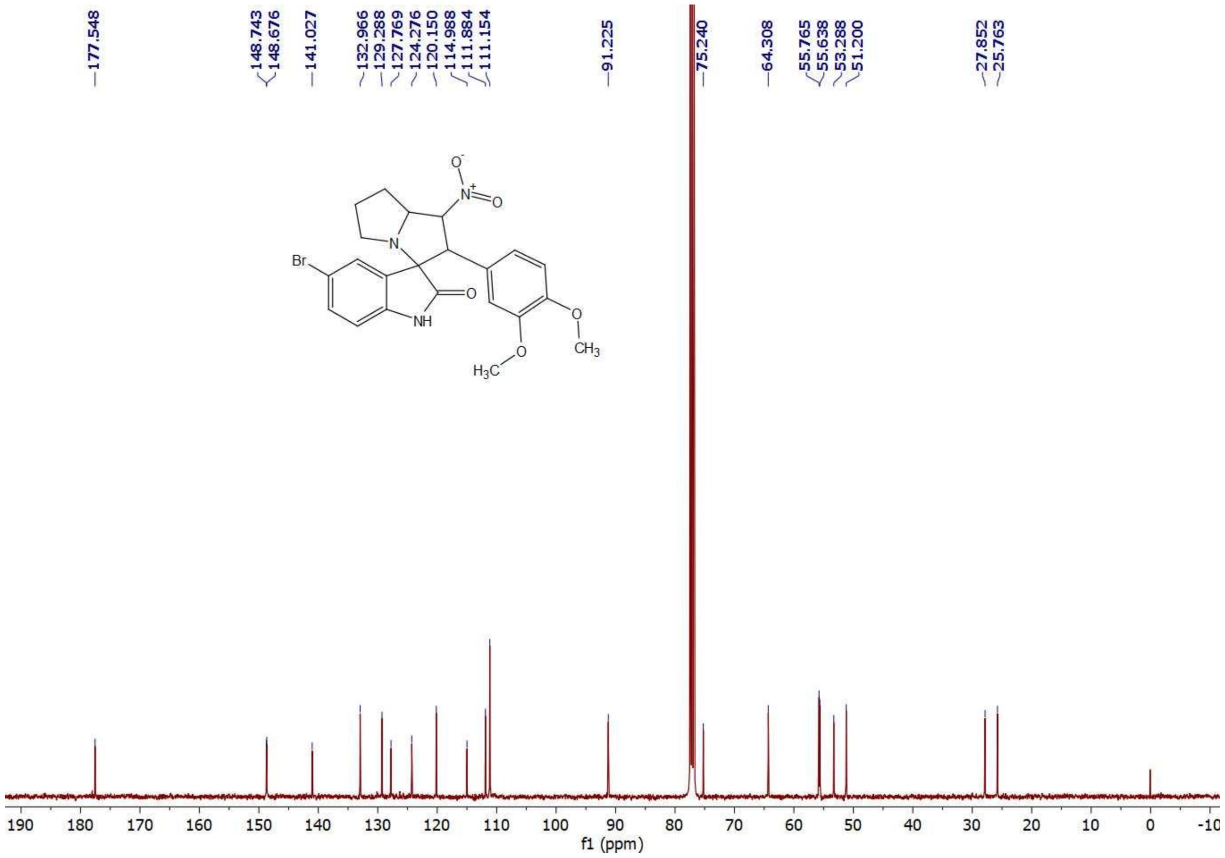

^{13}C-NMR (100 MHz, chloroform-*d*) spectrum of compound **4w**

Mass Spectrum of of Spiro[pyrrolidine-2,3'-oxindoles] 4a-w

User Spectrum Plot Report

Agilent Trusted Answers

Name	SCH-5	Rack Pos.		Instrument	LCMS	Operator	
Inj. Vol. (ul)	2	Plate Pos.		IRM Status	Success		
Data File	SCH-5A.d	Method (Acq)	General Method NW.m	Comment		Acq. Time (Local)	12-08-2023 21:47:46 (UTC+05:30)

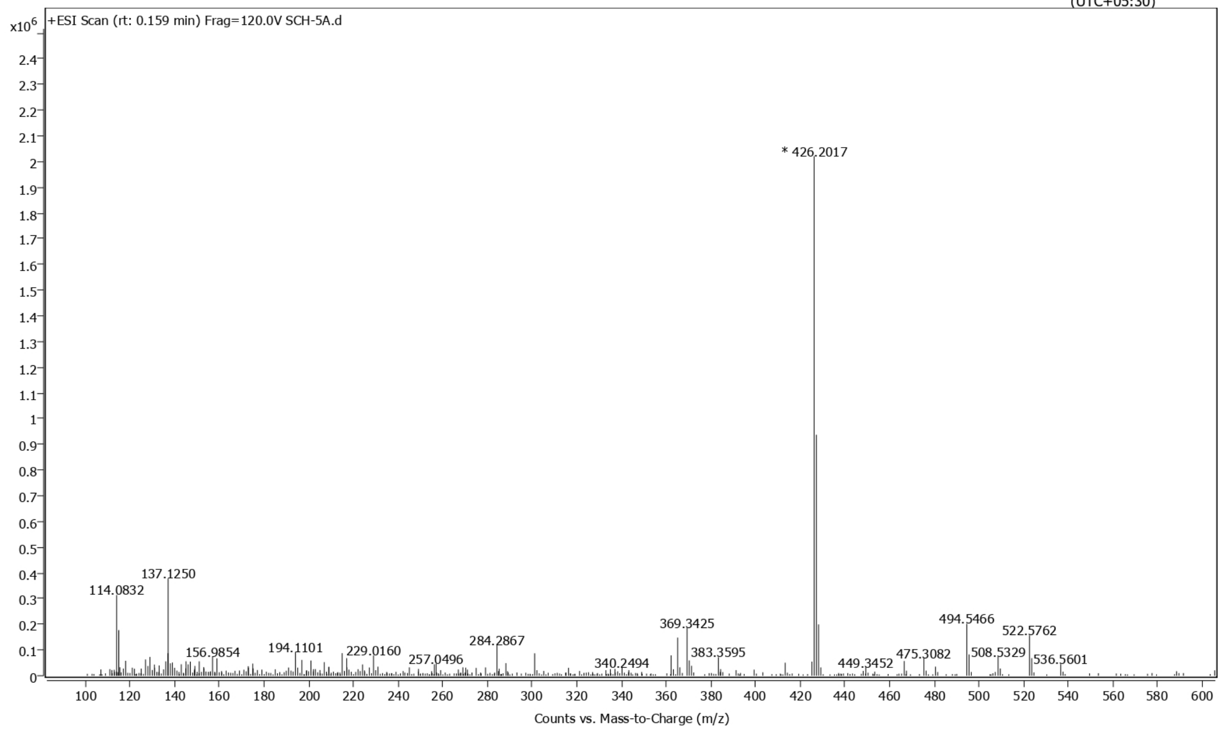

Page 1 of 1 Generated at 07:39 on 13-08-2023

Mass Spectrum of compound 4a

User Spectrum Plot Report

Name	SCH-RS-7	Rack Pos.		Instrument	LCMS	Operator	
Inj. Vol. (ul)	2	Plate Pos.		IRM Status	Success		
Data File	SCH-RS-7.d	Method (Acq)	General Method NW.m	Comment		Acq. Time (Local)	10-08-2023 4.20.08 PM (UTC+05:30)

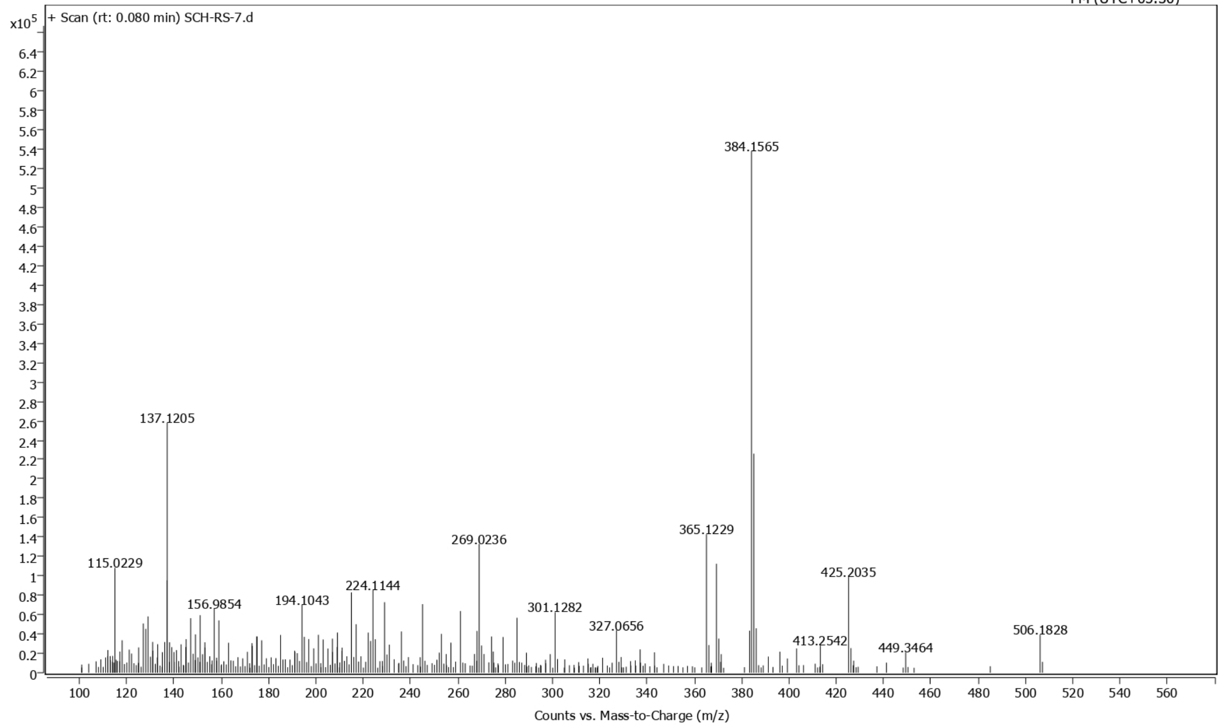

Generated at 03.45 PM on 11-08-2023

Mass Spectrum of compound 4b

User Spectrum Plot Report

Name	SCH-RS-8	Rack Pos.		Instrument	LCMS	Operator	
Inj. Vol. (ul)	2	Plate Pos.		IRM Status	Success		
Data File	SCH-RS-8.d	Method (Acq)	General Method NW.m	Comment		Acq. Time (Local)	10-08-2023 4.22.03 PM (UTC+05:30)

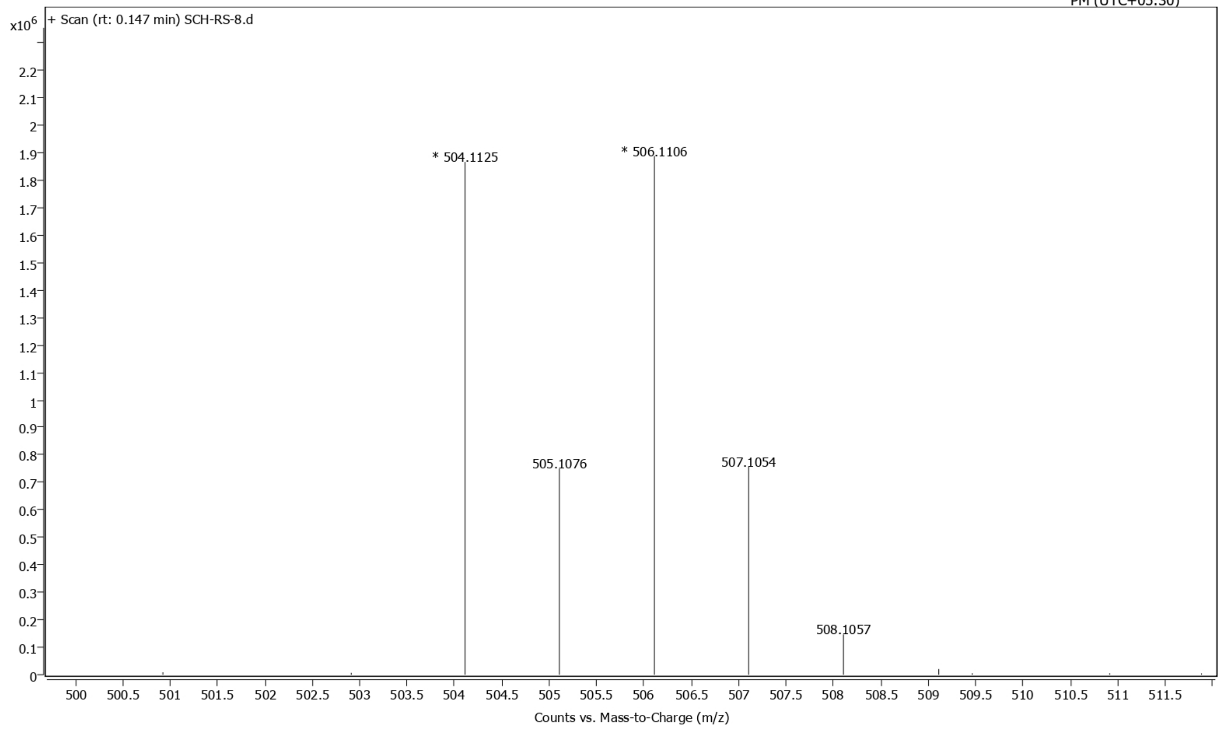

Page 1 of 1 Generated at 06.26 PM on 10-08-2023

Mass Spectrum of compound 4c

User Spectrum Plot Report

Name	SCH-05-86	Rack Pos.		Instrument	LCMS	Operator	
Inj. Vol. (ul)	2	Plate Pos.		IRM Status	Success		
Data File	SCH-05-86.d	Method (Acq)	General Method NW.m	Comment		Acq. Time (Local)	10-08-2023 3.47.26 PM (UTC+05:30)

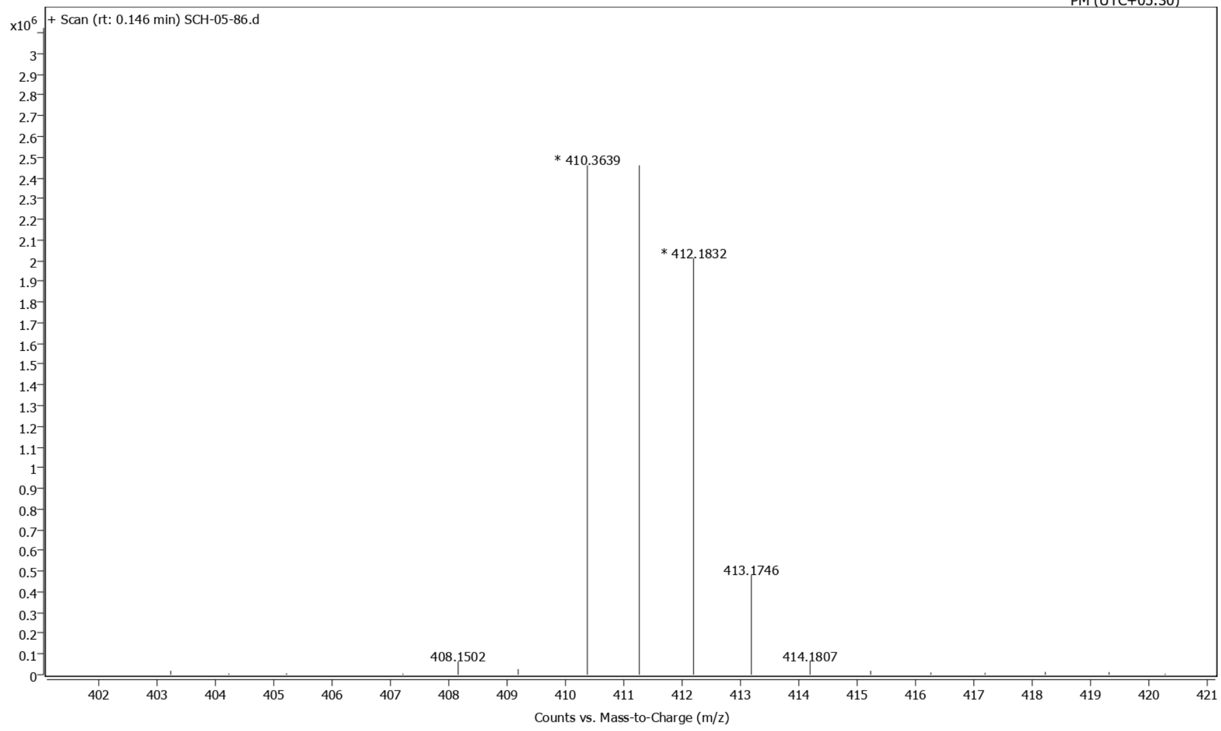

Page 1 of 1 Generated at 11.33 AM on 13-08-2023

Mass Spectrum of 4d

User Spectrum Plot Report

Agilent Trusted Answers

Name	SCH-RS-10	Rack Pos.		Instrument	LCMS	Operator	
Inj. Vol. (ul)	2	Plate Pos.		IRM Status	Success		
Data File	SCH-RS-10.d	Method (Acq)	General Method NW.m	Comment		Acq. Time (Local)	10-08-2023 4.27.49 PM (UTC+05:30)

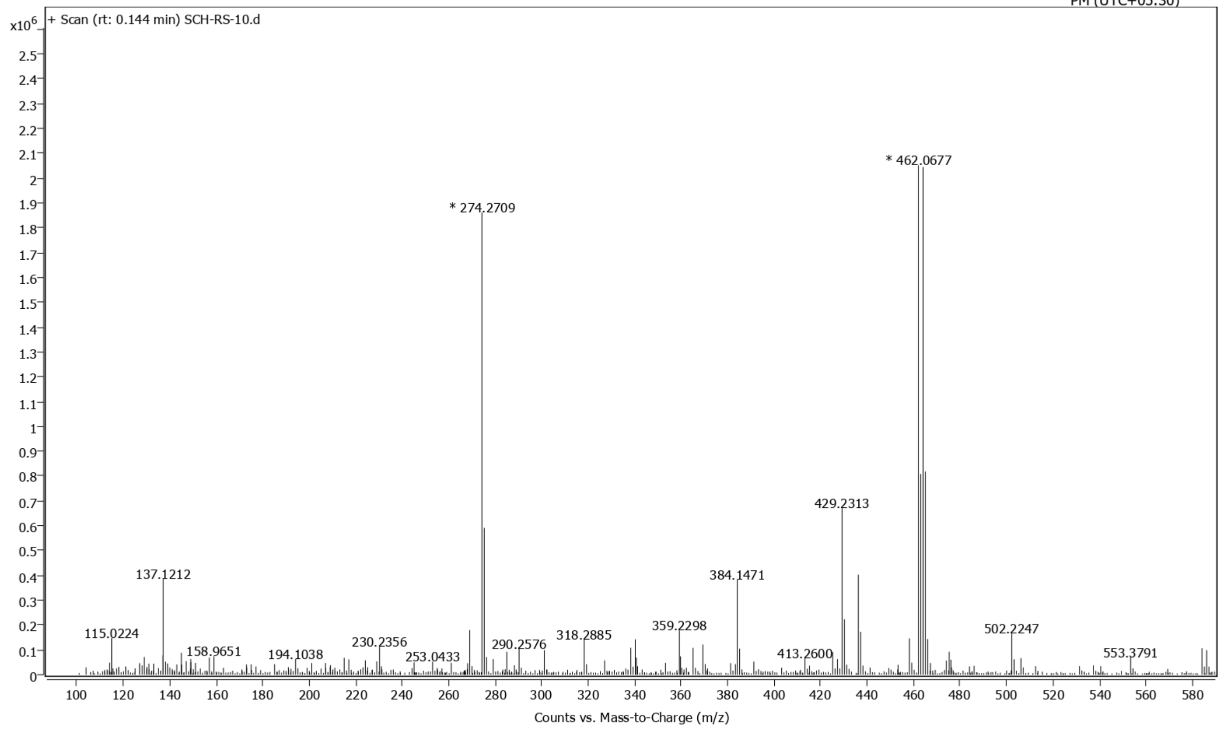

Page 1 of 1 Generated at 07.56 PM on 10-08-2023

Mass Spectrum of 4e

User Spectrum Plot Report

Name	SCH-11	Rack Pos.		Instrument	LCMS	Operator	
Inj. Vol. (ul)	2	Plate Pos.		IRM Status	Success		
Data File	SCH-11.d	Method (Acq)	General Method NW.m	Comment		Acq. Time (Local)	12-08-2023 21:08:55 (UTC+05:30)

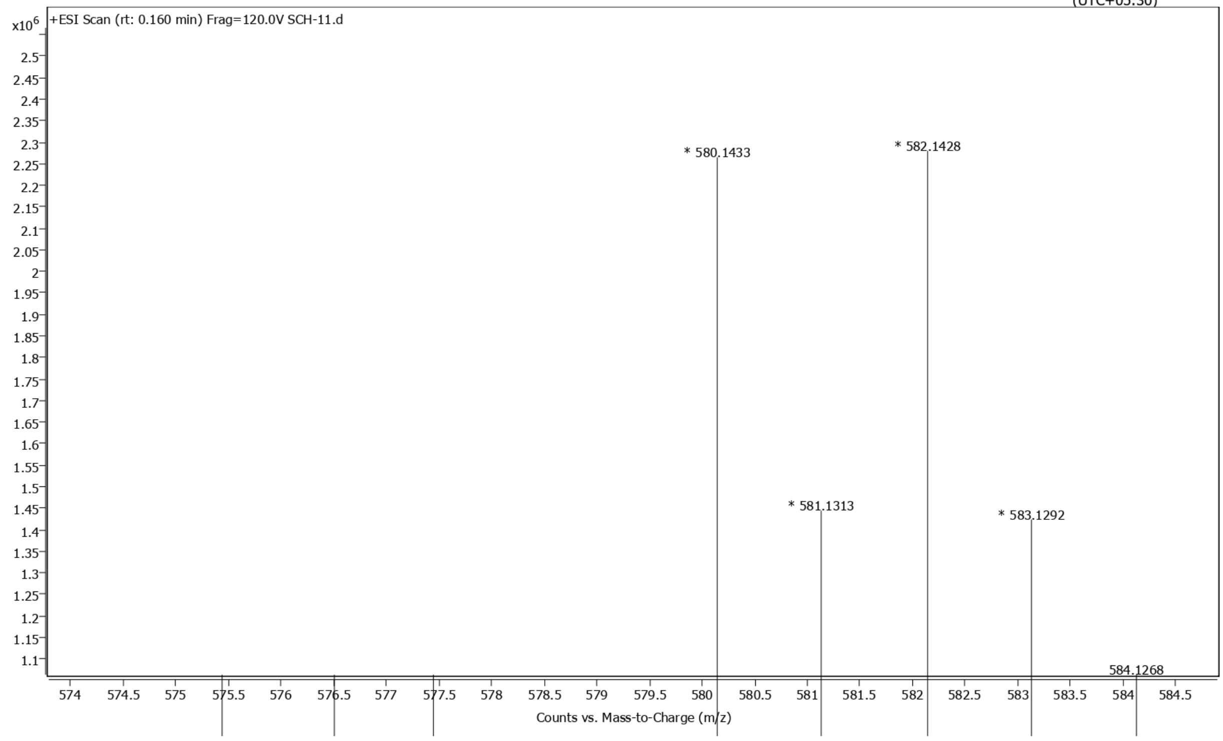

Page 1 of 1 Generated at 07:47 on 13-08-2023

Mass Spectrum of 4f

User Spectrum Plot Report

Name	SCH-13	Rack Pos.		Instrument	LCMS	Operator	
Inj. Vol. (ul)	2	Plate Pos.		IRM Status	Success		
Data File	SCH-13G.d	Method (Acq)	General Method NW.m	Comment		Acq. Time (Local)	13-08-2023 09:26:32 (UTC+05:30)

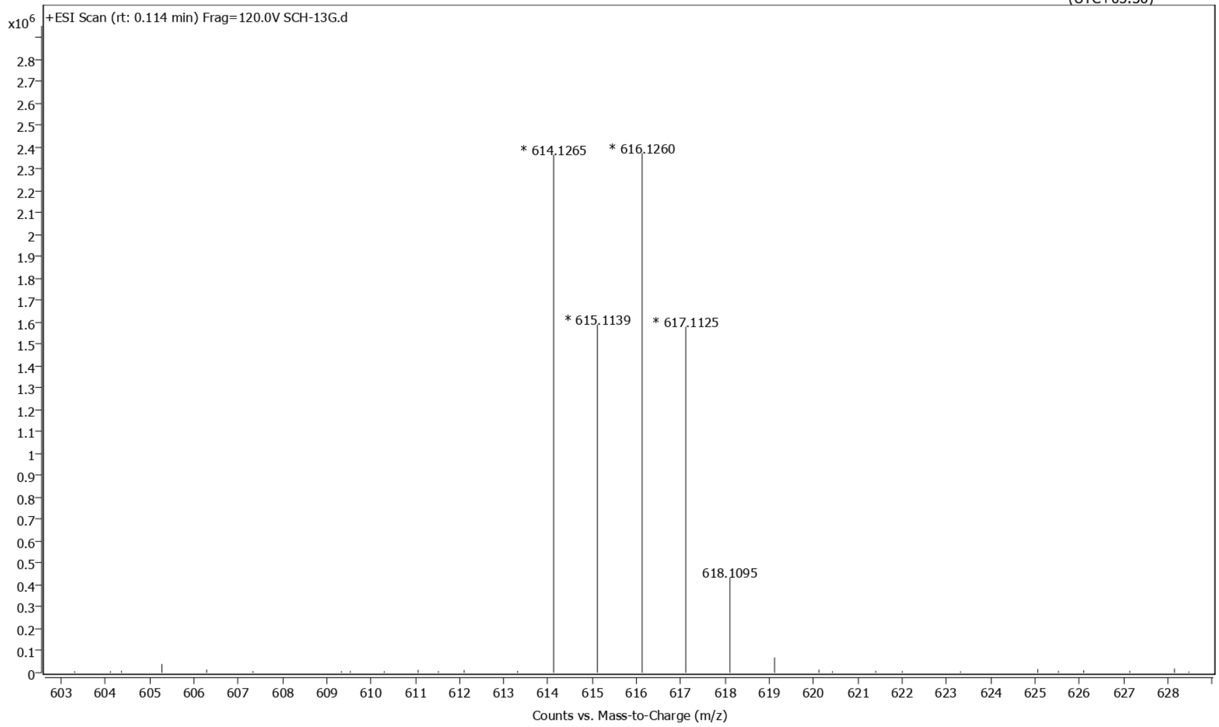

Page 1 of 1

Generated at 10:20 on 13-08-2023

Mass Spectrum of 4g

User Spectrum Plot Report

Name	SCH-RS-16	Rack Pos.		Instrument	LCMS	Operator	
Inj. Vol. (ul)	2	Plate Pos.		IRM Status	Success		
Data File	SCH-RS-16.d	Method (Acq)	General Method NW.m	Comment		Acq. Time (Local)	10-08-2023 3.51.16 PM (UTC+05:30)

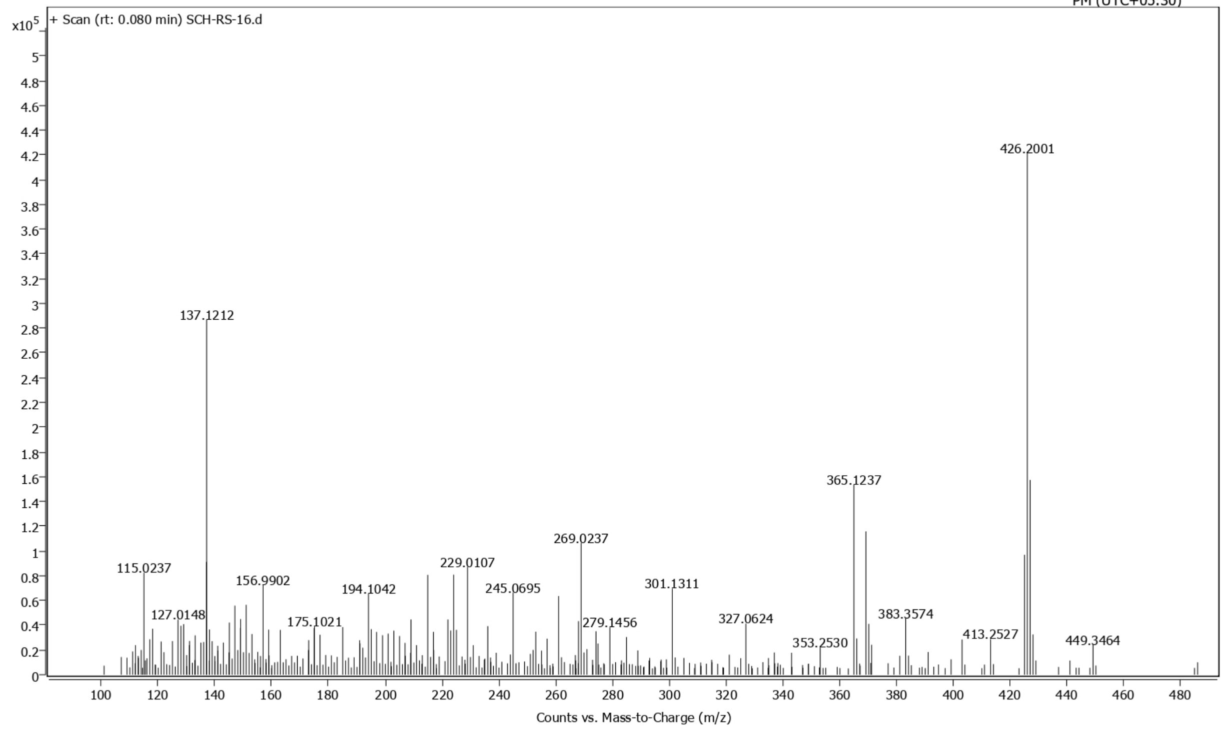

Page 1 of 1 Generated at 07.23 PM on 10-08-2023

Mass Spectrum of 4h

User Spectrum Plot Report

Name	SCH-17	Rack Pos.		Instrument	LCMS	Operator	
Inj. Vol. (ul)	2	Plate Pos.		IRM Status	Success		
Data File	SCH-17.d	Method (Acq)	General Method NW.m	Comment		Acq. Time (Local)	12-08-2023 21:10:50 (UTC+05:30)

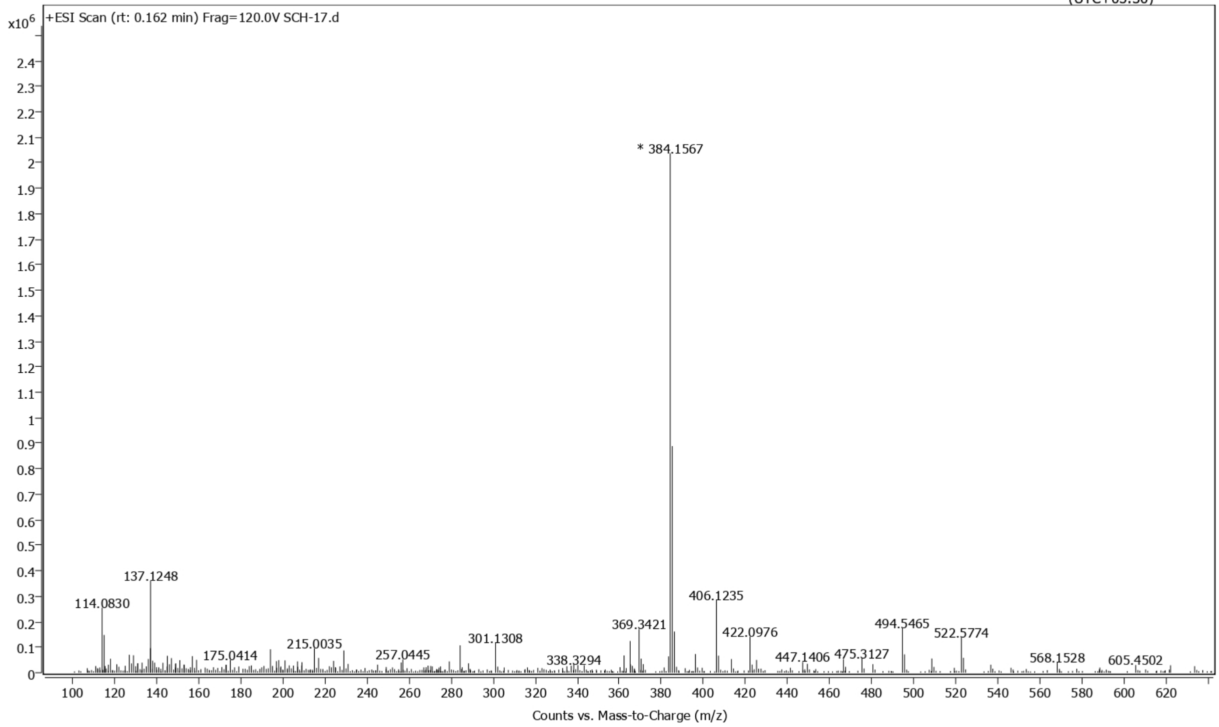

Page 1 of 1 Generated at 07:50 on 13-08-2023

Mass Spectrum of 4i

User Spectrum Plot Report

Name	SCH-RS-18	Rack Pos.		Instrument	LCMS	Operator	
Inj. Vol. (ul)	2	Plate Pos.		IRM Status	Success		
Data File	SCH-RS-18.d	Method (Acq)	General Method NW.m	Comment		Acq. Time (Local)	10-08-2023 4.10.30 PM (UTC+05:30)

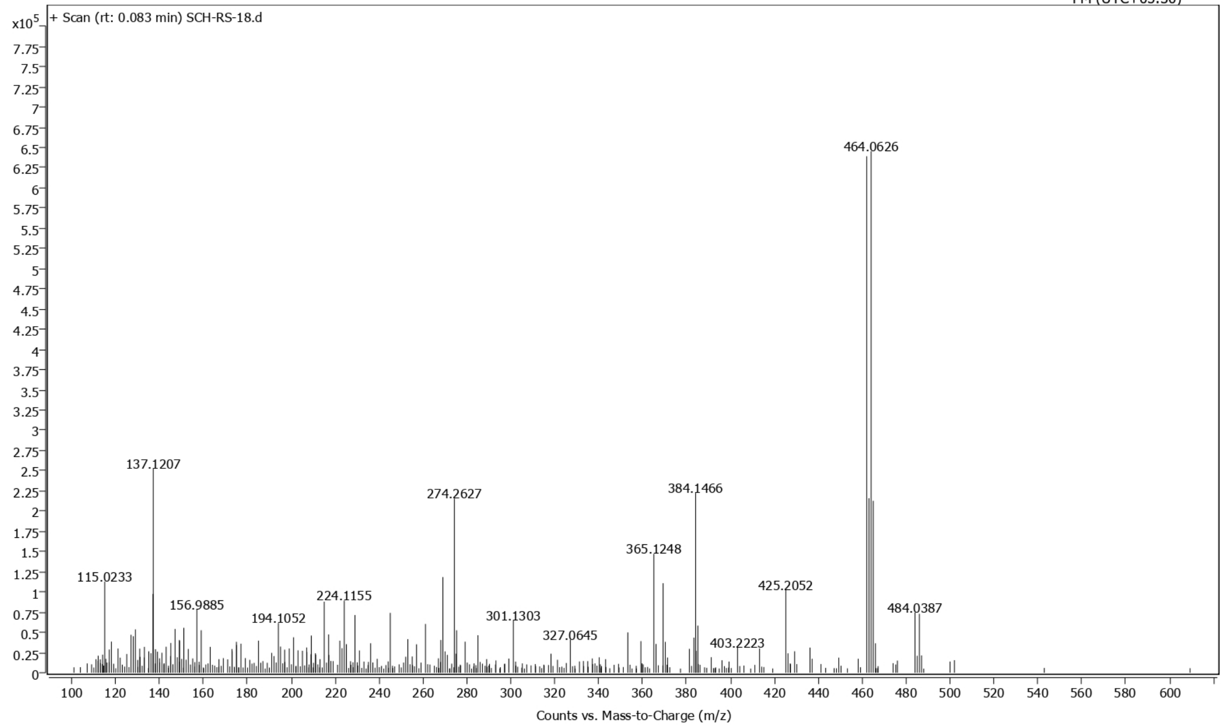

Page 1 of 1 | Generated at 07.46 PM on 10-08-2023

Mass Spectrum of 4j

User Spectrum Plot Report

Agilent | Trusted Answers

Name	SCH-19	Rack Pos.		Instrument	LCMS	Operator	
Inj. Vol. (ul)	2	Plate Pos.		IRM Status	Success		
Data File	SCH-19.d	Method (Acq)	General Method NW.m	Comment		Acq. Time (Local)	12-08-2023 20:57:22 (UTC+05:30)

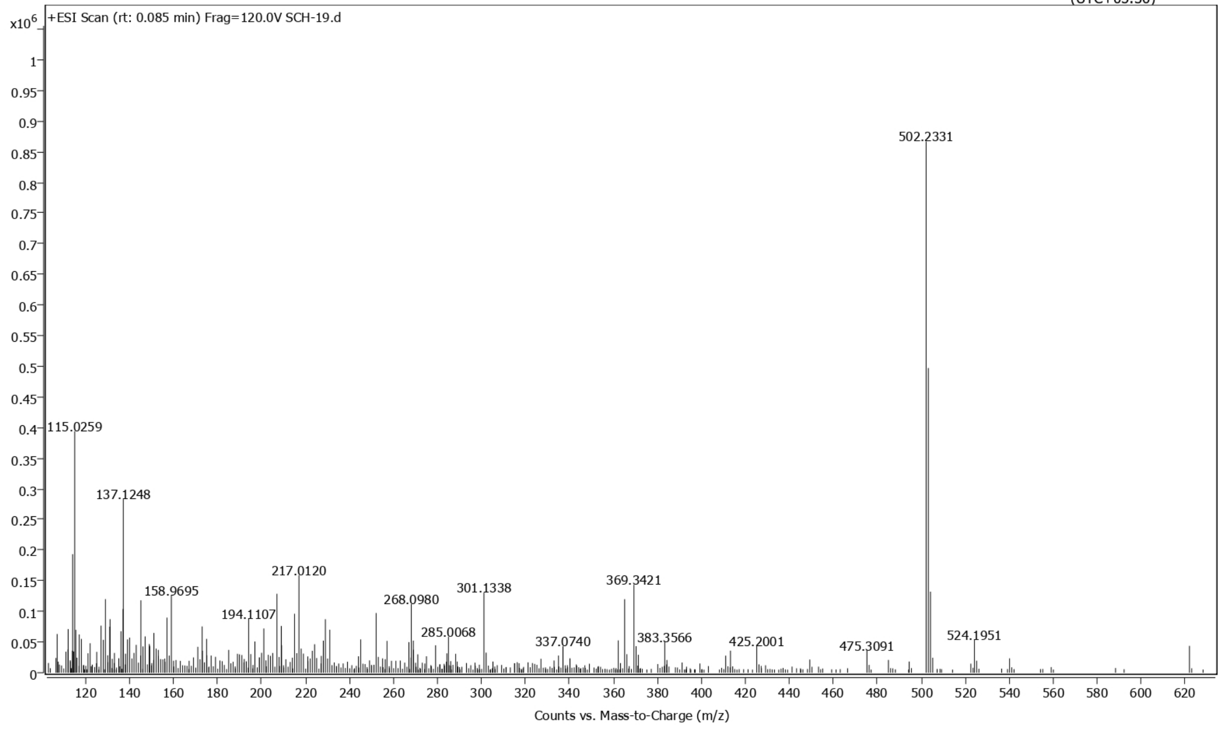

Page 1 of 1

Generated at 10:31 on 13-08-2023

Mass Spectrum of 4k

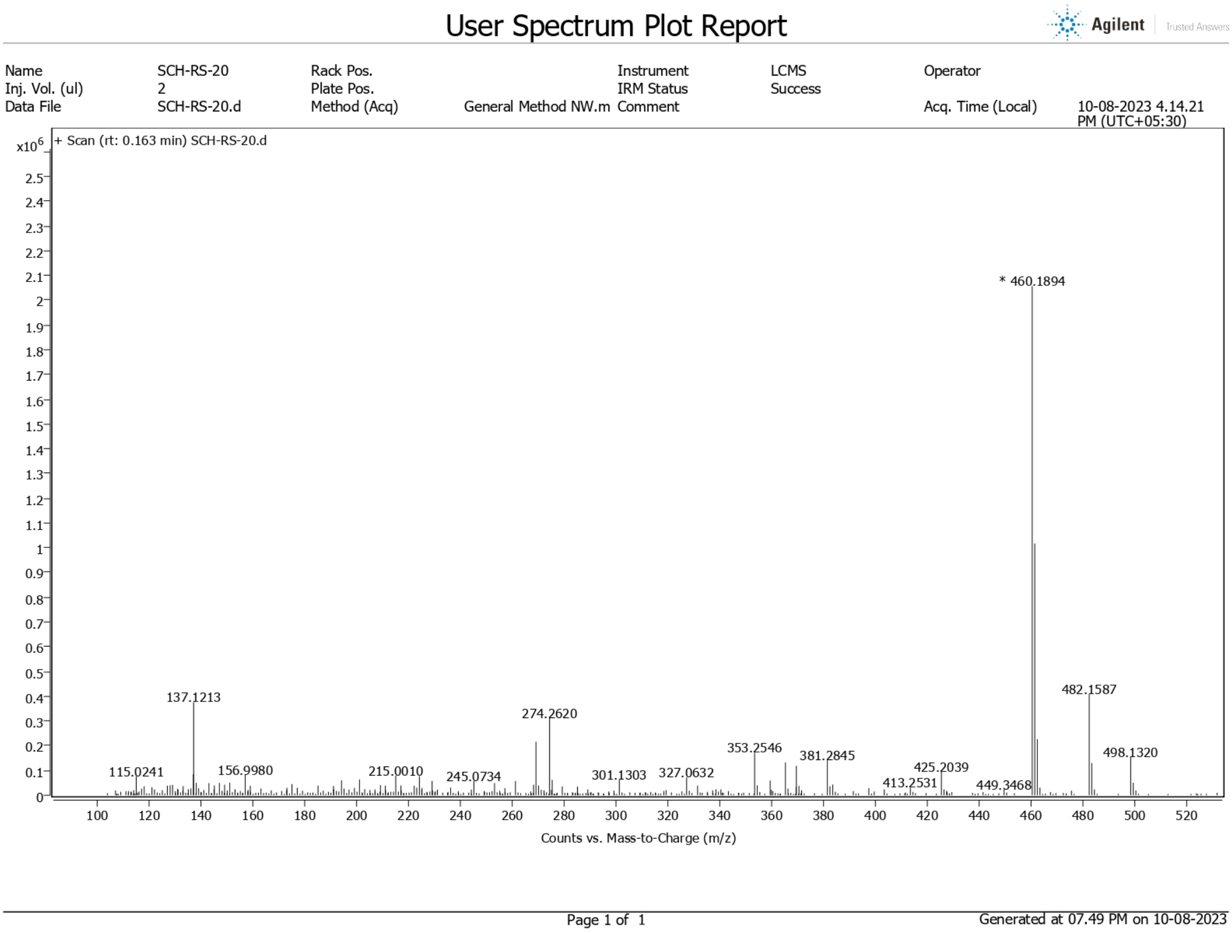

Mass Spectrum of 4l

User Spectrum Plot Report

Agilent Trusted Answers

Name	SCH-RS-21	Rack Pos.		Instrument	LCMS	Operator	
Inj. Vol. (ul)	2	Plate Pos.		IRM Status	Success		
Data File	SCH-RS-21.d	Method (Acq)	General Method NW.m	Comment		Acq. Time (Local)	10-08-2023 4.29.45 PM (UTC+05:30)

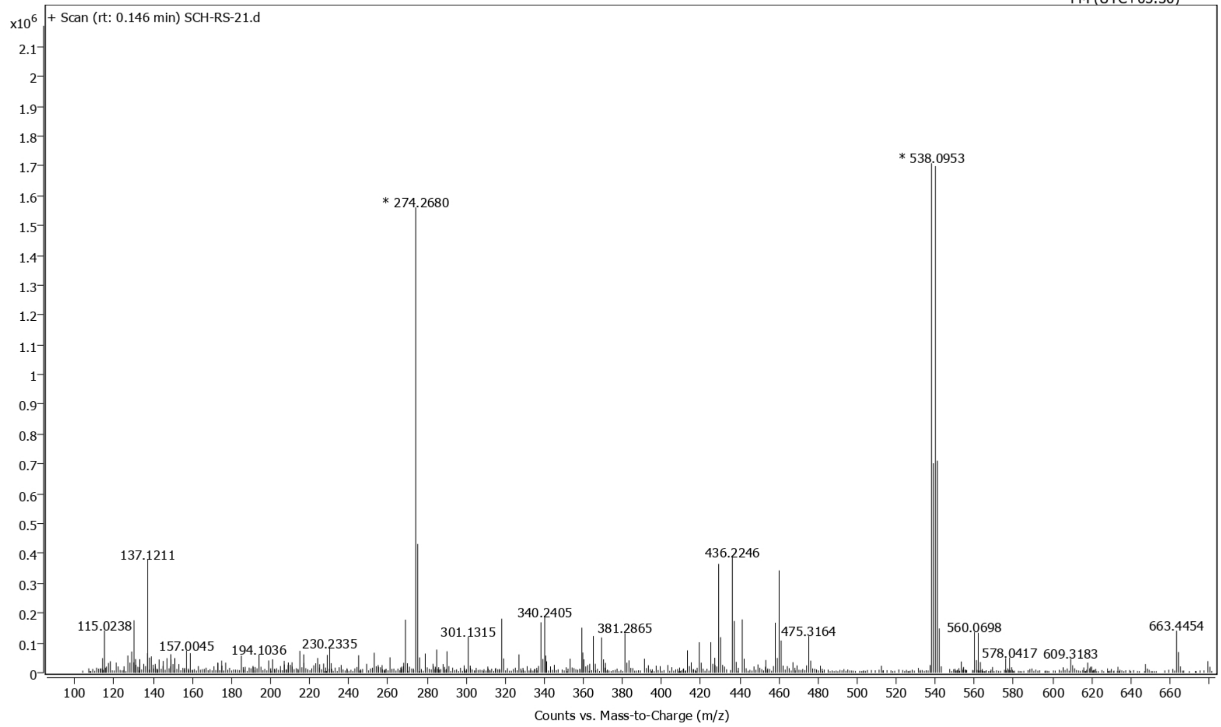

Generated at 07.50 PM on 10-08-2023

Mass Spectrum of 4m

User Spectrum Plot Report

Agilent Trusted Answers

Name	SCH-05-72	Rack Pos.		Instrument	LCMS	Operator	
Inj. Vol. (ul)	2	Plate Pos.		IRM Status	Success		
Data File	SCH-05-72.d	Method (Acq)	General Method NW.m	Comment		Acq. Time (Local)	10-08-2023 4.02.49 PM (UTC+05:30)

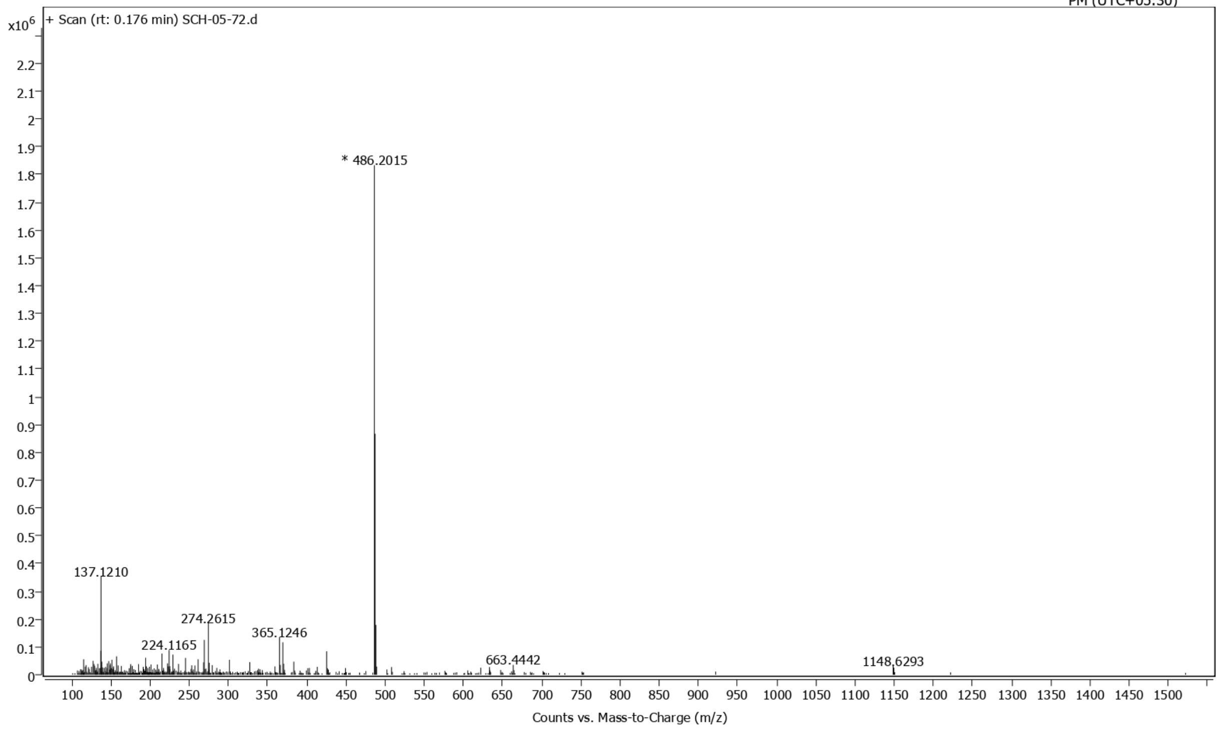

Page 1 of 1

Generated at 07.39 PM on 10-08-2023

Mass Spectrum of 4n

User Spectrum Plot Report

Name	SCH-05-73	Rack Pos.		Instrument	LCMS	Operator	
Inj. Vol. (ul)	2	Plate Pos.		IRM Status	Success		
Data File	SCH-05-73.d	Method (Acq)	General Method NW.m	Comment		Acq. Time (Local)	11-08-2023 5.14.26 PM (UTC+05:30)

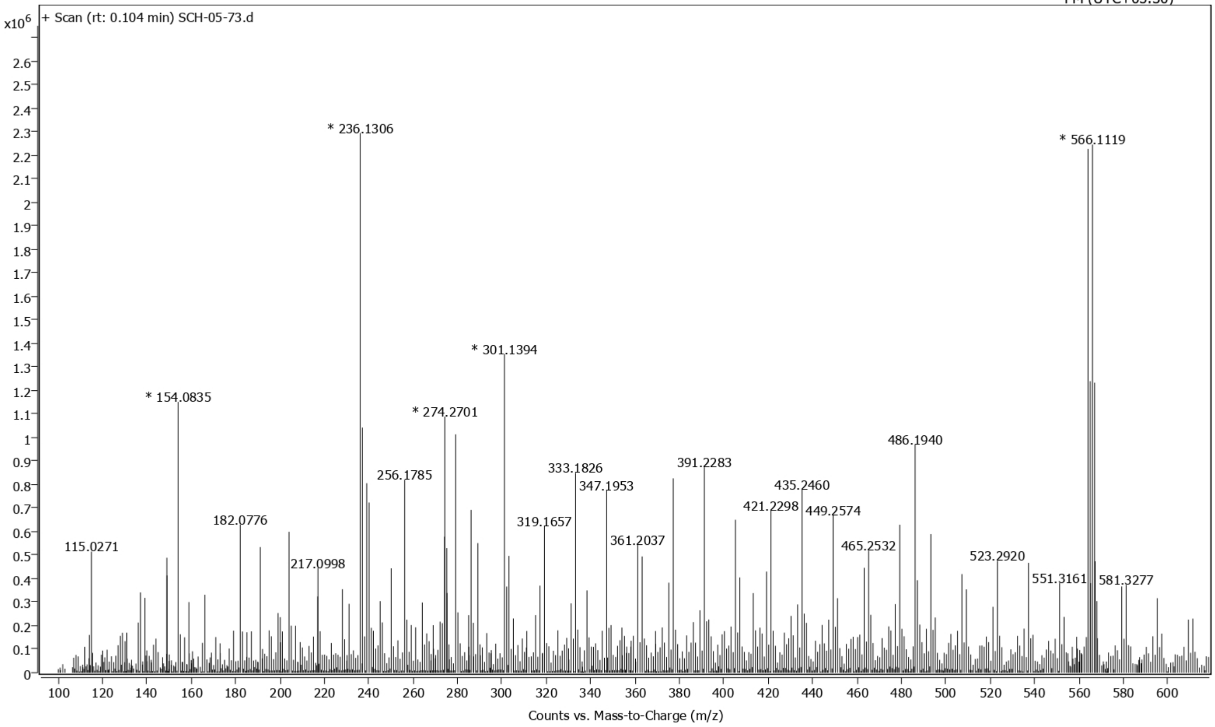

Page 1 of 1

Generated at 10.29 AM on 12-08-2023

Mass Spectrum of 4o

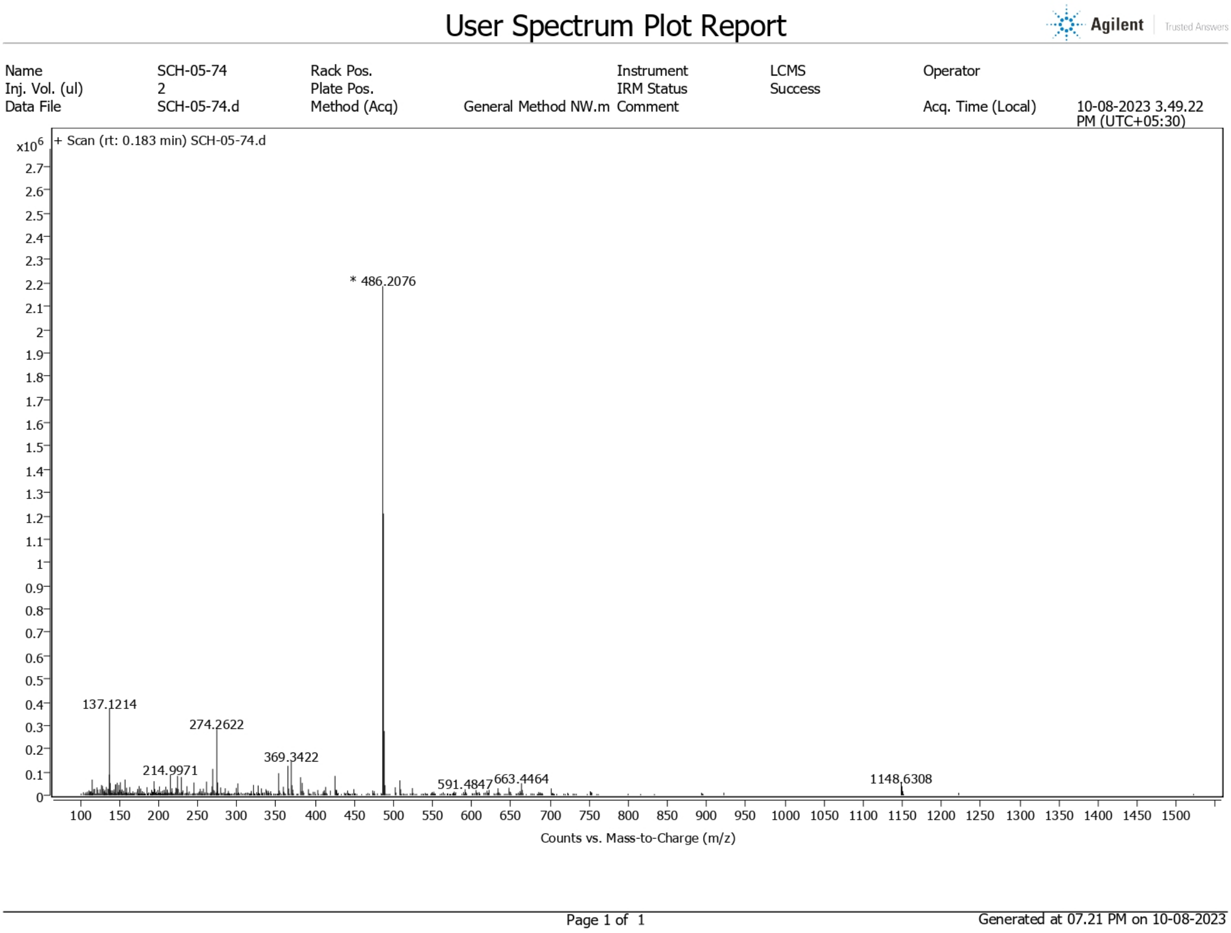

Mass Spectrum of 4p

User Spectrum Plot Report

Agilent Trusted Answers

Name	SCH-75	Rack Pos.		Instrument	LCMS	Operator	
Inj. Vol. (ul)	2	Plate Pos.		IRM Status	Success		
Data File	SCH-75D.d	Method (Acq)	General Method NW.m	Comment		Acq. Time (Local)	13-08-2023 09:04:27 (UTC+05:30)

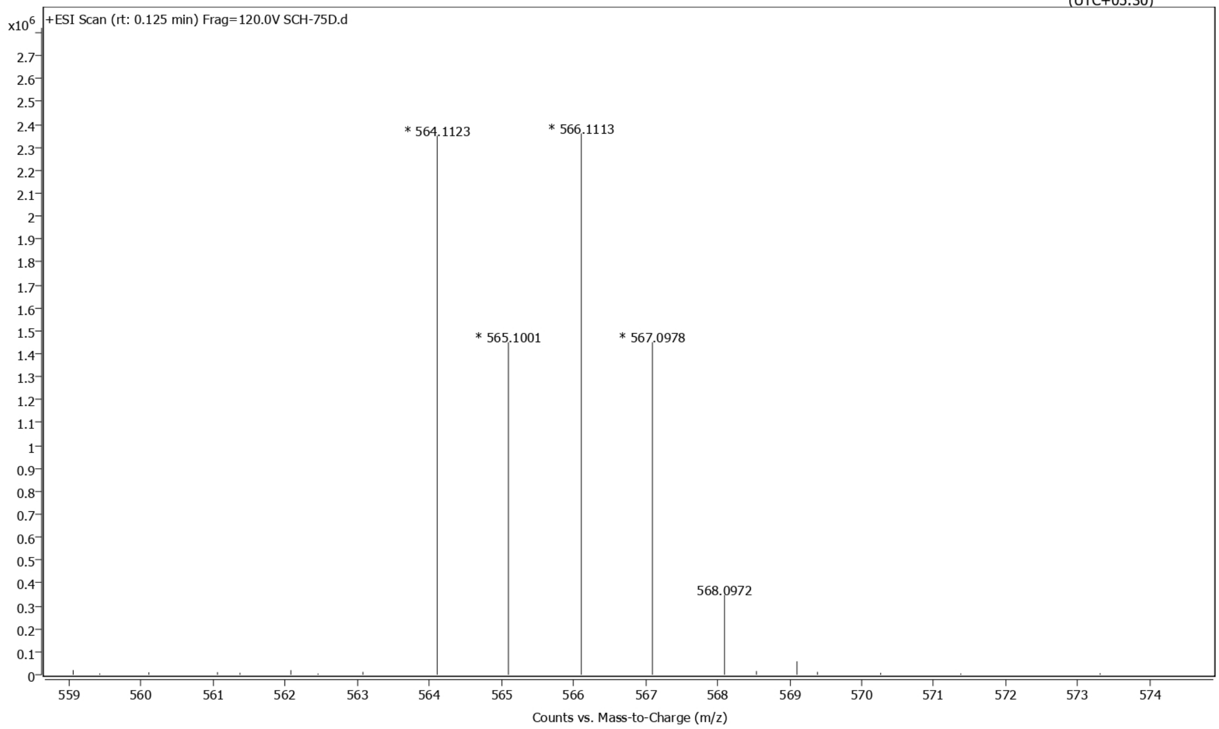

Generated at 09:09 on 13-08-2023

Mass Spectrum of 4q

User Spectrum Plot Report

Name	SCH-05-76	Rack Pos.		Instrument	LCMS	Operator	
Inj. Vol. (ul)	2	Plate Pos.		IRM Status	Success		
Data File	SCH-05-76.d	Method (Acq)	General Method NW.m	Comment		Acq. Time (Local)	11-08-2023 5.10.28 PM (UTC+05:30)

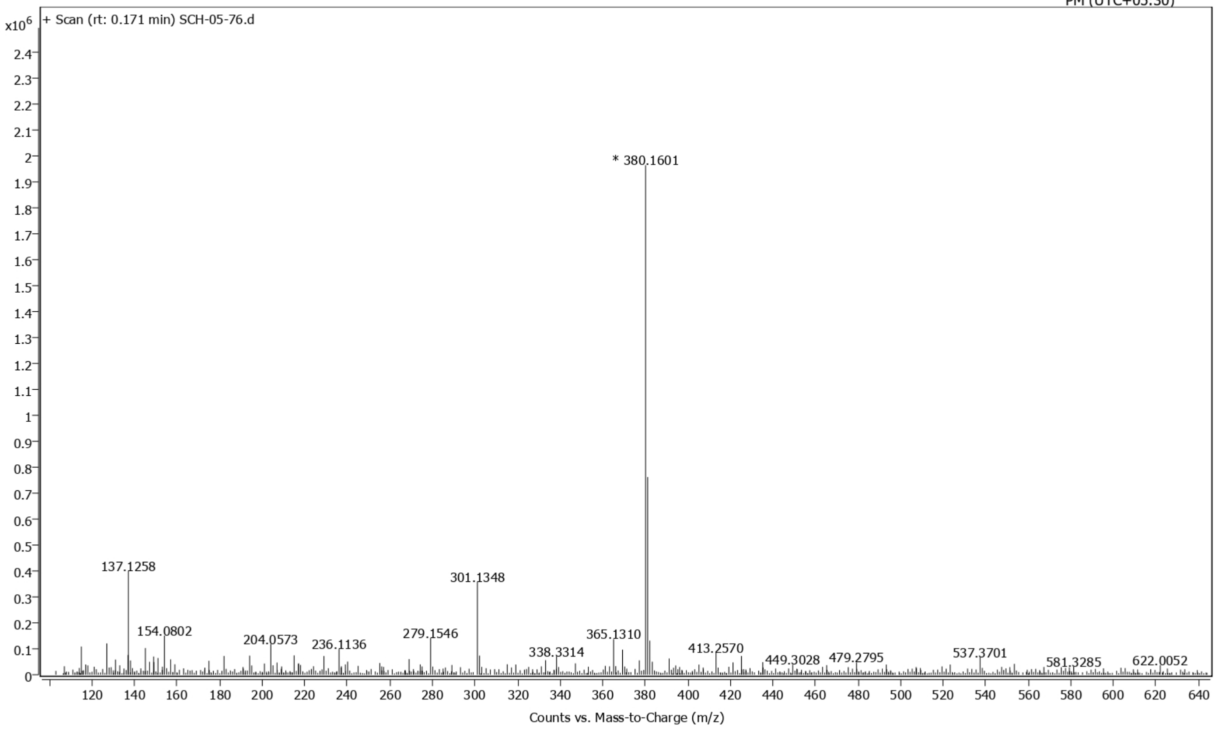

Page 1 of 1 Generated at 10.33 AM on 12-08-2023

Mass Spectrum of 4r

User Spectrum Plot Report

Name	SCH-86	Rack Pos.		Instrument	LCMS	Operator	
Inj. Vol. (ul)	2	Plate Pos.		IRM Status	Success		
Data File	SCH-77B.d	Method (Acq)	General Method NW.m	Comment		Acq. Time (Local)	13-08-2023 10:36:09 (UTC+05:30)

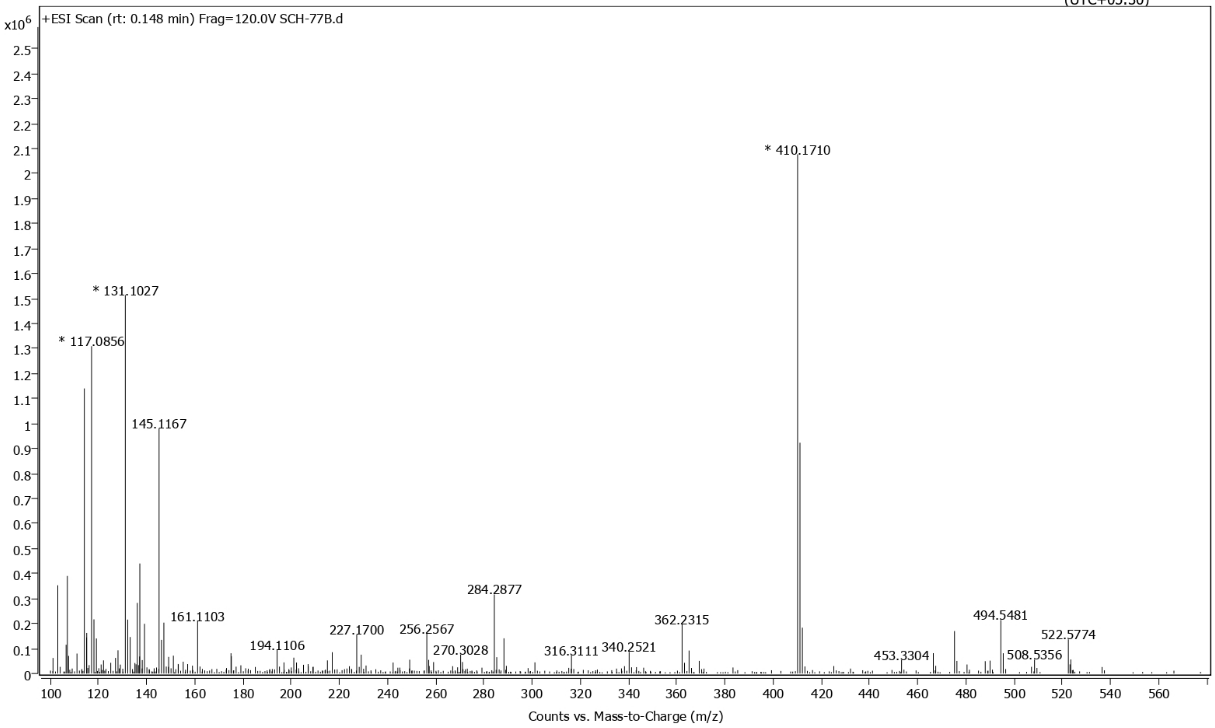

Page 1 of 1

Generated at 10:40 on 13-08-2023

Mass Spectrum of 4s

User Spectrum Plot Report

Name	SCH-05-78	Rack Pos.		Instrument	LCMS	Operator	
Inj. Vol. (ul)	2	Plate Pos.		IRM Status	Success		
Data File	SCH-05-78.d	Method (Acq)	General Method NW.m	Comment		Acq. Time (Local)	10-08-2023 3.53.12 PM (UTC+05:30)

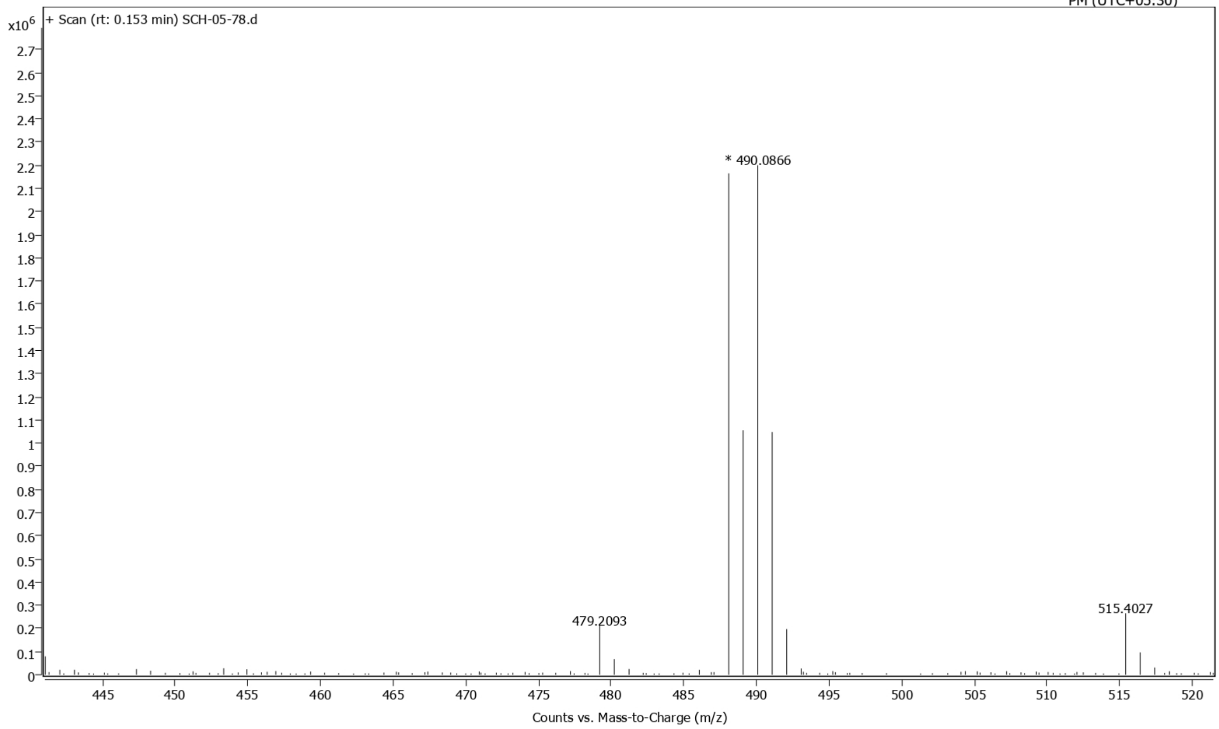

Page 1 of 1

Generated at 07.27 PM on 10-08-2023

Mass Spectrum of 4t

User Spectrum Plot Report

Agilent Trusted Answers

Name	SCH-05-79	Rack Pos.		Instrument	LCMS	Operator	
Inj. Vol. (ul)	2	Plate Pos.		IRM Status	Success		
Data File	SCH-05-79.d	Method (Acq)	General Method NW.m	Comment		Acq. Time (Local)	10-08-2023 4.06.40 PM (UTC+05:30)

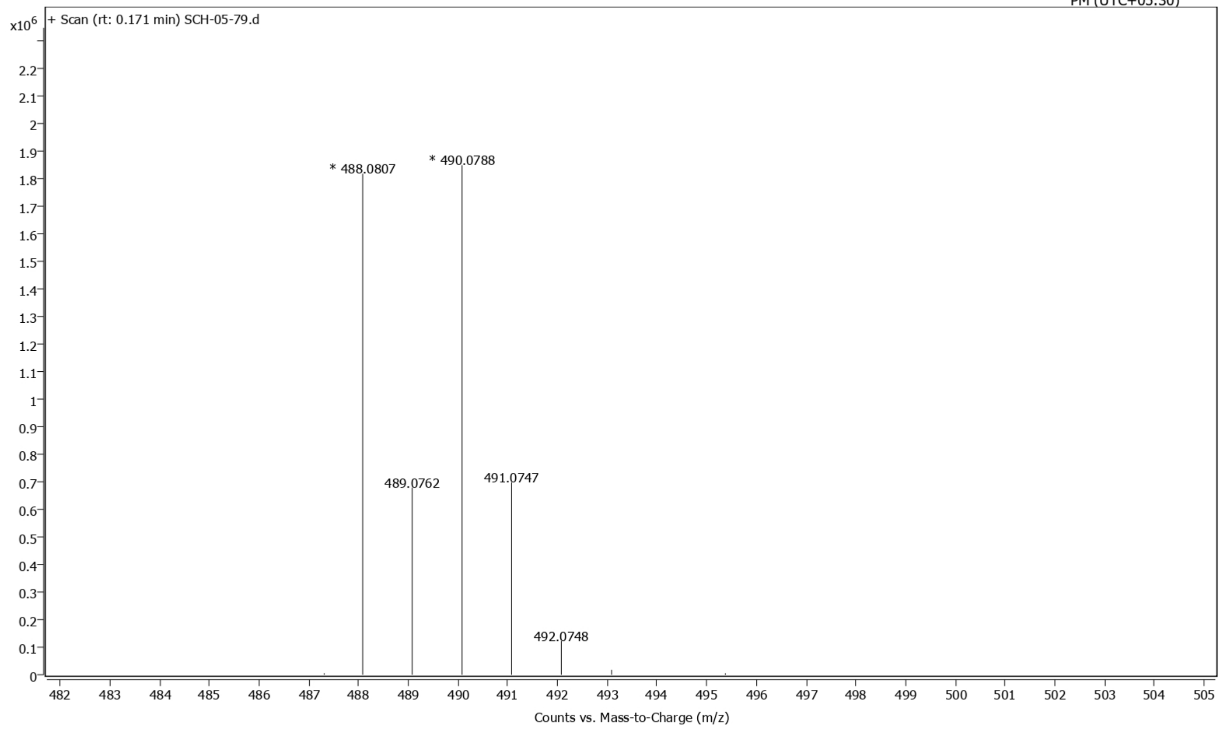

Page 1 of 1

Generated at 03.33 PM on 11-08-2023

Mass Spectrum of 4u

User Spectrum Plot Report

Name	SCH-86A	Rack Pos.		Instrument	LCMS	Operator	
Inj. Vol. (ul)	2	Plate Pos.		IRM Status	Success		
Data File	SCH-86B.d	Method (Acq)	General Method NW.m	Comment		Acq. Time (Local)	13-08-2023 08:29:47 (UTC+05:30)

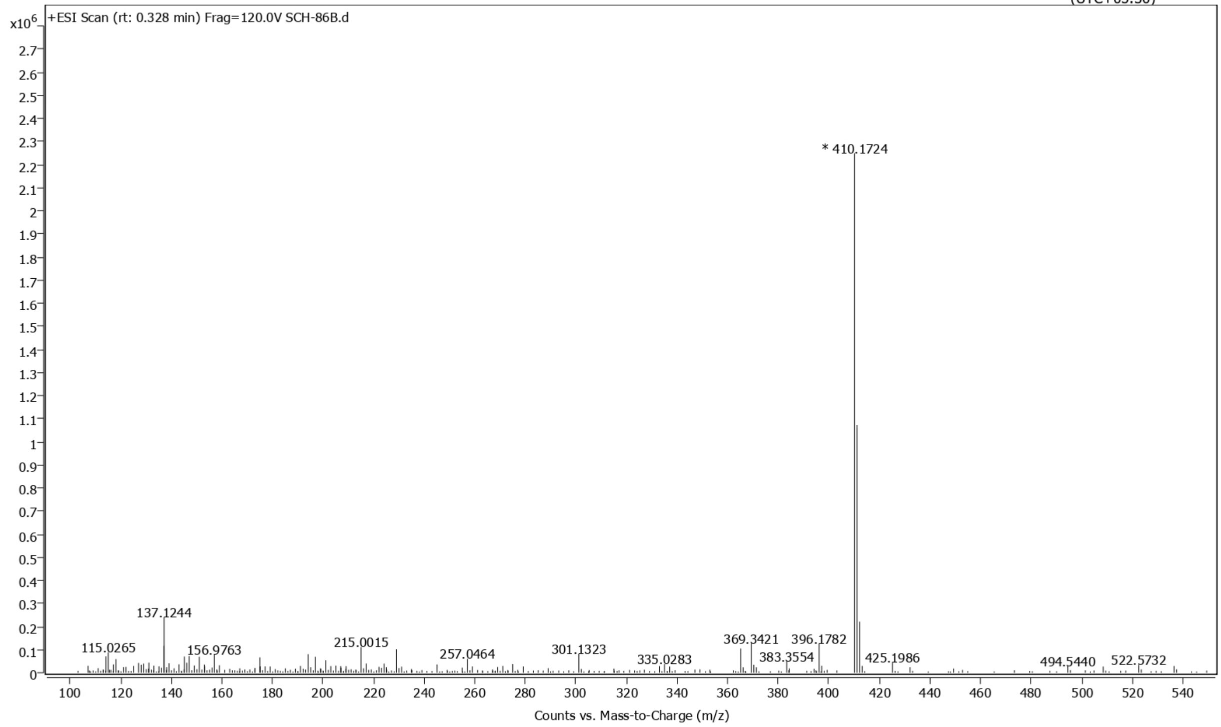

 Generated at 08:37 on 13-08-2023

Mass Spectrum of 4v

User Spectrum Plot Report

Agilent Trusted Answers

Name	SCH-87	Rack Pos.		Instrument	LCMS	Operator	
Inj. Vol. (ul)	2	Plate Pos.		IRM Status	Success		
Data File	SCH-87G.d	Method (Acq)	General Method NW.m	Comment		Acq. Time (Local)	13-08-2023 09:29:30 (UTC+05:30)

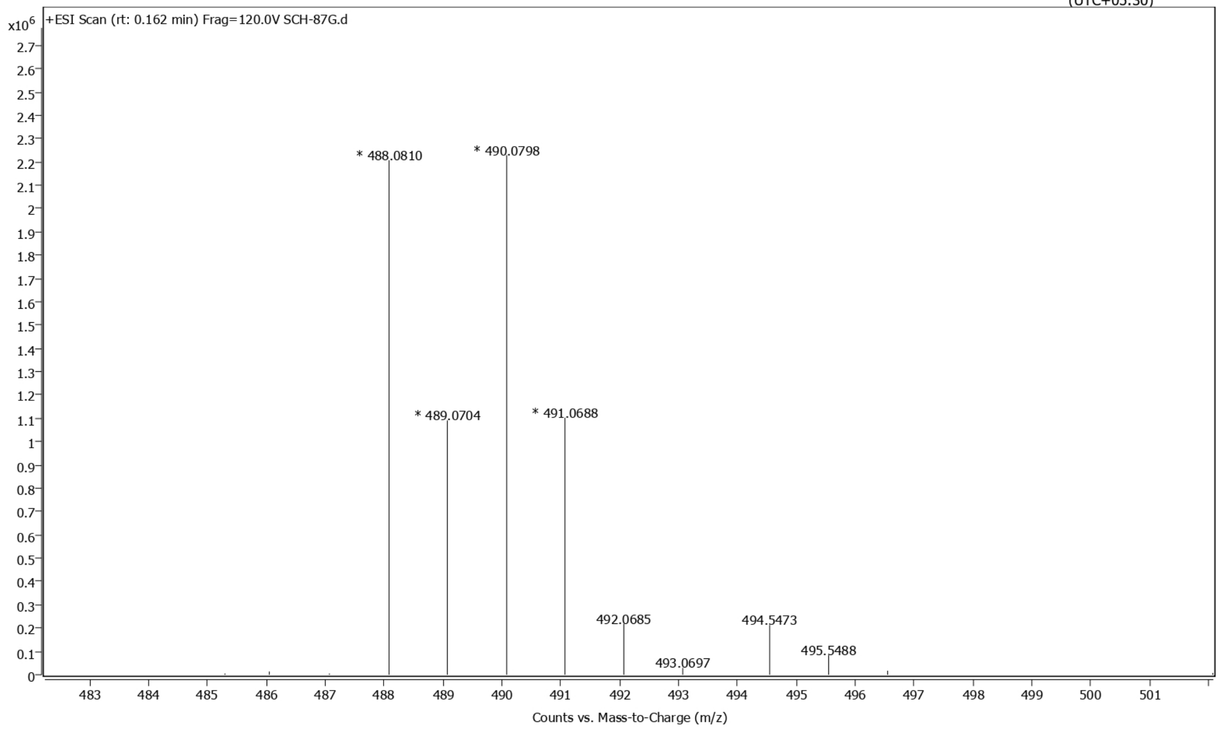

Page 1 of 1 Generated at 09:32 on 13-08-2023

Mass Spectrum of 4w

Figure 2: Cytotoxicity against A549 cell lines.

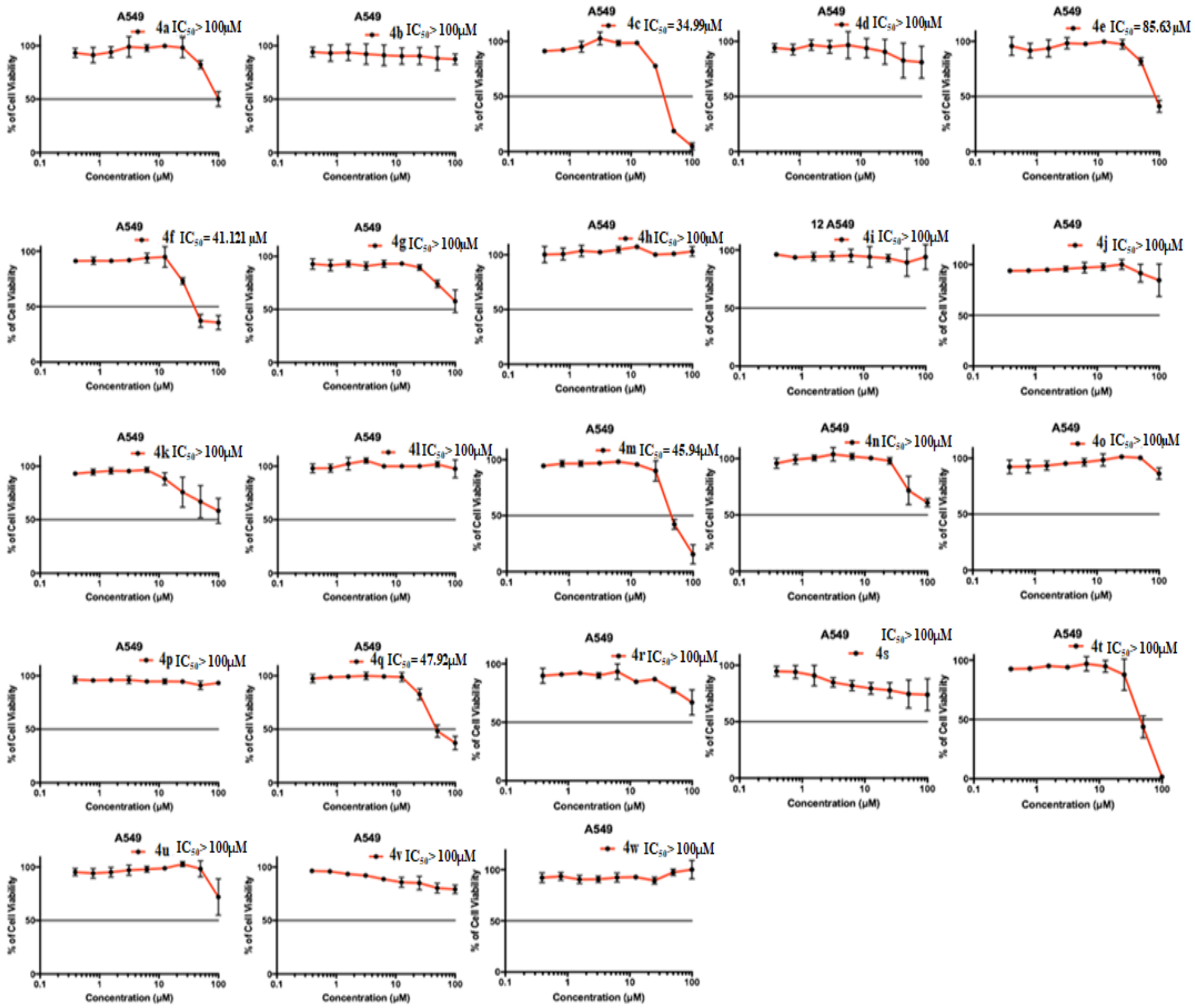

Figure 3: Cytotoxicity against BEAS2B cell lines.

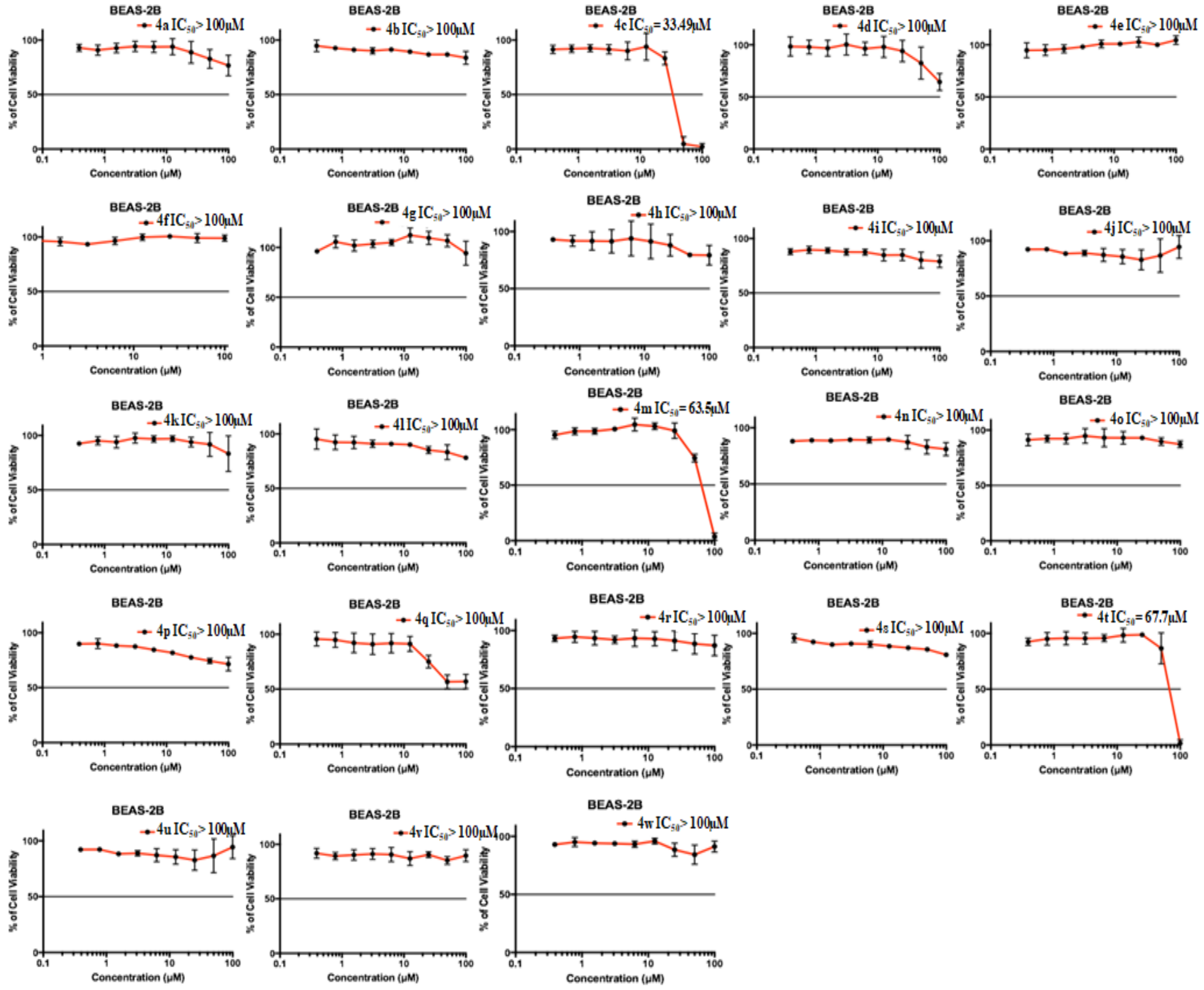

Figure 4: Cytotoxicity against HepG2 cell lines.

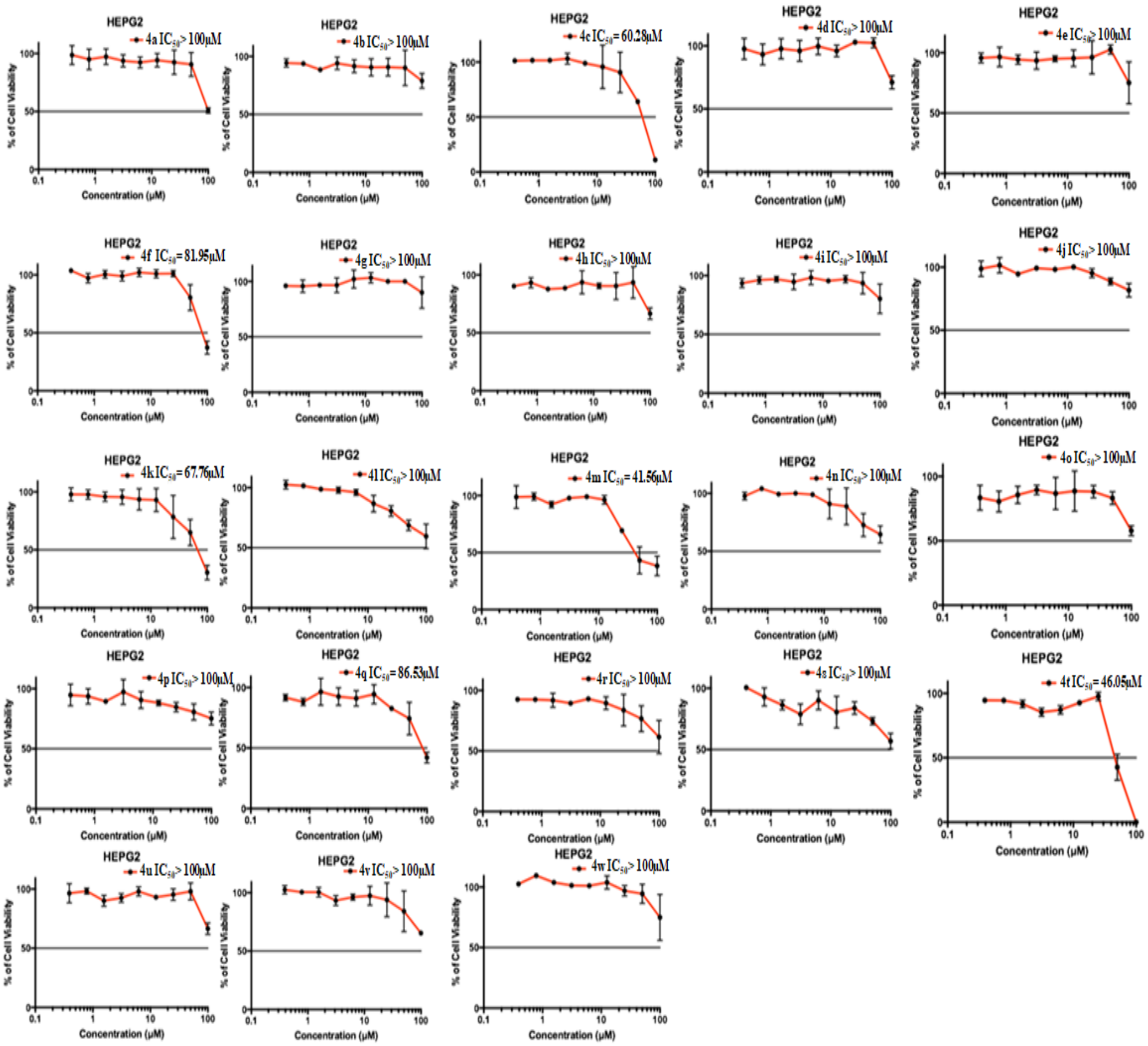

Figure 5: Cytotoxicity against LO2 cell lines.

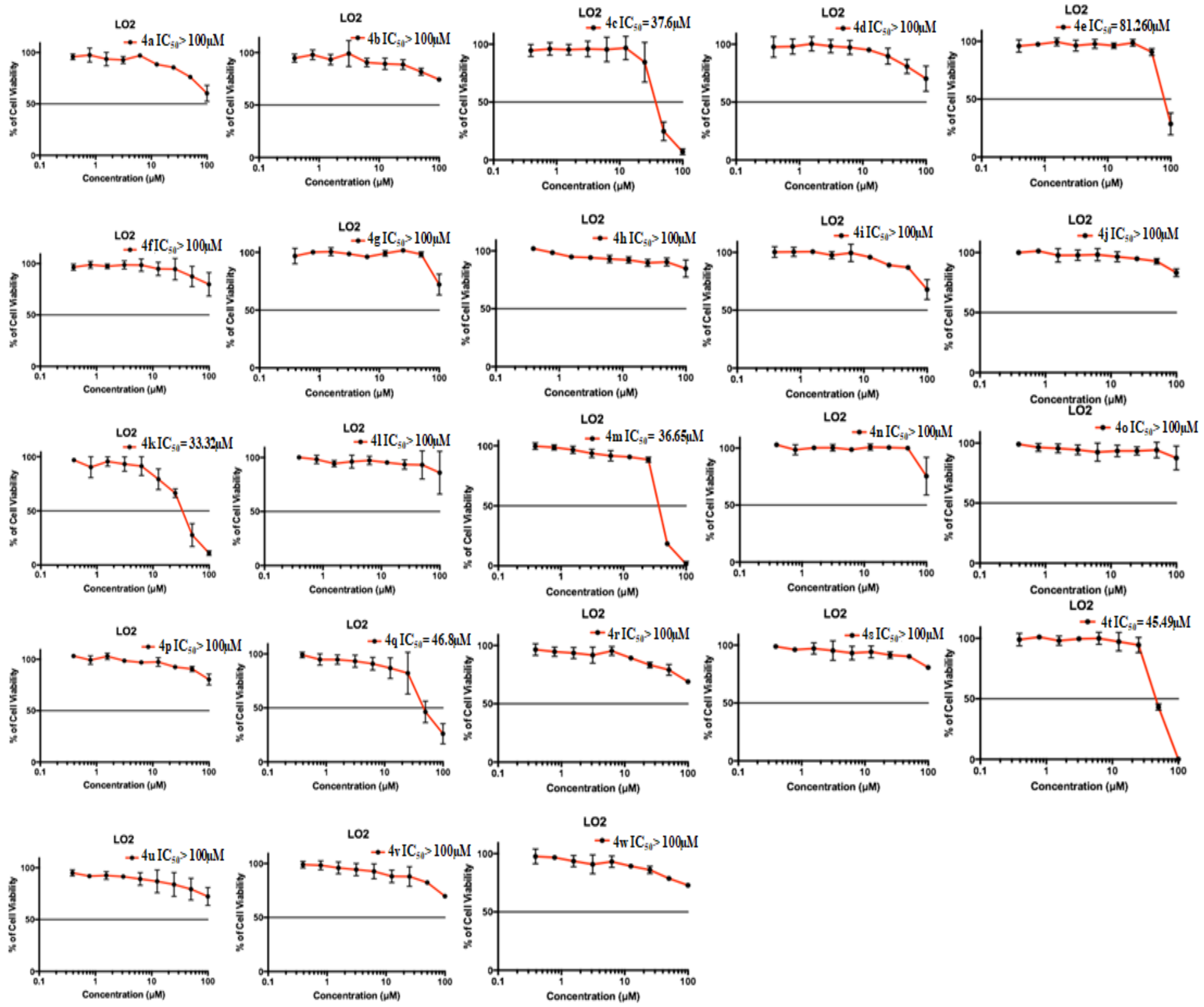

Supplement: Supplementary file 1 [file molecules-28-06503-s001.zip › molecules-2509588-supplementary.pdf]
